# Exploring and Targeting the Connection of Iron and Copper Homeostasis to Neurodegenerative Diseases

**DOI:** 10.1002/mco2.70766

**Published:** 2026-05-18

**Authors:** Xin Liu, Lin Jia, Kerong Wu, Mo Chen, Jichao Sun, Chuanbin Yang, Chengchao Xu, Jie Sun, Jigang Wang, Lingyun Dai

**Affiliations:** ^1^ Department of Geriatrics, Guangdong Provincial Clinical Research Center for Geriatrics, Shenzhen Clinical Research Center for Geriatrics Shenzhen People's Hospital (The First Affiliated Hospital, Southern University of Science and Technology; The Second Clinical Medical College, Jinan University) Shenzhen China; ^2^ Faculty of Pharmaceutical Sciences Shenzhen University of Advanced Technology Shenzhen China; ^3^ Department of Urology The First Affiliated Hospital of Ningbo University Ningbo Zhejiang China; ^4^ Laboratory of Oral Homeostatic Medicine, School of Medicine and SUSTech Homeostatic Medicine Institute (SHMI) Southern University of Science and Technology Shenzhen China; ^5^ Department of Pharmacology, Joint Laboratory of Guangdong‐Hong Kong Universities for Vascular Homeostasis and Diseases, School of Medicine and SUSTech Homeostatic Medicine Institute (SHMI) Southern University of Science and Technology Shenzhen China; ^6^ State Key Laboratory for Quality Ensurance and Sustainable Use of Dao‐di Herbs, Artemisinin Research Center and Institute of Chinese Materia Medica China Academy of Chinese Medical Sciences Beijing China; ^7^ Ningbo Key Laboratory of Nervous System and Brain Function, Department of Neurosurgery The First Affiliated Hospital of Ningbo University Ningbo Zhejiang China

**Keywords:** intrinsically disordered proteins, iron and copper homeostasis, metal‐binding proteins, neurodegenerative diseases, therapeutic agents

## Abstract

Iron (Fe) and copper (Cu) are vital micronutrients that regulate many critical physiological processes in the human body, with their homeostasis in the central nervous system (CNS) being essential for proper neuronal function. Disruptions in their metabolism and regulatory pathways have been associated with the pathogenesis of various forms of neurodegenerative diseases (NDDs) such as Alzheimer's disease (AD) and Parkinson's disease (PD). Despite growing research on metal homeostasis, the intricate molecular mechanisms that link iron and copper metabolism to the initiation and progression of NDDs remain insufficiently elucidated. In this review, we provide a systematic overview of the metabolic processes of iron and copper in the body and CNS, highlighting their interactions with many metal‐binding proteins, including transporters, storage proteins, and important intrinsically disordered proteins (e.g., amyloid β‐protein, tau, and alpha‐synuclein) involved in NDDs. We further dissect the downstream effects of metal ion dyshomeostasis on cellular redox balance, neuroinflammation, autophagy, organelle interaction network, and cell death. Additionally, we discuss current therapeutic strategies aimed at targeting iron and copper dyshomeostasis, as well as the emerging role of artificial intelligence in this field of research. By integrating metal metabolism, metal–protein interactions, the effect of metal dyshomeostasis on downstream biological processes, and potential intervention strategies, this review serves as a comprehensive reference for understanding the pathogenesis of NDDs and offers new perspectives for developing effective therapeutics. Overall, this review underscores the significance of reinstating metal balance for the treatment of neurodegeneration.

## Graphical abstract

Iron and copper dyshomeostasis, along with their interactions with key intrinsically disordered proteins (e.g., Aβ, tau, α‐synuclein) have a strong implication in the onset and progression of neurodegenerative diseases, including Alzheimer's disease (AD), Parkinson's disease (PD), Prion diseases (PrDs), Huntington's disease (HD), Wilson's disease (WD), Menkes disease (MD), Amyotrophic lateral sclerosis (ALS), and Multiple sclerosis (MS). The central mechanisms at play involve iron‐ or copper‐catalyzed oxidative stress, neuroinflammation, dysregulated autophagy, dysfunction of the organelle interaction network (particularly in the mitochondria–lysosome and endoplasmic reticulum–Golgi axes), and different cell death pathways (such as apoptosis, pyroptosis, ferroptosis, and cuproptosis). Multiple therapeutic agents, including iron/copper chelators, iron/copper ionophores, natural products, and other multifunctional compounds, highlight the potential of targeting iron or copper dyshomeostasis as a promising approach for treating neurodegenerative diseases.

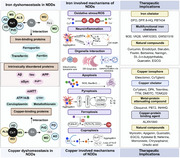

## 1 Introduction

Neurodegenerative diseases (NDDs) represent a significant challenge in the world. They are characterized by the gradual deterioration of neuronal function, resulting in a range of symptoms that include memory loss, motor impairment, and cognitive decline [1]. The development of NDDs results from a complex interplay of genetic, environmental, and cellular factors, with aging being the primary risk factor for most NDDs [2]. Additionally, accumulating studies have highlighted the critical roles of protein misfolding and aggregation, mitochondrial dysfunction, oxidative stress, neuroinflammation, and various formats of cell death in the pathogenesis of NDDs [3].

In recent years, the role of iron and copper homeostasis in maintaining brain health has garnered increasing attention [4, 5]. Accumulating evidence suggests that disruptions in iron and copper homeostasis or long‐term exposure to an iron‐ or copper‐polluted environment can result in different forms of NDDs [6, 7]. Iron and copper are indispensable trace elements that underpin numerous fundamental life processes. As key cofactors for various proteins and enzymes, they facilitate many essential biological functions, including mitochondrial respiration, antioxidant responses, angiogenesis, wound healing, and neuromodulator biosynthesis [6, 8].

The diverse range of biological activities in which iron and copper are involved depends largely on the various iron‐binding proteins (FeBPs) and copper‐binding proteins (CuBPs) in which Fe or Cu acts as a cofactor or structural component. Among various FeBPs and CuBPs, other than the transporters, chaperones, and storage proteins involved in the physiological transportation and distribution of metal ions, the interactions between intrinsically disordered proteins (IDPs) and metal ions hold significant relevance within the realm of NDDs. IDPs lack a single stable and cooperatively folded structure under physiological conditions [9]. They are highly flexible and dynamic, participating in numerous cellular processes. Emerging evidence suggests that excess iron and copper can lead to misfolding and aggregation of certain IDPs, including β‐amyloid (Aβ), tau, alpha‐synuclein (αSyn), prion protein (PrP), and mutant huntingtin protein (mHTT), contributing to the pathogenesis of NDDs. However, the effects of iron and copper on IDPs are complicated, and the underlying molecular mechanisms are not fully understood.

This review focuses on the intricate and complex roles of iron and copper homeostasis within the central nervous system (CNS) in relation to the pathogenesis of NDDs. First, we outline the metabolic processes of iron and copper within both the organism and the CNS. We then evaluate the biological functions of proteins that interact with these metal ions, with an emphasis on the biochemical aspects such as the protein motifs and amino acid sites involved in iron/copper binding. Next, we discuss the downstream effects of iron and copper dyshomeostasis on the various biological processes, including cellular redox balance, neuroinflammation, autophagy, organelle interaction network, and cell death, followed by the dissection of the molecular mechanisms associated with iron and copper dyshomeostasis in the context of eight key types of NDDs. Furthermore, we summarize the current therapeutic strategies for iron‐ or copper‐targeted intervention in NDDs, ranging from small‐molecule drugs to natural products. Finally, we also address the role of artificial intelligence (AI) technologies in this domain, including machine learning (ML)‐based predictions of metal‐binding sites (MBSs), virtual screenings (VSs) for novel iron/copper modulators, big data analytics for metal‐related genetic variants, and neuroimaging techniques for monitoring brain metal levels, thereby highlighting their emerging roles in accelerating research progress and uncovering novel mechanisms connecting iron and copper homeostasis to NDDs.

## 2 The Complexity and Dynamics of Iron and Copper Metabolism

This section presents a comprehensive analysis of iron and copper metabolism, tracing these essential metals journey from fundamental physiological regulation to their pivotal roles in neurodegenerative pathology. It begins with an examination of the systemic and cellular “life cycles” of iron and copper, highlighting their specialized homeostasis mechanisms within the CNS. To provide a molecular basis, the biochemical interactions between these metal ions and their primary transporters and storage proteins are detailed. A central focus is then placed on the structural and functional interactions between iron/copper and key IDPs, such as Aβ, tau, and αSyn, which are characteristic features of various NDDs. Finally, the discussion expands to the complex downstream consequences of metal dyshomeostasis, including oxidative stress, neuroinflammation, autophagic impairment, and the induction of various cell death pathways.

### 2.1 The Life Cycle of Iron and Copper Ions Within the Human Body

**FIGURE 1:**
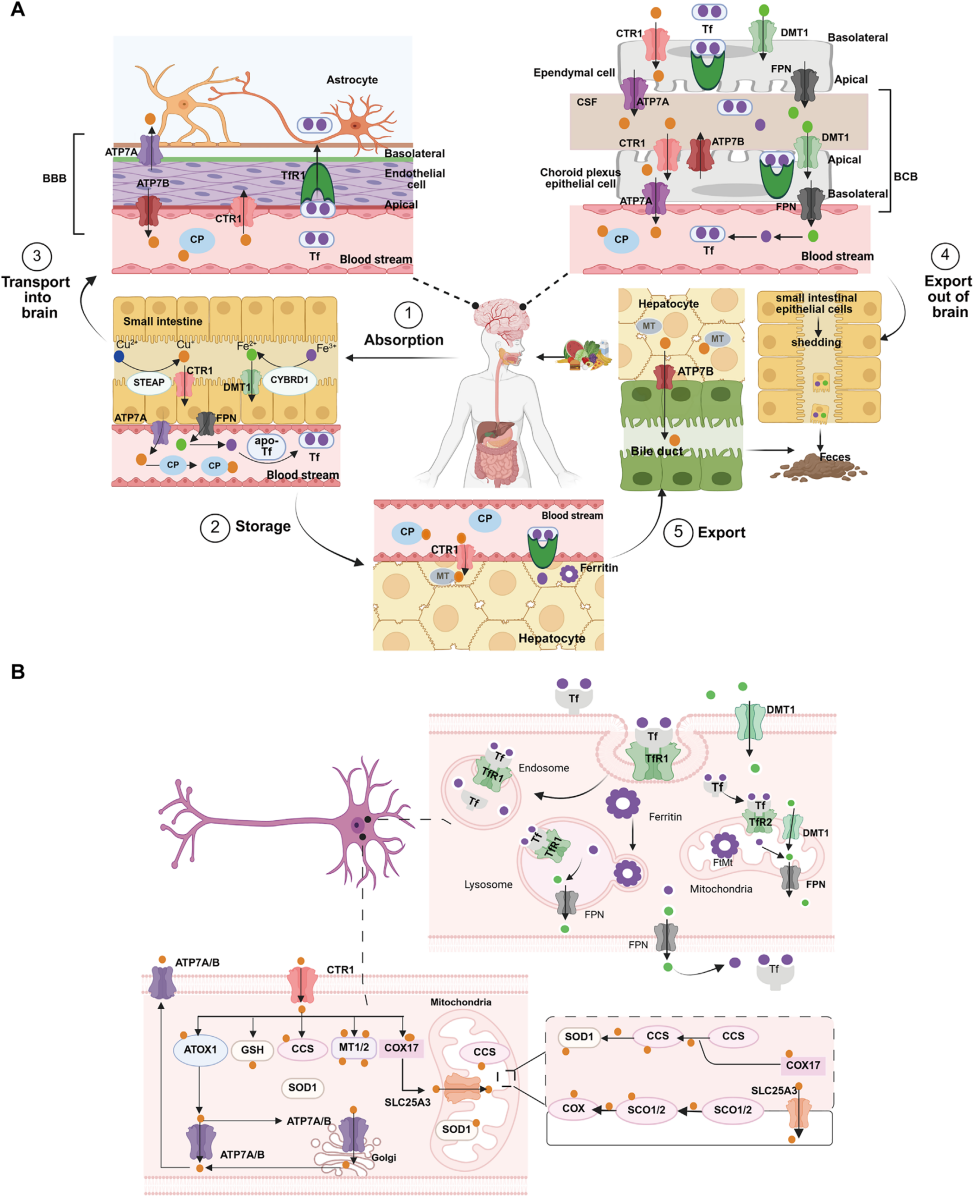
The life cycle of iron and copper within systemic circulation and their metabolism in the CNS. (A) (1) **Iron**: Intestinal cells reduce Fe^3^^+^ to Fe^2^^+^ via CYBRD1 and absorb it via DMT1. The absorbed iron is exported into blood by FPN, reoxidized to Fe^3^^+^, and bound to Tf for systemic delivery. Iron enters tissues via Tf–TfR1 endocytosis, is stored in the ferritin protein of the liver, and crosses the BBB via TfR1‐mediated uptake. Clearance of iron from the brain may involve BCB. (2) **Copper**: Dietary Cu^2^^+^ is reduced to Cu^+^ by STEAP1 and taken up into intestinal cells via CTR1. It enters the blood via ATP7A, binds to Cp, and is delivered to the liver. In the brain, copper crosses the BBB via CTR1 and ATP7A on endothelial cells. Astrocytes take up copper using CTR1 and facilitate copper supply to neurons. ATP7B in the choroid plexus may mediate copper entry into CSF, while ATP7A facilitates its efflux back to the blood. Excess copper is excreted into bile. (B) (1) **Iron**: Iron bound to Tf enters cells via TfR1‐mediated endocytosis. Within the endosome, iron is released. A portion of the released iron is stored in ferritin, while another portion is transported to organelles such as mitochondria via DMT1. Excess iron is exported from the cell by FPN. After iron release, the Tf–TfR1 complex is recycled to the cell membrane, restarting the uptake cycle and thereby maintaining cellular iron homeostasis. (2) **Copper**: Upon entering the cytoplasm, copper ions bind to GSH, MTs, or specific chaperones, including CCS, ATOX1, and COX17. CCS delivers copper to SOD1, while ATOX1 transports it to ATP7A/B for secretion. For mitochondrial utilization, COX17 and SLC25A3 mediate copper transport across the outer and inner membranes, respectively. Subsequently, SCO1/2 deliver copper to COX in the intermembrane space.

Iron and copper serve as indispensable cofactors for a multitude of enzymes, playing a critical role in their catalytic activities; however, an excess of iron or copper can be detrimental to cells, leading to cell death. The body maintains iron and copper homeostasis through a delicate balance of absorption, transport, storage, and excretion mechanisms (Figure 1A).

#### 2.1.1 Iron Homeostasis Within the Human Body

Dietary iron primarily exists in two forms: heme iron and nonheme iron. Heme iron, derived from animal‐based foods, is the most efficiently absorbed form [10]. Although the absorption mechanism of heme iron remains incompletely understood, its iron atom ferrous iron (Fe^2^^+^), tightly bound within a porphyrin ring, is liberated by heme oxygenase 1 (HMOX1) in the endoplasmic reticulum (ER) after uptake by enterocytes, subsequently entering the cytoplasmic labile iron pool (LIP) [11]. Nonheme iron, predominantly in the ferric (Fe^3^^+^) state, is widely distributed in plant‐based foods. Enterocytes must reduce Fe^3^^+^ to Fe^2^^+^ for absorption, a process catalyzed by ascorbic acid or the plasma membrane ascorbate‐dependent reductase CYBRD1 [12]. The reduced Fe^2^^+^ is then transported across the intestinal brush border membrane (BBM) via divalent metal transporter 1 (DMT1, encoded by *SLC11A2*) [13].

Iron enters the cytoplasmic LIP of enterocytes, where it can be utilized, stored, or exported. Poly(rC)‐binding proteins (PCBPs) maintain iron in a stable, inactive state and direct its transport to different targets [14]. Depending on systemic iron demand, iron is either stored or exported: when systemic demand is low, PCBP1/2 delivers iron to the primary cellular iron‐storage protein ferritin for storage [15]. When iron demand is high, iron is transported to the basolateral membrane (BLM) and is released into the bloodstream via the sole mammalian iron exporter ferroportin (FPN, encoded by *SLC40A1*) [16]. This export process also relies on the membrane‐anchored multicopper ferroxidase hephaestin (HEPH), which oxidizes the exported Fe^2^^+^ to Fe^3^^+^ [17]. The newly released Fe^3^^+^ then binds to apotransferrin (apo‐Tf) in the portal circulation [18]. In addition, another copper‐dependent ferroxidase, ceruloplasmin (Cp), also functions in facilitating iron release from storage sites such as the liver and oxidizing Fe^2^^+^ to Fe^3^^+^ so it can bind to apo‐Tf for systemic distribution.

Intracellular iron handling occurs in several key organelles (Figure 1B). The lysosome is a central site for iron storage and recycling [19]. Within the acidic and reducing environment of lysosomes, Fe^3^^+^ is reduced to Fe^2^^+^, and then released into the cytosol through channels like DMT1 and mucolipin‐1 (MCOLN1) [19]. Mitochondria are crucial for iron utilization. Iron transport across the outer mitochondrial membrane involves the voltage‐dependent anion channel (VDAC), Tf‐TfR2, and DMT1. Within the mitochondrial matrix, iron is utilized to synthesize iron–sulfur (Fe–S) clusters and heme or is stored in mitochondrial ferritin [20].

#### 2.1.2 Copper Homeostasis Within the Human Body

Copper ions are primarily transported in the bloodstream through their association with proteins rather than being present in a free state [21]. Approximately 75% of copper ions are nonexchangeable complexed with Cp, with roughly 25% being in an exchangeable complex with human serum albumin. A tiny fraction, approximately 0.2%, is bound to histidine (His) [21]. It is crucial to recognize that unbound copper, due to its strong oxidative properties, can act as a catalyst in the formation of highly reactive hydroxyl radicals. This can lead to significant cellular damage by inducing oxidative stress on DNA, proteins, and lipids [22]. The absorption of copper is mainly mediated by the copper transporter 1 (CTR1, also known as SLC31A1), which transports copper across the apical membrane of the small intestine epithelial cells. Copper is then delivered to the basolateral side of the epithelial cells through copper chaperone antioxidant 1 (ATOX1), followed by exporting into the bloodstream via the copper‐transporting ATPase 1 (ATP7A) protein [23]. From there, copper is transported to the liver via the portal vein [24]. The liver acts as the principal organ for copper storage and excretion. Copper storage is thought to be mediated by metallothionein‐1 and 2 (MT1/2), while excretion is mainly managed by copper‐transporting ATPase 2 (ATP7B), which excretes copper into bile [25].

Before CTR1 can uptake copper ions, the cupric ion Cu^2+^ is reduced to the monovalent cuprous ion Cu^+^ by the metalloreductases from the six‐transmembrane epithelial antigen of the prostate (STEAP) family [26]. Cu^+^ then binds to the ectodomain of CTR1 and enters the cell in its reduced state (Figure 1B). Cu^+^ passes from the mitochondrial membrane and enters the mitochondrial matrix via solute carrier family 25 member 3 (SLC25A3) [27], although the precise mechanism remains elusive [28].

Intracellular chelators such as MT1/2 and glutathione (GSH) protect cells from damage induced by excess free copper [29]. Copper chaperone proteins ensure the proper distribution and function of copper within the cell [30]. ATOX1, for example, binds Cu^+^ and delivers it to ATP7A for cellular export. The copper chaperone for superoxide dismutase (CCS) directly interacts with copper/zinc superoxide dismutase (SOD1), facilitating the transport of copper ions [31]. CCS also promotes the disulfide bond formation in SOD1, which is crucial for the latter's structure and function [32, 33]. SOD1 is vital for catalyzing the conversion of superoxide radicals to H_2_O_2_ and maintaining intracellular redox homeostasis, and the inactivation of SOD1 can trigger cell death [34].

A range of copper chaperone proteins in mitochondria are involved in the assembly and function of cytochrome C oxidase (COX), a key component of the mitochondrial respiratory chain [35]. Cytochrome C oxidase copper chaperone (COX17) transports Cu^+^ from the cytoplasm to the mitochondrial intermembrane space [36], where it is further delivered to the synthesis of cytochrome C oxidase 1 and 2 (SCO1/2), facilitating their disulfide bond formation [37]. Cytochrome C oxidase assembly factor 6 (COA6) is involved in the reduction of disulfide bonds in SCO1/2 [38], enabling their binding to Cu^+^ [39]. A deficiency in COA6 can disrupt the biosynthesis of respiratory Complex IV [40]. With the assistance of COA6, SCO1/2 deliver Cu^+^ obtained from COX17 to COX, participating in its assembly [41]. In addition, SCO1/2 also play a role in regulating cellular copper homeostasis, and their absence can lower intracellular Cu^+^ levels [42].

#### 2.1.3 Interrelation of Iron and Copper Homeostasis

Iron and copper homeostasis are closely intertwined. Several studies have revealed an inverse relationship between the iron/copper status and the hepatic metal accumulation: iron deficiency results in an increase in hepatic copper levels, although the exact mechanism behind this remains unclear [43]; conversely, copper deficiency leads to hepatic iron accumulation, likely due to reduced Cp activity under copper deprivation, which impairs iron release from hepatic stores [43].

At the molecular level, iron is capable of regulating the binding capacity of the iron regulatory protein (IRP) to the iron‐responsive element (IRE) [44]. Interestingly, copper can also influence the binding of IRP to IRE by occupying the iron‐binding site on IRP directly [45]. For example, elevated levels of copper can decrease the mRNA expression of TfR1, which contains an IRE within its 3′UTR region [46]. Moreover, excess copper can reduce the expression of the IRE‐containing DMT1 isoform while increasing the expression of FPN, which facilitates increased iron efflux from cells [47]. Additionally, copper treatment also promotes the expression of the *HAMP* gene, which encodes the iron regulatory peptide hepcidin, a hormone that promotes the endocytosis and degradation of FPN [48]. In contrast, a copper‐deficient diet decreases the activity of HEPH, a multicopper ferroxidase essential for the release of iron from intestinal epithelial cells into the bloodstream, and reduces iron absorption; both of these effects can be reversed through copper supplementation [49].

On the other hand, iron deficiency correlates with an upregulation of DMT1 mRNA and the copper transport proteins ATOX1 and ATP7A [50, 51]. Consequently, iron deficiency in rat models has been associated with an increased rate of copper clearance from the cerebrospinal fluid (CSF) [52]. The effect of iron deficiency on copper efflux may be attributable to transcriptional upregulation by hypoxia‐inducible factor‐2α (HIF‐2α) [53]. Under low‐iron conditions, HIF‐2α activation promotes the transcription of *DMT1* and *CYBRD1* to induce the synthesis of iron transport proteins, while simultaneously upregulating the mRNA expression of the copper importer CTR1 and exporter ATP7A [54]. In contrast, rodents fed with a high‐iron diet can develop copper deficiency‐related pathological symptoms, including growth retardation and anemia [55]. These results collectively support that there are close intersections between iron and copper metabolism, which have important, albeit not fully understood, physiologic and pathological implications.

### 2.2 The Role of Iron and Copper in Neuronal Function and Homeostasis

#### 2.2.1 Iron in Neuronal Function and Homeostasis

Brain iron is primarily stored as nonheme iron in ferritin, with only a small free pool involved in redox activity [56]. The human adult brain is estimated to contain nonheme Fe^3+^ content of approximately 1.63–10.22 µg/g wet weight [57]. As the most abundant trace metal in neurons, iron is integral to various cellular processes [58]. It is essential for normal brain development and function, playing a central role in neuronal maturation and differentiation. Mechanistically, iron deficiency impairs mitochondrial respiratory chain function and reduces ATP production, thereby disrupting axonal transport. The consequent reduction in mitochondrial volume leads to chronic energy deficits in neurons, ultimately affecting neuronal connectivity and synaptic function [59]. Furthermore, iron serves as an essential cofactor for key enzymes required for myelination, which is crucial for cognitive and behavioral functions [60]. Iron deficiency also activates cellular hypoxia signaling pathways, potentially compromising cerebrovascular integrity and resulting in neurovascular dysfunction [61]. Additionally, iron regulates neurochemical pathways, acting as a key cofactor for neurotransmitter synthetases [62]. As a result, iron deficiency impairs neurotransmitter synthesis, which can lead to abnormal brain development and neurological disorders [63].

Iron enters the brain primarily via two routes. The main one is the blood–brain barrier (BBB; Figure 1A). In this process, transferrin‐bound iron (TBI) binds to transferrin receptor 1 (TfR1) on the luminal side of brain microvascular endothelial cells (BMVECs) and is internalized via receptor‐mediated endocytosis. Within acidic endosomes, Fe^3^^+^ is dissociated from transferrin (Tf), reduced to Fe^2^^+^, and then transported into the cytoplasm by DMT1. Fe^2^^+^ may then be exported into the brain interstitial space through FPN on the BLM [64]. Iron can also traverse the BBB in the form of non‐Tf‐bound iron (NTBI) [54], released as complexes with small molecular ligands such as citrate, ATP, or ascorbic acid. Besides the BBB, the blood–cerebrospinal fluid barrier (BCB) serves as another critical interface regulating brain iron homeostasis (Figure 1A). The BCB is formed by tight junctions between choroid plexus epithelial cells [6, 60]. Iron traversal across this barrier relies on the coordinated action of multiple protein systems, including DMT1 and FPN, along with their ferroxidases Cp and HEPH. After transport into the CSF, iron binds to apo‐Tf for subsequent delivery [65]. The fenestrated capillaries of the choroid plexus facilitate rapid TfR1‐mediated endocytosis of TBI. Notably, the BCB also participates in brain iron clearance: the polarized distribution of DMT1 on the apical membrane may direct iron efflux from the CSF to the blood [66]. Together, these two barriers constitute a dual‐channel regulatory system maintaining dynamic iron homeostasis in the brain.

Astrocytes act as “traffic controllers” of iron metabolism within the brain (Figure 1B). Despite their strong iron‐uptake capacity, their intrinsic iron demand is relatively low, resulting in lower intracellular iron levels compared to neurons [67]. As the primary source of Cp in the brain [68], astrocytes oxidize Fe^2^^+^ exported via FPN to Fe^3^^+^ using both glycosylphosphatidylinositol (GPI)‐anchored Cp and soluble Cp [69]. The resulting Fe^3^^+^ binds to Tf in the interstitial fluid, enabling its distribution to other CNS cells [68]. This sophisticated regulatory system ensures a balanced iron supply, with studies showing that loss of Cp in astrocytes impairs iron export [70]. Astrocytes regulate iron metabolism through three key mechanisms: (1) secretion of hepcidin to downregulate FPN expression in BMVECs, thereby controlling iron efflux [71]; (2) efficient iron uptake via DMT1 localized in their end‐feet [67]; and (3) absorption of NTBI through the metal cation symporter ZIP14 (encoded by *SLC39A14*) and heme metabolism proteins HRG1 and HMOX1 [72].

Neurons can take up TBI via the Tf–TfR1 pathway and Fe^2^^+^ via the DMT1 pathway, respectively [62]. They may also absorb NTBI through lactoferrin‐mediated uptake, though this process carries a risk of iron overload [62]. Within cellular iron homeostasis, ferritin functions as the major storage depot, sequestering or releasing iron as needed, while FPN serves as the sole exporter of Fe^2^^+^ [73]. Furthermore, ferroxidases such as Cp and HEPH catalyze the oxidation of FPN‐exported Fe^2^^+^ to Fe^3^^+^, enabling its subsequent recycling via the Tf–TfR1 system [74].

Oligodendrocytes, which require substantial iron for myelin synthesis, are particularly vulnerable to iron deficiency and store large amounts of iron and ferritin [62, 75]. Unlike other neural cells, they lack TfR1 and primarily acquire iron via hepatitis A virus cellular receptor 1 (HAVCR1), a receptor for ferritin heavy chain (Fth) [76]. Intracellular ferritin‐stored Fe^3^^+^ is reduced to Fe^2^^+^ by reductases before its release, and iron efflux depends on the FPN/HEPH system [77].

Microglia have crucial roles in sustaining neural homeostasis and immune defense. Their iron transport dynamics are linked to their activation states: under proinflammatory conditions, they preferentially take up NTBI, whereas the upregulation of the TfR1/DMT1 pathway enhances self‐regulatory and anti‐inflammatory properties [62]. Iron export in microglia also relies on the FPN/HEPH system [62], with the IRE/IRP system fine‐tuning iron metabolism by modulating genes such as *TFRC*, which encodes TfR1, thereby ensuring a balanced intracellular iron environment [78].

#### 2.2.2 Copper in Neuronal Function and Homeostasis

The human brain contains an estimated copper content of 3.1–5.1 µg/g wet weight [79], constituting about 9% of the body's copper content, which is the third highest among all organs [80]. The distribution of copper in the brain is uneven, with higher copper levels in the ventricles and the substantia nigra (SN) [81]. Copper is integral to the function and homeostasis of the CNS, participating in various processes such as intracellular signaling, energy production, neuropeptide activation, catecholamine homeostasis, neuronal myelination, neurotransmitter synthesis, and synaptic transmission [81].

The levels and homeostasis of copper in the brain are regulated by the BBB and the BCB barriers [82] (Figure 1A). Approximately 70% of copper uptake into brain cells is mediated by CTR1 [83]. CTR1 not only transports copper into the cell but also assists in loading it onto copper‐dependent enzymes with the help of chaperonins. Excess copper from brain cells is released into the CSF via ATP7A. Subsequently, copper is taken up and stored by the cells forming the BCB, and can be re‐released into the CSF by ATP7B or transported into the bloodstream via ATP7A [79]. Both ATP7A and ATP7B are crucial for maintaining brain copper homeostasis from early developmental stages [84]. ATP7A is present in the BMVECs that constitute the BBB, and exhibits high expression in the choroid plexus, which is responsible for forming the BCB and modulating the levels of substances within the CSF [84].

Astrocytes play a crucial role in maintaining copper balance within the brain (Figure 1A). Positioned between the BBB endothelial cells and neurons, astrocytes are in close contact with both neuronal cytosol and capillary endothelial cells via their end‐feet [85]. They are the first to interact with metal ions that have traversed the BBB [86]. Astrocytes generally possess higher copper concentrations than neurons under both normal and disease states [87]. These cells can effectively absorb and retain copper, and due to their higher resistance to copper toxicity, they shield neurons from its harmful effects [86]. Astrocytes also contain high levels of metallothioneins (MTs) and GSH, which contribute to their enhanced capacity to cope with copper exposure [88]. The accumulation of copper in astrocytes may involve both CTR1‐mediated and CTR1‐independent transport processes [89], with ATP7A possibly playing a role in copper export [90]. Astrocytes can also prevent copper‐mediated inactivation or degradation of extracellular GSH by releasing antioxidants or copper chelators [91]. Given that alterations in brain GSH and energy metabolism are associated with NDDs, and that astrocytes can provide GSH precursors and energy substrates to neighboring neurons, dysfunctions in astrocytes may be associated with NDDs [92]. However, the specific mechanisms linking astrocytes to copper homeostasis and NDDs warrant further investigation.

### 2.3 Interaction of Iron and Copper With Key Transporters and Storage Proteins

#### 2.3.1 Iron Interaction With Key Transporters and Storage Proteins

In living organisms, iron ions are primarily transported or chaperoned by specific FeBPs rather than existing in a free form. Over 95% of plasma iron is carried by Tf, forming a reversible complex for systemic transport; the remainder exists as NTBI, primarily complexed with molecules like citrate and albumin [54]. The following sections detail the biochemical aspects of the interactions of iron with several key transporters and storage proteins: Tf, ferritin, and FPN (Table 1).

**TABLE 1:** Summary of the iron‐related proteins, key IDPs, iron level changes and the consequent pathogenic effects in NDDs.

NDD	Iron‐related proteins	Key IDPs	Iron level change in plasma/serum	Iron level change in CNS	Iron level change in CSF	Effect of iron dyshomeostasis	References
AD	Tf, FPN	APP, Aβ, tau	No specific data	↑	−	Induce the aggregation of Aβ and tau Aβ/tau‐Fe produces ROS	[93]
PD	Tf, FPN	αSyn	↓	↑	↑	Promote oligomerization of αSyn Accelerate aggregation of αSyn	[94]
PrD	−	PrP^sc^	No specific data	↑	↑	Promote the conversion of Fe^3^^+^ to the more bioavailable Fe^2^^+^	[95]
HD	FPN	mHTT	No change	↑	↑	Induce neuroinflammation Lead to lipid peroxidation	[96, 97]
ALS	−	−	↑	↑	↑	Induce ferroptosis and neuroinflammation Lead to oxidative stress	[98, 99, 100]
MS	−	−	↓	↑	No change	Induce ferroptosis and excitotoxicity Lead to oxidative stress	[101, 102]

##### 2.3.1.1 Iron Interaction With Tf

Tf is an abundant plasma glycoprotein that functions as a natural chelator and the principal systemic iron transporter. The primary role of Tf involves the reversible binding of Fe^3^^+^, which is essential for maintaining iron in a soluble state and protecting cells from the toxic free radicals generated by iron. Tf can bind either one iron atom (monoferric Tf) or two iron atoms (diferric Tf) and exhibits the strongest iron‐binding capacity at neutral pH, releasing iron when the pH drops below 6.5. Although Tf can bind approximately 30 different metals, its affinity for iron is the highest [103]. Notably, given the high expression of TfR1 on brain endothelial cells, Tf holds significant potential as a carrier for drug delivery, particularly for transporting therapeutic molecules across the BBB.

Tf consists of 679 amino acid residues with an apparent molecular weight of approximately 80 kDa [103]. It can be structurally divided into two nearly symmetrical domains: the N‐terminal domain consisting of 336 amino acids and the C‐terminal domain containing 343 amino acids. These domains are connected by a short peptide chain. Each domain exhibits a structure composed of several α‐helices overlaying a β‐sheet backbone, with their interaction forming a hydrophilic MBS. Within this site, iron is coordinated in a distorted octahedral geometry, binding to oxygen atoms from tyrosine and aspartate residues, a nitrogen atom from His, and two oxygen atoms from a carbonate anion [104]. During the process of iron binding and release, Tf undergoes conformational changes [104].

##### 2.3.1.2 Iron Interaction With Ferritin

Ferritin is the primary iron‐storage protein in various organisms, capable of storing iron in a nontoxic and bioavailable form [105]. It is a highly conserved iron‐storage protein across species and possesses the catalytic function of converting Fe^2^^+^ to Fe^3^^+^ for the purpose of storage. The human ferritin is structured as a spherical nanocage composed of 24 self‐assembling subunits [103]. In vertebrates, these subunits are categorized into two types: ferritin heavy chain (ferritin‐H, Fth), which exhibits ferroxidase activity, and ferritin light chain (ferritin‐L, Ftl), which facilitates iron nucleation. This assembly of these 24 subunits, known as maxi‐ferritin, forms an octahedral structure characterized by an internal cavity that is capable of accommodating up to 4500 iron atoms [106]. The iron‐free form of this protein, referred to as apoferritin, can undergo controlled disassembly and reassembly through the modulation of pH [107].

##### 2.3.1.3 Iron Interaction With FPN

FPN is recognized as the only known mammalian iron efflux transporter, facilitating the transport of Fe^2^^+^ ions. The mechanism of iron transport is dependent on dual MBS in its N‐ and C‐terminal domains, respectively. The N‐terminal domain contains the primary functional site for iron transport, where residues D39 and H43 in the TM1 helix are responsible for the direct coordination of Fe^2^^+^. In the C‐terminal domain, a tetrahedral coordination network formed by D325/C326 in TM7b and H507 in TM11 serves as a critical regulatory site, which not only binds Fe^2^^+^ but also interacts with the C‐terminus of hepcidin [108]. Notably, the presence of iron significantly enhances the binding affinity of FPN to hepcidin, suggesting a model that hepcidin mainly targets FPN molecules loaded with iron [108].

The iron transport process relies on an intracellular gating system, which includes ionic bonds such as R489–D157, to maintain an outward‐open conformation [108]. Furthermore, the nonhelical region of TM7b acts as a structural hinge that facilitates conformational changes, with this process being modulated by transmembrane pH gradients [108]. Mutations in these sites have distinct effects: alterations in the N‐terminal D39 directly impair iron transport functionality and can lead to hemochromatosis, mutations in the gating region, such as R88/E486, resulted in a locked conformation that blocks iron efflux, while mutations in the C‐terminal binding site (e.g., C326S/H507R) specifically disrupt this cooperativity without affecting basal transport activity. This intricate dual‐site design enables FPN to execute iron efflux while remaining sensitive to regulation by systemic iron status [108].

#### 2.3.2 Copper Interaction With Key Transporters and Storage Proteins

The two primary oxidation states of copper ions, Cu^+^ and Cu^2+^, have distinct coordination preferences; Cu^+^ ions preferentially bind to soft sulfur‐containing donors (e.g., cysteine and methionine), whereas Cu^2+^ ions are more likely to coordinate with intermediate or hard nitrogen‐containing ligands (e.g., His or oxygen donors such as glutamate or aspartate) [30]. This difference in binding preferences is crucial for understanding how copper interacts with different cellular components.

As mentioned above, cells encode an array of CuBPs for the uptake, transport, storage, and efflux of copper ions. In the following section, we describe in detail the biochemical aspects of the interaction of copper ions with CTR1, MTs, ATP7A, ATP7B, and Cp (Table 2).

**TABLE 2:** Summary of the copper‐related proteins, key IDPs, copper level changes and the consequent pathogenic effects in NDDs.

NDD	Copper‐related proteins	Key IDPs	Copper level change in plasma/serum	Copper level change in CNS	Copper level change in CSF	Effect of copper dyshomeostasis	References
AD	−	APP, Aβ, tau	↑	↓	No change	Induce the aggregation of Aβ and tau Aβ/tau‐Cu produces ROS	[109, 110]
PD	−	αSyn	↑	↓	No change	Induce the aggregation of αSyn Reduce dopamine metabolism Induction of oxidative stress	[111, 112, 113]
PrD	−	PrP^sc^	↑	↓	No specific data	Promote the conformational transition of PrP^C^ to PrP^SC^ Induce the aggregation of PrP^C^ Increase protease resistance and protein infectivity	[114]
HD	−	mHTT	No change	↑	↑	Induce the aggregation of polyQ tract and HTT fibers Reduce LDH activity	[115]
WD	ATP7B	−	↓	↑	↑	Copper overload in the brain Dysfunctional astrocytes	[116, 117]
MD	ATP7A	−	Total copper↓ Free copper ↑	↓	↓	Copper deficiency in the brain Reduce activity of specific copper‐dependent enzymes	[118, 119]
ALS	SOD1	−	↓	↑	↑	Mutations and misfolding of SOD1, leading to oxidative stress	[120, 121, 122]
MS	−	−	↑	↑	↑	Dysregulation of copper transport, causing demyelination	[123, 124]

##### 2.3.2.1 Copper Interaction With CTR1

The evolutionarily conserved, high‐affinity active copper uptake transporter CTR1 protein, located on the plasma membrane, is instrumental in copper transportation in intestinal cells [125]. In the CNS, CTR1 is notably abundant in brain capillary endothelial cells that form the BBB, where it is recognized as the primary conduit for copper's passage from the bloodstream into the brain [126]. CTR1 is also present on the apical cell surface of the choroid plexus of the BCB, facilitating the export of copper from the brain into the CSF [45] and within the brain's parenchyma [112]. Notably, CTR1 is observed surrounding neuromelanin granules in the SN, where it likely modulates the pigment's copper uptake [127].

CTR1 is capable of transporting copper into the cell in a low‐copper environment, accounting for at least 80% of cellular copper uptake [128]. CTR1 exists in the cytoplasmic membrane as a monomeric protein molecule assembled into a homotrimeric channel and selectively transports Cu^+^ [129]. CTR1 can switch its position between the plasma membrane and intracellular vesicles [130]. When extracellular copper concentrations are high, CTR1 is rapidly internalized and degraded [131]. Other factors affecting CTR1 localization are low extracellular pH and tissue hypoxia, while increased GSH levels mediate CTR1 degradation [132].

The human CTR1 protein consists of 190 amino acids, featuring an extracellular N‐terminal domain of approximately 67 amino acids and a cytosolic tail of around 15 amino acids. The ectodomain is rich in methionine and His, both capable of coordinating metal ions [133]. The ectodomain of the CTR1 protein has two glycosylation sites: A site for N‐glycosylation at position 15 on Asn15 and a site for O‐glycosylation at position 27 on Thr27. Research on mutations has demonstrated that O‐linked glycosylation shields the extracellular domain of CTR1 from proteolytic degradation, generating a truncated CTR1 protein with approximately twofold diminished copper transport activity [134]. The ectodomain of CTR1 is known to bind three Cu^+^ via methionine‐rich sequences and an additional two Cu^2+^ through the action of the N‐terminal copper/nickel (His‐Met‐Asp clusters) [135]. Current understanding suggests that the His‐Cys‐His motif located at the tail end of each CTR1 monomer acts as a binding site for Cu^+^. This binding occurs before the copper ions are handed off to GSH, MTs, or other copper‐binding molecules for further utilization [136].

##### 2.3.2.2 Copper Interaction With MTs

MTs, a group of cysteine‐rich metalloproteins and peptides, are distinguished by their unique three‐dimensional structures that feature either single or multiple clusters of metal‐thiolate bonds [137]. This unique feature endows them with the capacity for metal binding [138]. MT‐1 and MT‐2 can be induced and found throughout the body in various organs. MT‐3 and MT‐4 are predominantly expressed in the CNS and stratified epithelia, respectively, and their expressions are not typically induced by the same stimuli as MT‐1 and MT‐2 [139]. The strong affinity of MTs for copper, coupled with the known release of copper at glutamatergic synapses, implies that these proteins may act as a buffer for copper ions in the synaptic region [140]. In the majority of instances, MTs serve a neuroprotective function [141]. The molecular connection between neuroinflammation and biometals is established through the regulation of MTs [142].

MTs are known for their intrinsic functions, which stem from their ability to bind to transition metals tightly. Their key biological functions include maintaining zinc and copper homeostasis, as well as safeguarding against environmental metal toxins and oxidative stress. Moreover, MTs have been shown to play specialized roles in stress adaptation, shielding the brain from injury, modulating neuronal development, exerting antiapoptotic effects, and interacting with and neutralizing metal‐based chemotherapy drugs, which can lead to drug resistance [140].

Mammalian MTs are composed of a single polypeptide chain containing 61–68 amino acids, with a consistent pattern of 20 cysteine residues. These cysteines are grouped in motifs such as Cys‐X‐Cys, Cys‐Cys, and Cys‐X‐X‐Cys. MT‐1 and MT‐2 are the primary zinc‐binding proteins within cells and are upregulated in the presence of heavy metals. MT‐3 and MT‐4 are characterized by their mixed content of Zn^2+^ and Cu^+^ [140]. Mammalian MTs feature a dumbbell‐shaped conformation, consisting of two separate domains linked by a flexible hinge. Each domain harbors its own metal‐thiolate cluster. The N‐terminal domain features an M(II)_3_Cys_9_ cluster, while the C‐terminal one has an M(II)_4_Cys_11_ cluster, with all the 20 conserved cysteines participating in metal binding [137]. In these clusters, the metals exhibit a tetrahedral coordination geometry, with both bridging and terminal cysteine thiolates. When loaded with Cu^+^ ion, MT can bind up to 12 Cu^+^, which are organized into two separate Cu(I)_6_‐thiolate clusters. In a dihedral and trigonal fashion, the Cu^+^ is coordinated by two to three sulfur atoms from both bridging and terminal cysteine ligands [137].

##### 2.3.2.3 Copper Interaction With ATP7A and ATP7B

ATP7A and ATP7B proteins are two human copper‐transporting ATPases involved in copper transport [143]. Although ATP7A and ATP7B share a high degree of sequence and structural similarity, their distribution within tissues, their specific functions, and how they are regulated vary considerably [144]. ATP7A is particularly important for transferring copper from the intestinal cells into the bloodstream by moving it across the BLM [145]. In contrast, ATP7B primarily functions in exporting extra copper to maintain intracellular copper balance. Under normal cellular copper levels, ATP7A and ATP7B localize to the trans‐Golgi network (TGN) to supply copper to newly synthesized cuproproteins. In response to high copper, these transporters relocate to the plasma membrane for copper export. ATP7B is targeted to the apical membrane, while ATP7A moves to the BLM of polarized cells [146].

**FIGURE 2:**
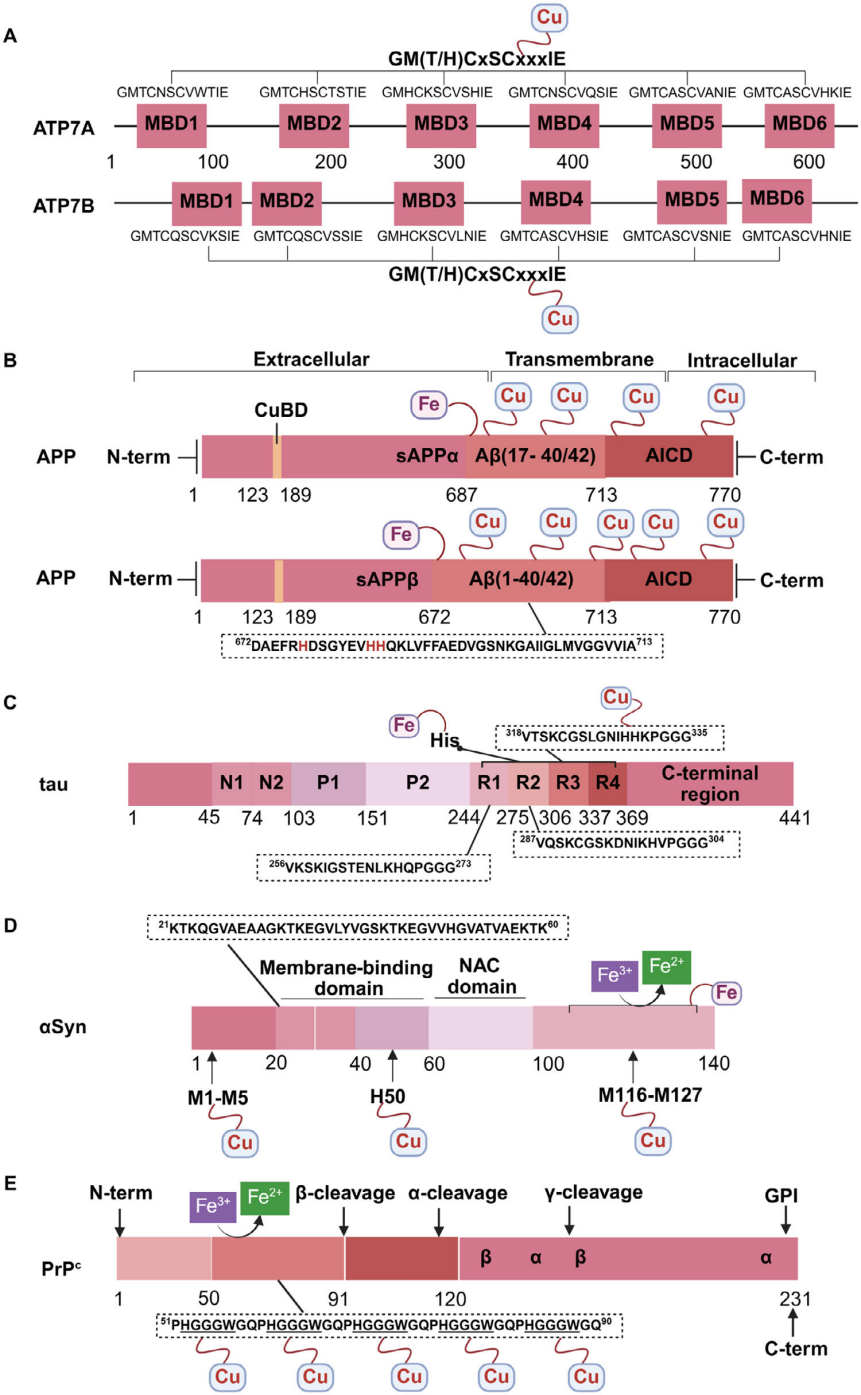
Schematic representation of the iron‐ and copper‐binding motifs within several critical proteins involved in NDDs. (A) Scheme of the domain organization of the ATP7A/B proteins and the sequence of the six MBD structural domains that bind copper ions. (B) Amino acid coordination of iron and copper ions in the binding sites of APP. (C) Scheme of the domain organization of tau protein and the sequence of the R1–R3 domains that bind iron and copper ions. (D) Scheme of the domain organization of αSyn protein and the sequence of its membrane‐binding domain and NAC domain. The regions spanning amino acid 100–140 of αSyn can reduce Fe^3^^+^ to Fe^2^^+^. (E) Scheme of the domain organization of PrP^c^ protein and the sequence of the octarepeat domain that binds copper ions, with the specific residues HGGGW underlined. The region spanning amino acid 51–90 of PrP^c^ can reduce Fe^3^^+^ to Fe^2^^+^.

A unique characteristic of copper ATPases is six metal‐binding domains (MBDs) within the N‐terminal sequence, situated in the cytoplasmic region [147] (Figure 2A). Each MBD has a conserved ferredoxin‐like structure comprising roughly 70 amino acids, including a conserved CxxC motif that is crucial for Cu^+^ binding [148].

Inside the cell, the MBDs of ATP7A and ATP7B acquire copper ions from the copper chaperone ATOX1 [149], although these domains can also bind copper ions directly in vitro. Copper ions are complexed by both the MBDs and ATOX1 when they are in the oxidized form [150]. The lengths of the interdomain linkers within ATP7A and ATP7B exhibit specific arrangements. For both proteins, the membrane‐proximal MBD5 and MBD6 are linked by a brief connector. ATP7B and ATP7A exhibit distinct arrangements of their MBDs. In ATP7B, the first two MBDs are closely packed, with the longest linker located between MBD4 and MBD5. Conversely, in ATP7A, the longest linker lies between MBD1 and MBD2. Beyond the core copper‐binding CxxC motif, MBD sequences are highly conserved and share an extended signature motif, GM(T/H)CxSCxxxIE. This suggests that additional conserved residues are crucial for both copper transfer and overall domain stability. The copper‐binding sites within MBD4–6 of ATP7B and MBD5–6 of ATP7A are particularly similar, sharing the consensus sequence of GMTCASCVxxIE. Across all MBDs, MBD3 shows the highest level of sequence similarity between ATP7B and ATP7A. Moreover, MBD3 is unique among both proteins for having a lysine residue immediately after the first cysteine of the CxxC motif. A conserved threonine residue preceding the copper‐binding motif in most MBDs (except MBD3) promotes directional copper transfer from ATOX1 by destabilizing the ATOX1‐Cu–MBD intermediate complex [151]. This highlights the complex and specific roles of ATP7A and ATP7B in copper homeostasis, as well as the intricate mechanisms by which they operate, including their structure and function.

##### 2.3.2.4 Copper Interaction With Cp

The human Cp protein is a glycoprotein primarily synthesized in the liver [152]. It is released into the bloodstream with copper ions already bound during its early synthesis phase, along with the attachment of a polysaccharide chain [112]. Cp synthesized in the brain is mainly in the GPI‐anchored form [153]. This multifunctional enzyme serves various roles, including functioning as a copper transporter, iron oxidase, amine oxidase, nitric oxide oxidase, glutathione peroxidase, and an anti‐inflammatory agent [154].

The copper chaperone ATOX1 delivers copper to Cp synthesized by liver and kidney cells [155]. Cp protein is the predominant transporter of copper in the serum. Upon arrival at the surface of target cells, Cp engages with its specific receptor to relinquish copper, which is subsequently internalized and utilized by these cells. The interaction and release of copper ions by Cp facilitate the distribution of copper across the body. Furthermore, copper ions also modulate the synthesis and metabolism of Cp [153]. Cp binds to Cu^2+^ in blood and tissue fluids, significantly inhibiting Cu^2+^‐induced lipid peroxidation and erythrocyte hemolysis [156].

Structurally, Cp protein is composed of about 50% β‐sheets and random coils, with almost no presence of α‐helical structures [157]. Cp has six compact structural domains capable of binding six copper ions. A trinuclear multicopper cluster, located at the interface of the first and sixth domains, plays a critical role in maintaining both the catalytic activity and structural stability of Cp [157]. The other three copper ions exist in monocopper form in the second, fourth, and sixth structural domains, respectively. As a member of the multicopper oxidase family, Cp accepts electrons from substrates in its monocopper ion center and transfers them to the multicopper ion center to reduce oxygen to water [158]. In this process, copper ions undergo oxidation, facilitating their transport and metabolism within the body [159].

### 2.4 Interaction of Iron and Copper With Key IDPs in NDDs

Among the various FeBPs and CuBPs, the study of iron and copper interactions with IDPs has attracted considerable interest due to their close relationship with the development of NDDs. IDPs, unlike folded proteins, possess a high degree of flexibility, allowing them to readily accommodate the conformational demands of various ligands. The binding sites for iron and copper ions in IDPs have different geometries and utilize different amino acid residues, reflecting the versatility of these proteins in coordinating with different metal ions. The complexes formed between iron and copper ions and IDPs are capable of participating in redox reactions. In the next section, we elaborate on the biochemical aspects of the iron and copper interaction with several key IDPs, including Aβ, tau, PrP, αSyn, and mHTT, that are known to be involved in NDDs (Figure 2B–E).

#### 2.4.1 Iron and Copper Interaction With APP and Aβ

Aβ peptide, first identified as a major component of meningeal vascular amyloid in 1984 [160], was soon established as a primary constituent of amyloid plaques [161]. In 1992, Hardy and Higgins first proposed the amyloid cascade hypothesis, postulating that the Aβ deposition in the brain marked the inception of the pathogenesis of AD [162]. Since its inception, this hypothesis has emerged as one of the foremost models in explaining the pathogenesis of AD [163, 164]. Aβ is derived through a sequential proteolytic process of β‐amyloid precursor protein (APP) mediated by β‐secretase (BACE1) and γ‐secretase.

APP is a type I membrane protein that is particularly abundant in neuronal tissues [165]. The functions of APP are diverse, linking to various processes such as neurogenesis, neuronal differentiation, synaptic function, cell cycle regulation, cell adhesion, and calcium (Ca^2+^) homeostasis [166]. Mice lacking APP tend to have a shortened lifespan, cognitive and learning difficulties, and altered metal balance in brain regions [167]. Cleavage of APP can proceed via two primary pathways: the amyloidogenic pathway, which leads to the formation of amyloid plaques, and the more prevalent nonamyloidogenic pathway. The nonamyloidogenic pathway is initiated by α‐secretase, which releases the extracellular soluble domain of APP, known as sAPPα, leaving behind a membrane‐attached C83 fragment [168]. The action of α‐secretase within the Aβ region prevents the formation of Aβ peptides. In addition, sAPPα can reduce Aβ production by inhibiting BACE1, an enzyme involved in the amyloidogenic pathway. The amyloidogenic pathway begins with the action of BACE1, which is responsible for the initial cleavage [169]. This process generates the extracellular fragment known as sAPPβ and the membrane‐attached C99 domain [170]. Cleavage of C83 or C99 by γ‐secretase leads to the production of the amyloid precursor protein intracellular domain (AICD), which acts as a transcription factor regulating the processing and intracellular trafficking of APP [171].

Aβ, a peptide of 37–49 amino acids, is a critical end product of the amyloidogenic pathway in APP processing. Aβ_40_ and Aβ_42_ are the two main forms, with Aβ_40_ constituting roughly 90% of the overall Aβ peptides produced, while Aβ_42_ makes up approximately 5%–10%. Aβ_42_ tends to aggregate more readily than Aβ_40_ because of its heightened hydrophobicity at the C‐terminus [172]. Typically, the levels of Aβ in the human brain and CSF fall within the picomolar range [173]. Both excessively high and low levels of Aβ can adversely affect synaptic function. Research has indicated that low levels of Aβ in the picomolar range can improve long‐term potentiation [174], increase dendritic spine density, and facilitate vesicle docking [175]. While there is some debate about the exact concentrations of Aβ required for different synaptic functions, the evidence suggests a “hormetic effect” for Aβ. This means that there is a beneficial effect within an optimal dosage range, but detrimental effects occur if the levels are either too high or too low [176].

##### 2.4.1.1 Iron Interaction With APP and Aβ

Iron interacts with APP and Aβ at different sites, which could result in the disturbance of iron homeostasis, oxidative stress, and the development of NDDs.

APP plays a role in regulating iron export by stabilizing FPN on the cell surface, thereby facilitating the efflux of Fe^2^^+^ [177]. A proposed Fe^2+^‐binding site in the E2 domain (comprising residues approximately 401–417 in the APP‐770 isoform) contains a conserved REXXE motif (Figure 2B), similar to the ferroxidase center of ferritin, which was initially thought to possess ferroxidase activity that oxidizes Fe^2^^+^ to Fe^3^^+^ [177]. Nonetheless, subsequent studies have questioned the intrinsic ferroxidase catalytic activity of the E2 domain or related peptides, suggesting that APP primarily functions to stabilize FPN without directly participating in the oxidation of iron [178].

Aβ, especially the Aβ_40_ and Aβ_42_ variant, demonstrates a high affinity for binding both Fe^2^^+^ and Fe^3^^+^ within its N‐terminal region (residues 1–16; Figure 2B), thereby promoting redox cycling, generating reactive oxygen species (ROS) via the Fenton reaction, and leading to the formation of toxic oligomers and ordered fibrils [179]. The primary coordination involves three His residues (His6, His13, and His14), with potential participation from Tyr10, Asp1, or Glu11 [180]. Nuclear magnetic resonance (NMR) studies confirm that Fe^2^^+^ binds to Aβ_16_/Aβ_40_ at physiological pH, with His residues as key ligands [181].

##### 2.4.1.2 Copper Interaction With APP and Aβ

APP has two structural copper‐binding domains (CuBDs) [182]. In particular, the extracellular E2 structural domain of APP contains two typical copper‐binding sites (Figure 2B). One site is coordinated by a 4‐His sphere, reminiscent of the coordination in SOD1, while the other site has a tripartite geometry involving a His, the carboxyl group of a glutamate residue, and a solvent water molecule, with a nearby methionine potentially also participating in coordination [183]. In addition, APP contains a CuBD in which copper binds in a twisted tridentate bipyramidal geometry to two His residues, one tyrosine, and two solvent molecules [184].

The interaction of Cu with Aβ is typically inaccessible unless APP is processed by β‐ and γ‐secretases. Once released from APP, Aβ exhibits a strong affinity for copper in vitro, and this interaction influences the kinetics and trajectory of amyloid fibril and plaque formation [185]. Aβ binds to Cu^+^ in a dihedral geometry through two His residues, whereas it adopts a twisted square‐based pyramidal geometry when binding to Cu^2+^ [186]. The binding of copper ions to Aβ catalyzes the formation of ROS, which occurs via a complex transition state known as the “in‐between state” [186].

#### 2.4.2 Iron and Copper Interaction With Tau

The human tau proteins belong to the family of microtubule‐associated proteins (MAPs) [187]. Tau is a classic example of a “natively unfolded” protein. Its low secondary structure content is responsible for its notable resistance to heat and acid [188]. Since unfolded proteins tend to be highly flexible in conformation, structural analysis of full‐length tau by crystallography has not been possible [189]. Tau proteins are classified into six different isoforms based on the number of microtubule‐binding repeats (MTBRs, named R1–R4 in sequence) within the microtubule‐binding domain (MTBD) in their C‐terminus and the presence or absence of inserts in their N‐terminus. Specifically, tau protein isoforms are categorized into two main groups based on the number of MTBRs: three isoforms contain three repeats (3R), and three contain four repeats (4R), resulting in lengths ranging from 352 to 441 amino acids. This difference is determined by the alternative splicing of exon 10: its inclusion produces 4R isoforms (0N4R, 1N4R, 2N4R), while its exclusion yields 3R isoforms (0N3R, 1N3R, 2N3R). These six isoforms are also known as τ3L, τ3S, τ3, τ4L, τ4S, and τ4 [190].

Tau isoforms show distinct expression patterns throughout brain development. In the adult human brain, a balanced ratio of 3R to 4R tau is maintained. Notably, 3R tau is predominantly expressed during developmental stages, whereas the 4R tau is produced in adulthood [191]. Compared to 3R tau, 4R tau exhibits greater activity in promoting microtubule assembly [192]. An imbalance in the ratio of 3R to 4R has been implicated in attention deficit disorder and other tauopathies [193].

Each MTBR consists of a conserved sequence of 18 amino acids interspersed with spacer regions containing either 13 or 14 amino acids [194]. Studies have shown that tau can accelerate the formation of microtubules in vitro and also reduce their disassembly rate [195]. The 18‐amino acid motifs within tau connect to microtubules through a series of weak, flexible binding sites distributed along the protein [196]. In particular, the most effective segment for stimulating microtubule assembly is the region between R1 and R2, specifically the peptide ^275^VQIINK^280^ [197].

Microtubules are critical for cell shape determination and cell division [198]. They also act as pathways for the transport of cellular components by motor proteins that move along the microtubules [199]. These motor proteins are responsible for moving organelles such as mitochondria, lysosomes, peroxisomes, and vesicles involved in endocytosis or exocytosis, either to the edge of the cell or back to the microtubule‐organizing center [200]. Research has shown that tau can influence the process of axonal transport. It has been suggested that tau's strong interaction with microtubules may disrupt the normal transport of cellular cargo, possibly by dissociating it from kinesin motor proteins. However, tau does not appear to affect the rate at which kinesin transports its cargo [201], and its knockdown or inhibition in mouse models and primary neurons does not impair microtubule assembly or axonal transport [202]. This suggests that tau's normal functions may be compensated for by other MAPs [203]. Similarly, humans carrying mutations or complete disruptions of *tau* genes develop normally [204]. Thus, the neuron‐specific function of tau remains to be clarified.

##### 2.4.2.1 Iron Interaction With Tau

The interaction between iron and tau protein is highly dependent on the protein's native conformation and specific residues, particularly His. It is proposed that this interaction occurs via a putative iron‐binding motif located within tau's MTBD [205, 206]. Iron exhibits a strong affinity to His residues [180] (Figure 2C). Modification of the His residues in tau effectively prevents iron binding, underscoring their critical role [205].

The two redox states of iron, Fe^2^^+^ and Fe^3^^+^, exert distinct and contrasting effects on tau structure and function. Fe^2^^+^ acts rapidly to induce reversible conformational changes from random coil to α‐helix, promotes tau dimerization and increases membrane thickness, which indicates a key role in the initiation of aggregation [207]. This binding may also be mediated by threonine and tyrosine residues, as tyrosine phosphorylation significantly weakens the interaction with Fe^2^^+^. In contrast, the interaction with Fe^3^^+^ is a slower and largely irreversible process. Although it also induces conformational changes, these effects are less pronounced compared to Fe^2^^+^. Fe^3^^+^ also inhibits or diminishes tau–tau interactions without significantly altering membrane thickness [207]. Moreover, the binding mechanisms for the two ions differ, as phosphorylation on serine/threonine has a weaker impact on Fe^3^^+^ binding compared to Fe^2^^+^.

Furthermore, the interaction between iron and tau is influenced by other metal ions. Fe^3^^+^ completely inhibits the uptake of Cu^2^^+^ by tau, possibly by occupying the same binding sites or inducing a conformational change that blocks binding. Fe^2^^+^ has only a minor effect on the interaction between Cu^2^^+^ and tau, but competes with Cu^2^^+^ for binding after serine/threonine phosphorylation [207].

##### 2.4.2.2 Copper Interaction With Tau

Copper can interact with the microtubule‐bound tau proteins and affect their secondary structures [208]. However, the residues involved in the interaction between microtubule and tau do not appear to be involved in copper binding [209]. Copper binds directly to tau‐derived peptides within the individual R1–R4 repeats or their smaller fragments, suggesting that copper binding to tau induces conformational changes in the MTBD [210].

A particularly strong binding to copper is observed with the octadecapeptide ^318^VTSKCGSLGNIHHKPGGG^335^ within the R3 repeat sequence, which is known to play an important role in tau aggregation [209] (Figure 2C). The imidazole rings of the two His residues within this 18‐amino acid peptide are essential for the coordination of Cu^2+^. This coordination takes place in a square planar arrangement, involving the two imidazole rings, the carboxyl group at the C‐terminus, and the amino group at the N‐terminus of the His residues. The conformation of this complex is highly pH‐dependent, with a mixture of random structures, α‐helices, and β‐turns predominating at acidic pH, and an increase in α‐helices at physiological pH [209]. The octadecapeptide ^287^VQSKCGSKDNIKHVPGGG^304^ from the R2 repeat binds copper with relatively low affinity at a stoichiometric ratio of 1:1. Under physiological pH conditions, the imidazole groups of cysteine and His are crucial for the coordination of copper, irrespective of the peptide backbone. Cu^2+^ facilitates the formation of α‐helices within the R2 region, with an increase in α‐helices forming with increasing copper concentration. R2 peptides have a greater propensity to form α‐helices than R3 peptides [209]. Similarly, the peptide ^256^VKSKIGSTENLKHQPGGG^273^, derived from the R1 repeat, binds copper in a 1:1 ratio. In contrast, Cu^2+^ induces a smaller extent of structural changes in the Cu‐R4 complex [209].

It is hypothesized that the aggregation stimulated by copper in different structural domains may depend on the number of His residues present in the sequence, with a higher number of His residues predisposing to aggregation. Notably, the interaction of R2 and R3 repeat sequences with Cu^2+^ leads to ROS production. The redox activity of Cu^2+^ facilitates the formation of disulfide bonds between the thiol groups of cysteine residues and generates Cu^+^, which can further catalyze ROS production through a Fenton‐type reaction. In addition, the Cu^2+^‐2His complex can bind to cysteine and form the Cu^+^‐2His complex during cysteine oxidation, promoting the Fenton reaction and increasing ROS production. This cycle can regenerate the Cu^2+^‐2His complex, allowing it to oxidize cysteine again. Neither oligomerization nor ROS production was observed for the R1 and R4 repeat sequences [211].

#### 2.4.3 Iron and Copper Interaction With αSyn

αSyn is a compact, water‐soluble protein that is widely distributed throughout the body. αSyn is predominantly found at the presynaptic terminals of dopaminergic neurons, where it cooperates with SNARE (soluble N‐ethylmaleimide‐sensitive factor attachment protein receptor) proteins to regulate dopamine release, synaptic function, and plasticity [212]. αSyn is in a dynamic equilibrium, fluctuating between a soluble and a membrane‐bound state, with its secondary structure varying according to its state [213].

Human αSyn protein consists of 140 amino acids and possesses a distinctive 11‐amino acid motif (with a consensus of XKTKEGVXXXX) repeated seven times throughout the sequence [214]. Within this repetitive sequence is the NAC (nonamyloid component) region, which is remarkably hydrophobic and tends to aggregate in human αSyn [215]. Upon binding to lipid membranes, the seven 11‐residue motifs within αSyn undergo a transition into an α‐helical configuration, a structural feature similar to that of apolipoproteins [216]. αSyn can adopt either a single, extended α‐helix associated with large diameters (approximately 100 nm) and small curvature, or a disrupted α‐helix associated with small, highly curved vesicles, depending on the curvature of the membrane [217]. Strikingly, αSyn can also adopt a β‐sheet amyloid structure under pathological conditions. This β‐sheet structure is correlated with the aggregation of αSyn, the formation of fibrils, and the subsequent accumulation within Lewy bodies. The β‐sheet structure is believed to be neurotoxic; however, the exact form of the neurotoxic species has not been definitively identified [213].

##### 2.4.3.1 Iron Interaction With αSyn

Iron can directly bind to αSyn, which induces a conformational change and promotes its aggregation, thereby playing a role in PD pathogenesis. The primary interaction site is the C‐terminal region (residues 96–140), which is characterized by a high percentage of negatively charged acidic amino acids, specifically aspartate and glutamate (Figure 2D). At physiological pH, the αSyn protein carries a net negative charge of approximately 24, with 15 of these charges concentrated at the C‐terminus [218]. The binding mechanism is primarily driven by nonspecific electrostatic interactions, where the positively charged Fe^2^^+^ and Fe^3^^+^ ions are attracted to these negatively charged carboxylate groups. This is consistent with the measured binding affinity of approximately 173 µM, which is classified as a moderate or relatively low affinity, suggesting the lack of a specific and clearly defined structural binding site. The polyvalent nature of Fe^3^^+^ is particularly relevant, as it can engage with two or more negatively charged carboxylate groups simultaneously. This property allows Fe^3^^+^ to act as a molecular “bridge,” forming cross‐links within a single αSyn molecule or between different molecules. This bridging effect not only neutralizes charge but also induces conformational changes, thus promoting oligomerization and significantly accelerating the aggregation process of pathological αSyn [94].

##### 2.4.3.2 Copper Interaction With αSyn

The C‐terminus of αSyn is unstructured, characterized by its acidic nature and high glutamate content. This region has been implicated in a variety of interactions with ions, polycations, and polyamines (Figure 2D); it also modulates the membrane binding and aggregation tendency of αSyn [213]. Besides the C‐terminal binding site, another distinct copper binding site with nanomolar affinity has been recognized within the N‐terminal domain [219]. Empirical data indicate that when Cu^+^ is present, the protein features two binding sites, one at the N‐terminus and one at the C‐terminus, each showing similar affinities within the micromolar range. Notably, the first copper‐binding site is composed of the sulfur atoms from the thioether groups of Met1 and Met5, whereas the second site involves the thioether groups of Met116 and Met127 [220], with the former region exhibiting a stronger affinity for copper binding [221]. In addition, a recent report showed that under physiological conditions, at a pH of 7, there are three copper binding sites on αSyn, which are associated with the amino acid residues Met1‐Asp2, His50, and Asp121 [222]. Studies have suggested that copper binding at His50 of αSyn may also play an important role in the pathogenesis of PD in humans [223]. However, at a lower pH of 5, the binding at His50 is significantly reduced, and αSyn has an alternative copper binding site located between Asp119 and Glu123 [224].

#### 2.4.4 Iron and Copper Interaction With Different PrP Conformations

The major PrP (encoded by *PRNP*) is a highly conserved, ubiquitously expressed glycoprotein [225]. It exists in the normal cellular form PrP^C^, and the pathogenic form called “prion protein scrapie” (PrP^Sc^) [226]. The main difference between them lies in their structural conformation. PrP^C^ is predominantly rich in α‐helices, whereas PrP^Sc^ is characterized by the presence of multiple β‐sheets, low solubility, and resistance to proteases [227]. PrP^C^ is a GPI‐anchored plasma membrane protein, predominantly found on the neuronal cell surfaces, with about 10%–20% in the cytosol [228]. PrP^C^ is implicated in myelin homeostasis, neuroprotection, and metal ion homeostasis [229]. In neurons, PrP^C^ is found in the pre‐ and postsynaptic regions of axon terminals and is involved in axonal transport [230].

Mature human PrP^C^ consists of an unstructured N‐terminal region (amino acid 23–120) and a structured C‐terminal region (amino acid 121–231) [231]. The N‐terminus contains an octarepeat region consisting of four PHGGGWGQ sequences with 5 Cu^2+^ binding sites [232] in tandem at amino acid 51–90, the C‐terminal region is made up of three alpha‐helical segments, two beta‐sheet structures, a disulfide linkage between cysteines at positions 179 and 214, and a pair of N‐linked glycosylation sites at the sequence spanning amino acids 181–197 [233].

PrP^C^ can be converted to the β‐sheet‐rich isomer PrP^Sc^, which is inclined to self‐catalyzed conversion and accumulation into nonsoluble complexes [234]. Abnormal accumulation of pathological proteins in the brain can lead to transmissible spongiform encephalopathies, also known as PrD. However, the mechanism by which PrP^C^ is converted to PrP^Sc^ and PrD remains unknown [229]. PrP^C^ can undergo four different post‐translational modifications (PTMs) by cleavage, resulting in the generation of PrP fragments. Alpha‐cleavage and beta‐cleavage occur in the disordered N‐terminal region, while gamma‐cleavage and PrP shedding happen in the structured C‐terminal region. In addition to these cleavage events, PrP^C^ has also been observed to be cleaved under laboratory conditions by phospholipase C, targeting the GPI‐anchoring site [235].

##### 2.4.4.1 Iron Interaction With Different PrP Conformations

Accumulating evidence indicates that the PrP^C^ protein is involved in brain iron metabolism. This is supported by the observation that *PRNP* gene knockout mice exhibit disrupted brain iron metabolism and reduced iron levels [236]. In addition, abnormal levels of ferroxidase and Tf have been observed in the CSF of patients with Creutzfeldt–Jakob disease [237].

Mechanistically, PrP^C^ functions as a ferrireductase, utilizing its octapeptide repeat domain to promote the conversion of Fe^3^^+^ to the more bioavailable Fe^2^^+^ (Figure 2E). This reduction facilitates cellular iron uptake by enabling the transport of Fe^2^^+^ across the membrane via the ZIP14/DMT1 complex [95]. Notably, the mRNA of the *PRNP* gene itself contains an IRE, making its expression subject to regulation by IRPs in response to changes in cellular iron [238]. This forms a complex feedback regulatory loop influencing brain iron homeostasis, wherein PrP^C^ modulates iron availability, while its own synthesis is, in turn, influenced by iron levels.

##### 2.4.4.2 Copper Interaction With Different PrP Conformations

Recent studies have shown that the native PrP^C^ protein can adopt a copper‐binding form in vivo, with a preference for Cu^2+^ ions [239]. PrP^C^ does not exhibit strong interactions with other divalent cations, including Ca^2+^, Co^2+^, Mg^2+^, Mn^2+^, and Ni^2+^. The primary Cu^2+^ binding site is located in the N‐terminal region of PrP, where four His residues, specifically H61, H69, H77, and H85 (according to the human protein residue numbering), within the octarepeat region, together with two adjacent His residues, H96 and H111, are responsible for the coordination of copper ions at a neutral pH [240] (Figure 2E). Crystallographic analysis of the PrP^C^‐Cu^2+^ complex reveals that Cu^2+^ is coordinated in an equatorial plane by the imidazole ring of the His, two neutralized glycine amide groups, and a carbonyl group of glycine, with an axial water molecule linked to the indole group of tryptophan [241]. The linkage between glycine and copper is unstable at pH below 6.5, suggesting a pH‐sensitive mechanism by which PrP can bind Cu^2+^ in the extracellular space or release copper within the endosome [241]. Recent research indicates that the binding of a single copper ion can confer PrP^C^ with protease resistance and detergent insolubility quickly and reversibly [242]. This finding underscores the important role of copper binding in the transition of PrP^C^ to a form with properties similar to the pathogenic PrP^Sc^.

Recently, two C‐terminal His residues, H140 and H177, have been shown to bind Cu^2+^ cooperatively, linking the N‐ and C‐terminal regions of PrP and enhancing their mutual interaction [243]. Moreover, H187 has also been identified as being involved in the coordination of Cu^2+^ [244]. The octarepeat region of PrP, involved in Cu^2+^ coordination, has a unique and uniform distribution of aromatic amino acids [245]. It is also proposed to form low‐complexity amyloid‐like kinked segments (LARKS) that are mainly composed of transient β‐sheet interactions [246]. These segments are thought to be involved in the liquid–liquid phase separation of low‐complexity domains within proteins that form biomolecular condensates, such as membraneless organelles [247]. Recent research has also shown that Cu^2+^ facilitates the aggregation of recombinant PrP in vitro and the aggregation of membrane‐anchored PrP^C^ within cells. Overexpression of PrP^C^ was found to attenuate Cu^2+^‐induced cytotoxicity, suggesting that PrP^C^ aggregation may serve as a copper buffering mechanism by scavenging Cu^2+^. PrP aggregation may be a key mechanism for various functions related to copper and other metal ions, including metal ion sensing, uptake, transport, and storage. These processes are critical for signal transduction and antioxidant defense. However, the production of ROS due to prolonged exposure to Cu^2+^ or H_2_O_2_ can trigger a transition from liquid to solid in PrP:Cu^2+^ condensates, potentially leading to the aggregation of pathological PrP [247].

#### 2.4.5 Iron and Copper Interaction With mHTT

The secondary structure of Huntington's disease (HD)‐associated mHTT is characterized by an expanded polyglutamine (polyQ) tract, which is formed by the cytosine–adenine–guanine (CAG) trinucleotide repeats within the huntingtin (*HTT*) gene. This expansion leads to the formation of misfolded proteins and aggregates that are toxic to neurons [248]. The aggregates disrupt the ubiquitin‐proteasome pathway, as well as transcriptional regulation [249]. This disruption also leads to increased excitotoxicity, impaired mitochondrial function, and changes in energy metabolism. Additionally, it affects axonal transport and synaptic function. These factors collectively contribute to the pathology observed in HD [250]. Exposure to copper intensifies the issues associated with mHTT by reducing the clearance rate of misfolded proteins, thus initiating a detrimental cycle that hastens the process of neurodegeneration [251]. Elevated copper levels have been observed in the striatum of human brains affected by HD after death. However, the reasons for this accumulation of copper in HD and the precise mechanisms by which it could exacerbate neurodegeneration remain unclear [252].

While the full‐length HTT protein is quite large (350 kDa), it is the smaller N‐terminal fragments that primarily drive the progression of the disease [253]. The mHTT protein contains three main domains within its exon 1: the N‐terminal segment (HTT NT), which forms an alpha‐helix; the polyQ domain, which is intrinsically disordered; and the proline‐rich domain, which forms polyproline II helices [254]. The length of the polyQ tract in mHTT is crucial as it determines the protein's propensity to misfold and aggregate. Normally, the HTT has a polyQ tract with less than 36 glutamines, but in HD, this number is expanded to 36 or more, leading to the production of mHTT. The mutant protein with the expanded polyQ tract tends to form beta‐sheet‐rich amyloid‐like fibrils, which are resistant to degradation and can accumulate in cells [255].

To the best of our knowledge, there have been no reports on the interaction between iron and mHTT. In contrast, research has shown that the N171 fragment of mHTT, regardless of whether it contains a typical (17Q) or an elongated (68Q) glutamine stretch, is capable of interacting with Cu^2+^. The interaction with copper was found to be dependent on His residues at positions 82 and 98, which are crucial for binding and appear to work together to coordinate a single copper ion. Additionally, the exon‐1 fragment containing a normal glutamine stretch (17Q, referred to as N84) was also observed to interact with Cu^2+^ [252]. Copper binds to and induces the oxidation of the N171 fragment of HTT, which contains a polyglutamine tract. The formation of copper‐regulated oligomers of HTT is influenced by this oxidative process. Mutations of all cysteine residues present in the N171 fragment prevent the creation of these oligomers. The HTT protein undergoes selective oxidation events that are crucial for the assembly of stable oligomers, and that also slow down their clearance from the soluble cellular compartment [256].

### 2.5 The Downstream Effect of Dysregulated Iron and Copper Metabolism

The dysregulation of cellular iron and copper metabolism can have profound effects on many aspects of cellular functions and biological processes. In the following sections, we discuss the influence of iron and copper dyshomeostasis on oxidative stress/ROS, neuroinflammation, autophagy, organelle interaction network, and cell death.

#### 2.5.1 Oxidative Stress/ROS

**FIGURE 3:**
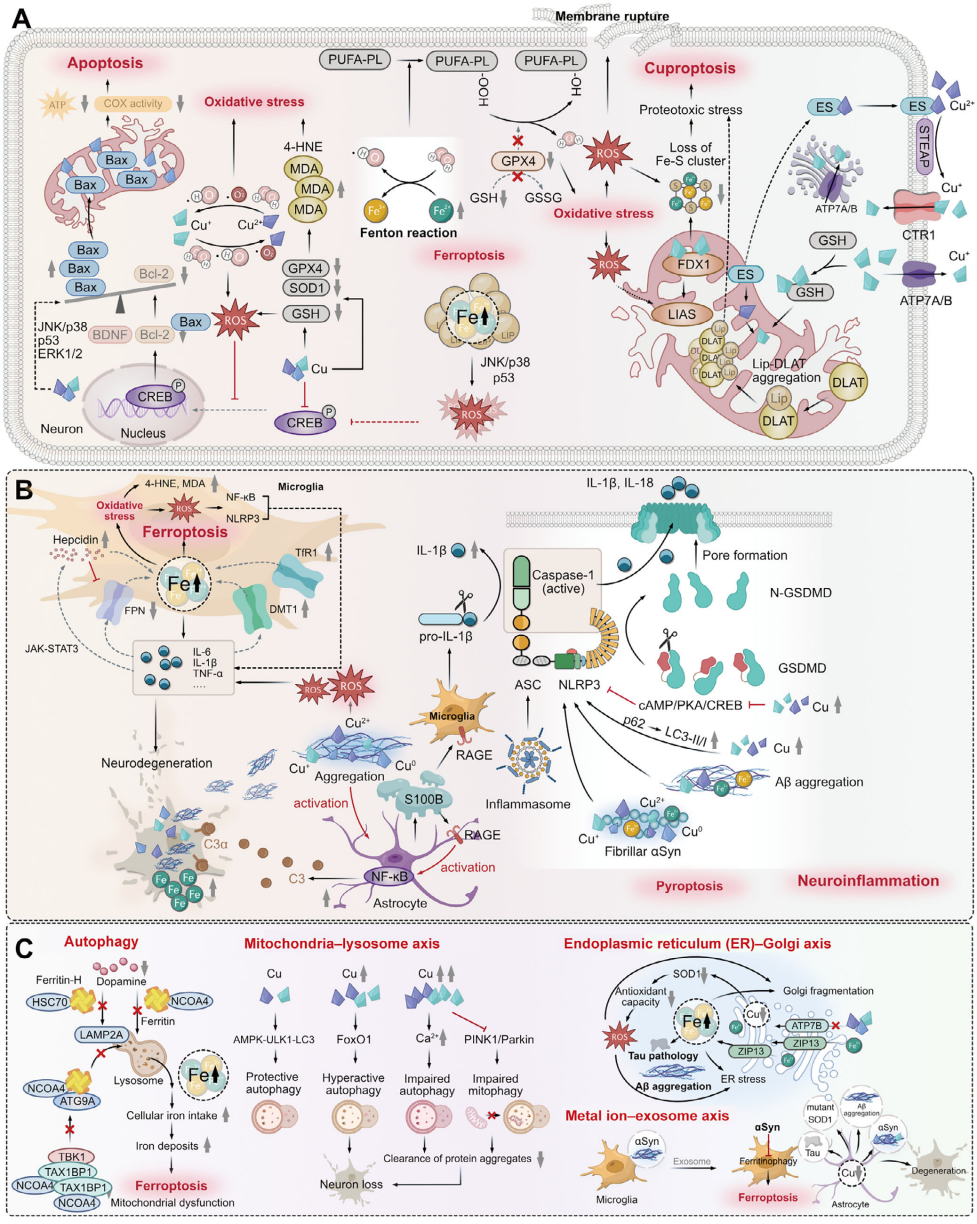
The downstream mechanisms of dysregulated iron and copper metabolism. (A) **Left**: At the cellular level, the redox cycling between Cu^2^^+^ and Cu^+^ induces Fenton and Haber–Weiss reactions, generating ROS and superoxide radicals. Copper dysregulation depletes antioxidants (GSH, SOD1, GPX) and elevates lipid peroxidation markers such as MDA. ROS and copper inhibit the nuclear translocation of p‐CREB and, via the JNK/p38, p53, and ERK1/2 pathways, reduce Bcl‐2 transcription while increasing the Bax/Bcl‐2 ratio. This promotes Bax mitochondrial translocation, intensifies oxidative stress and ROS‐mediated cytotoxicity, and ultimately triggers mitochondrial outer membrane permeabilization and apoptosis. **Middle**: Fe^2^^+^ in the labile iron pool drives the Fenton reaction, producing •OH that attacks PUFAs and initiates a self‐perpetuating lipid peroxidation cascade. Impaired GPX4 function compromises the detoxification of lipid peroxides using GSH, leading to membrane rupture. The resulting lipid peroxidation products (e.g., 4‐HNE, MDA) promote oxidative stress and ROS, which further disrupt Fe–S clusters and impair the mitochondrial respiratory chain, ultimately leading to ferroptosis. Additionally, intracellular iron accumulation induces oxidative stress through the JNK/p38 and p53 pathways, thereby inhibiting CREB phosphorylation. This reduces p‐CREB nuclear translocation, leading to reduced Bcl‐2 transcription, an increased Bax/Bcl‐2 ratio, and ultimately apoptosis. **Right**: Cuproptosis is induced by increasing the concentration of free copper ions in cells. Treatment of cells with copper ionophore, such as ES, facilitates Cu^2+^ entry into cells. In addition, overexpression of the transporter CTR1, which mediates copper entry, or knockdown of the transporter ATP7A/B, which mediates the copper export from cells, can also increase the intracellular Cu^+^ levels. This results in excessive Cu^+^ binding to lipoylated DLAT and further DLAT oligomerization. Together with the copper‐induced decrease in Fe–S cluster stability and the induction of proteotoxic stress, these events lead to copper‐induced cell death, also known as cuproptosis. (B) Microglial iron overload triggers the secretion of inflammatory cytokines such as IL‐6, IL‐1β, and TNF‐α. These cytokines promote intracellular iron accumulation by upregulating iron importers (DMT1, TfR1) and suppressing the iron exporter FPN. Concurrently, they upregulate hepcidin expression via the JAK‐STAT3 pathway, which further induces FPN internalization and degradation. This leads to a state of “iron retention” in microglia, aggravating ferroptosis. Accumulated iron amplifies oxidative stress through the Fenton reaction, generating lipid peroxidation products (e.g., 4‐HNE, MDA) and ROS, which in turn activate the NF‐κB and NLRP3 pathways, forming a self‐perpetuating loop of inflammatory cytokine release. Meanwhile, copper accumulated in aggregates elevates local ROS levels, directly inducing microglial inflammation and, together with plaque aggregates, stimulating astrocytes to release S100B. The released S100B binds to the RAGE receptor, activating NF‐κB, which upregulates S100B and C3 expression, thus creating a self‐amplifying cycle. Activation of the C3a receptor pathway further exacerbates neuronal damage. Elevated S100B also promotes microglial production of pro‐IL‐1β, which is cleaved into active IL‐1β by the NLRP3 inflammasome, sustaining neuroinflammation. Furthermore, copper and iron aggregated with Aβ or αSyn protein aggregates can both activate the NLRP3 inflammasome. Copper overload additionally suppresses the neuroprotective cAMP/PKA/CREB pathway and activates the p62‐dependent autophagy pathway (reflected by an increased LC3II/I ratio), thereby cooperatively promoting NLRP3 inflammasome assembly and activation. Ultimately, these processes converge to drive GSDMD‐mediated pyroptosis, leading to neuronal death. (C) **Left**: Decreased dopamine production not only impairs the recognition of ferritin‐H by HSC70 and its subsequent transport to lysosomes via LAMP2A for degradation, but also inhibits the NCOA4‐mediated delivery of ferritin to lysosomes. Reduced TBK1 expression further hinders ferritin trafficking to lysosomes. Collectively, these disruptions lead to insufficient iron release. To compensate for iron deficiency, cells upregulate iron uptake; however, the accumulated iron cannot be properly metabolized or utilized, ultimately resulting in abnormal intracellular iron accumulation, mitochondrial dysfunction, and ferroptosis. **Middle**: Copper exerts concentration‐dependent effects on autophagy: an appropriate concentration induces protective autophagy; elevated concentrations trigger excessive autophagy via activation of the FoxO1 pathway; and a further increase causes a surge in cytoplasmic calcium concentration, impairing autophagic flux and suppressing mitophagy through inhibition of the PINK1/Parkin pathway. These alterations compromise the cell's ability to clear pathological protein aggregates, leading to cell death. **Right**: ZIP13 promotes iron transport from the cytosol into the ER and Golgi. Excess iron induces oxidative stress, triggering ER stress and Golgi fragmentation, thereby exacerbating Aβ/tau‐related pathology. Reduced ATP7B expression leads to insufficient copper loading in the Golgi, decreasing SOD1 enzymatic activity and weakening antioxidant defenses. Consequently, oxidative stress intensifies and further aggravates ER stress and Golgi structural damage. Microglia can release αSyn via exosomes, promoting its intercellular spread. Aggregated αSyn inhibits ferritinophagy, blocking iron release from ferritin, which impairs cellular iron release and disrupts iron homeostasis, thereby inducing ferroptosis. Mutant CuBP SOD1 enhances exosome release through unconventional secretory pathways, while copper deficiency promotes the spread of misfolded proteins such as Aβ, tau, or αSyn via exosomes, ultimately contributing to neuronal degeneration.

Oxidative stress is a defining characteristic of NDDs. There is a growing body of evidence linking cognitive decline to increased oxidative stress [257]. Oxidative stress needs to be handled delicately in the cell, where its dysregulation can lead to an overproduction of ROS such as hydrogen peroxide (H_2_O_2_), superoxide anion radicals (O_2_˙^−^), and hydroxyl radicals (·OH) [258]. Excessive ROS generation can lead to uncontrolled oxidative damage to proteins, lipids, and DNA within cells [259]. Both iron and copper are redox‐active metals that are closely linked to the cellular redox balance and oxidative stress (Figure 3A).

##### 2.5.1.1 Iron‐Induced Oxidative Stress/ROS

Accumulated iron catalyzes the production of ROS and oxidative stress via the Haber–Weiss and Fenton reactions, resulting in a reduction in ATP synthesis, decreased activity of COX, increased permeability of the inner mitochondrial membrane, damage to the outer mitochondrial membrane, and collapse of the mitochondrial membrane potential [260, 261]. Among the various ROS, O_2_˙^−^ holds particular significance, as it can initiate a vicious cycle of iron‐mediated oxidative stress. It reduces Fe^3^^+^ to the more reactive Fe^2^^+^ via the reaction: Fe^3^^+^ + O_2_˙^−^ → Fe^2^^+^ + O_2_. The generated Fe^2^^+^ further participates in the Fenton reaction, which catalyzes the formation of the highly reactive •OH [262] (Figure 3A).

Mechanically, excess iron depletes GSH, impairs key antioxidant proteins such as glutathione peroxidase 4 (GPX4), and indirectly suppresses others, including superoxide dismutase (SOD1) and catalase. The generated radicals trigger lipid peroxidation of polyunsaturated fatty acids (PUFAs) within cell membranes, generating toxic byproducts such as malondialdehyde (MDA) and 4‐hydroxynonenal (4‐HNE). The resulting oxidative stress enhances ROS production, exacerbates damage to Fe–S clusters, mitochondrial respiratory complexes, and cell membranes, and ultimately promotes neuronal apoptosis or ferroptosis in susceptible brain regions [263].

##### 2.5.1.2 Copper‐Induced Oxidative Stress/ROS

The cycling of copper between its two oxidation states, Cu^2+^ and Cu^+^, is a critical factor in copper homeostasis, and its interconversion could initiate the Haber–Weiss and Fenton reactions, leading to ROS production [264] (Figure 3A). Elevated copper levels can impair mitochondrial function by causing oxidative stress and ROS, similarly to the effect of iron overload [261, 265, 266]. Like iron, an excess of copper results in the depletion of GSH and the inhibition of key antioxidant enzymes, thereby amplifying oxidative stress and damage from lipid peroxidation [267, 268].

The underlying mechanism of copper‐induced oxidative stress/ROS may involve the cAMP response element‐binding protein (CREB) [269]. Within the brain, CREB is a key transcription factor associated with neuronal survival and learning capacity, commonly referred to as the “memory switch” [270]. Unlike iron overload, which suppresses CREB through ROS induction [271], direct copper exposure or copper‐induced oxidative damage can hinder CREB phosphorylation, resulting in reduced expression of brain‐derived neurotrophic factor (BDNF) and the antiapoptotic Bcl‐2 (B‐cell lymphoma 2) protein. Subsequently, a decrease in the Bcl‐2/Bax (Bcl‐2 associated X protein) ratio facilitates the translocation of proapoptotic Bax to the mitochondria, leading to diminished mitochondrial membrane potential and the induction of cellular apoptosis [269].

#### 2.5.2 Neuroinflammation

NDDs are characterized by chronic neuroinflammation that does not resolve spontaneously [272]. Neuroinflammation is primarily mediated by microglia and astrocytes, which release proinflammatory cytokines, chemokines, signaling molecules, and ROS [273, 274]. In the aging brain, microglia become functionally impaired and prone to chronic activation, potentially facilitating NDD development [275].

##### 2.5.2.1 Iron‐Induced Neuroinflammation

Iron plays a critical role in mediating neuroinflammation in the CNS through a multifaceted mechanism, which primarily involves microglial activation (Figure 3B). Microglia, as the most efficient iron‐absorbing glial cells, are directly stimulated by iron to release proinflammatory cytokines, such as interleukin (IL)‐6, IL‐1β, and tumor necrosis factor (TNF)‐α, thereby initiating the neuroinflammatory response [276]. These cytokines, particularly IL‐6, can upregulate the expression of hepcidin via the JAK/STAT3 pathway. Hepcidin subsequently binds to and leads to the internalization and degradation of the iron exporter FPN, resulting in the block of cellular iron efflux and increased intracellular iron accumulation [277]. Concurrently, these proinflammatory signals also promote the upregulation of neuronal iron import proteins, such as DMT1 and TfR1, which leads to neuronal iron overload [278, 279]. This excess of iron within neurons further aggravates the situation by exacerbating microglial activation and the release of proinflammatory factors [278]. Microglia are highly susceptible to iron‐induced cell death, with this susceptibility to ferroptosis being influenced by the vesicular transport gene SEC24B [280]. This positions ferroptosis as a critical pathway connecting iron overload to microglial loss and neurodegeneration [280]. Additionally, iron accumulation drives a phenotypic transition in microglia from a protective M2 state to a harmful proinflammatory M1 state [281].

The resulting inflammatory and pro‐oxidant milieu is amplified through several key pathways. Iron overload and the ensuing oxidative stress foster the generation of lipid peroxidation products and cellular debris, which can accelerate the aggregation of pathological proteins (including Aβ and αSyn), thereby exacerbating proteopathy and inflammatory responses [263]. ROS activate the nuclear factor‐kappa B (NF‐κB) pathway and the NLRP3 inflammasome, driving the secretion of IL‐1β and IL‐18 and establishing a proinflammatory feedback loop [282]. This reinforced inflammatory environment reinforces the mechanisms of cellular iron retention and disrupts BBB integrity, creating a self‐perpetuating cycle that sustains and intensifies neuroinflammation [283].

##### 2.5.2.2 Copper‐Induced Neuroinflammation

Copper can also induce inflammation in brain tissue, potentially contributing to the BBB breakdown and toxin accumulation associated with the development of NDDs [6]. Research indicates that copper deficiency in rodent models increases vulnerability to inflammatory stimuli [284]. Both copper deficiency and accumulation in the CNS have been reported to activate microglia and astrocytes, with elevated labile copper levels observed in microglia during neuroinflammation [285]. It is suggested that the regulation of copper homeostasis plays a role in modulating the transition of microglia from proinflammatory to anti‐inflammatory states, which is mediated through modulating NO and S‐nitrosothiol signaling [286].

Furthermore, excess copper accumulation in the CNS induces the misfolding and aggregation of pathological proteins (such as Aβ and αSyn), which further cause astrocytes to release elevated levels of several cytokines, including the proinflammatory S100 protein family, particularly S100B [287] (Figure 3B). S100B proteins function as extracellular cytokines via signaling pathways mediated by the receptor for advanced glycation end (RAGE) products. The interaction between S100B and RAGE exhibits a clear concentration dependence. At low concentrations, which are typically found under physiological or low‐pathological conditions, it generally exerts protective effects. At high concentrations, often observed in pathological states, this interaction shifts toward proinflammatory and neurotoxic roles [288]. Once activated and present at high micromolar concentrations, the continuous interaction of S100B with RAGE further raises extracellular S100B levels by activating NF‐κB, creating a self‐sustaining positive feedback loop within astrocytes [289]. Astrocyte‐secreted S100 proteins also contribute to the priming and activation of the NLRP3 inflammasome, promoting the production of active IL‐1β in neuroinflammatory conditions [290]. As a proinflammatory cytokine, IL‐1β fosters an inflammatory milieu around the plaques, which hinders their degradation and clearance by microglia [291]. Moreover, IL‐1β has the potential to induce synaptic impairment, neuronal apoptosis, and the suppression of neurogenesis [292]. In addition, harmful protein aggregates activate the NF‐κB signaling cascade in astrocytes, leading to increased secretion of complement C3, which subsequently engages with C3a receptors on neurons, causing neuronal damage [293].

#### 2.5.3 Influence on Autophagy

Autophagy, an evolutionarily conserved intracellular degradation system, plays a pivotal role in maintaining brain homeostasis [294]. Impairments of the autophagy–lysosomal pathway (ALP) are commonly observed in multiple NDDs [295, 296]. Iron and copper have been implicated in the pathological mechanisms of NDDs through their regulation of autophagic processes [297].

##### 2.5.3.1 Influence of Iron on Autophagy

Dysregulation of iron metabolism significantly influences the progression of NDDs through the “ferritinophagy” pathway, an autophagic form that selectively degrades the cellular iron‐storage protein ferritin (Figure 3C). NCOA4 serves as the key receptor that facilitates ferritinophagy, which binds to ferritin and directs it into the ALP for degradation, thereby releasing stored iron [298]. In conditions where iron is abundant, NCOA4 undergoes degradation, leading to a reduction in ferritinophagy; conversely, under iron‐deficient conditions, NCOA4 is stabilized, thereby promoting ferritinophagy and iron release [299]. Therefore, NCOA4‐mediated ferritinophagy leads to an increase in the intracellular LIP and enhances cellular susceptibility to ferroptosis [300]. In the neuroferritinopathy cell model, the accumulation of defective ferritin results in a decrease in NCOA4 levels, which favors the formation of ferritin/iron aggregates, leading to the onset of cellular senescence phenotype or increased susceptibility to ferroptosis in neurons [301]. In summary, these findings demonstrate that the NCOA4‐mediated ferritinophagy pathway should be carefully controlled. Furthermore, iron overload can impair the autophagic flux in microglia by reducing Rab7, thereby disrupting autophagosome–lysosome fusion, which may contribute to sustained inflammation [302].

Under normal physiological conditions, serine/threonine‐protein kinase (TBK1) forms a complex with tax1‐binding protein 1 (TAX1BP1) and NCOA4. The kinase activity of TBK1 phosphorylates ferritin, promoting its dissociation from the complex. Subsequently, NCOA4 cooperates with autophagy‐related protein ATG9A to transport ferritin to lysosomes for degradation, thereby releasing ferrous ions and maintaining intracellular iron homeostasis [303]. However, in ALS patients, the expression or function of TBK1 is reduced. This impairment prevents ferritin from being efficiently delivered to lysosomes, resulting in insufficient release of ferrous ions. To compensate for the iron deficiency, cells upregulate iron uptake. However, the accumulated iron may not be properly metabolized and utilized, ultimately leading to abnormal intracellular iron accumulation [303].

Within astrocytes, in addition to the NCOA4‐mediated canonical ferritinophagy, dopamine can also promote a chaperone‐mediated autophagy pathway, in which ferritin is recognized by heat shock cognate 71 kDa protein (HSC70) and is subsequently transported via lysosome‐associated membrane glycoprotein 2 (LAMP2) to lysosomes for degradation [304]. The regulated degradation of ferritin and the proper release of iron are essential for the maintenance of mitochondrial function. In PD patients, decreased dopamine levels in the brain hinder the degradation of ferritin, thereby contributing to mitochondrial dysfunction in astrocytes [304].

##### 2.5.3.2 Influence of Copper on Autophagy

Copper exhibits complex, concentration‐dependent effects on autophagy (Figure 3C). In some contexts, such as in AD, adequate intracellular copper concentration is essential for inducing protective autophagy [294]. Increased copper import in the neuroblastoma SK‐N‐SH cell line enhances AMPK‐ULK1 signaling, p62 expression, and ATG5‐dependent autophagy as a protective mechanism [305]. This is reproduced in a mouse model that chronic copper exposure can increase the AMPK phosphorylation and the autophagy marker LC3‐II in a dose‐dependent manner [306]. Furthermore, high concentration copper triggers excessive autophagy in neural stem cells by activating the forkhead box protein O1 (FoxO1) pathway [307].

However, excess copper can also promote pathological outcomes by inhibiting autophagic flux in MN9D mouse dopaminergic neuronal cells. This inhibition is likely achieved through the induction of intracellular calcium dyshomeostasis, the dysregulation of transcription factor EB (TFEB) activity, and the impairment of lysosomal function [308]. Sustained copper exposure in the murine microglial BV2 cell line also inhibits the mitophagy process [309], which is a selective autophagic mechanism for the removal of damaged and depolarized mitochondria and is mediated by serine/threonine‐protein kinase PINK1 and E3 ubiquitin‐protein ligase Parkin [310]. The blockage of mitophagy in microglial cells results in the accumulation of damaged mitochondria and sustained release of inflammatory factors [309].

In summary, under physiological conditions, autophagy exerts a protective effect by clearing abnormal protein aggregates [311], but in the pathological context of disrupted iron and copper homeostasis, autophagy may become hyperactivated or impaired, ultimately contributing to neuronal loss [312]. Targeting specific autophagy pathways regulated by iron and copper may offer novel therapeutic strategies for NDDs.

#### 2.5.4 Influence on Organelle Interaction Network

Imbalances in iron and copper metabolism can significantly disrupt the interactions and communications between various organelles, including but not limited to the mitochondria–lysosome axis, ER–Golgi axis, and exosomes (Figure 3C). The interorganellar cross‐regulations play important roles in maintaining cellular homeostasis and biological functions, involving mechanisms such as the exchange of molecules and signals. Disruptions of the organelle interaction network may contribute to oxidative stress, protein aggregation, and neuronal death, all of which are important factors in NDDs [6].

##### 2.5.4.1 Influence of Iron and Copper on the Mitochondria–Lysosome Axis

The mitochondria–lysosome axis plays a critical role in the maintenance of cellular functions, which include mitochondrial quality control, mitophagy, and lysosomal function. Mitochondria regulate cellular respiration, energy production, cell survival, and death, while lysosomes are responsible for protein degradation, nutrient sensing, and metabolic signaling. The bidirectional regulation of their dynamics and network structure is critical for maintaining neuronal homeostasis [313].

Iron accumulation within mitochondria can trigger oxidative stress, leading to excessive mitochondrial fission mediated by dynamin‐related protein 1 (DNM1L), which ultimately results in mitochondrial damage and neuronal death [314]. Proteins located at the mitochondria–lysosome contact sites, such as Parkin and PINK1, are key regulators of mitochondrial health, overseeing processes like mitophagy, which is a selective degradation process of damaged mitochondria by lysosomes [315]. The dysregulations of mitochondria–lysosome contact sites have been implicated in the pathogenesis of NDDs [316]. Notably, *PINK1* is recognized as a risk gene associated with PD, and its mutations increase susceptibility to the disease by inducing mitochondrial dysfunction [313]. In the *Drosophila* model, modulating iron homeostasis within mitochondria, by either overexpressing the mitochondrial iron importer mitoferrin or knocking down the FeBP ferritin, effectively rescues multiple phenotypes associated with the loss‐of‐function of PINK1 [317].

Copper overload leads to mitochondrial dysfunction and triggers compensatory mitophagy that is dependent on the PINK1/Parkin pathway (Figure 3C). However, prolonged exposure to copper impairs the fusion and degradation capacities of lysosomes, inhibiting mitophagic flux and resulting in the accumulation of damaged mitochondria [318]. In PD models, dysregulated copper homeostasis adversely affects mitochondrial dynamics and lysosomal acidification, thereby hindering communication between these two organelles [319].

##### 2.5.4.2 Influence of Iron and Copper on the ER–Golgi Axis

The ER–Golgi axis is a critical network involved in protein folding, trafficking, glycosylation, and the integrity of the secretory pathway [320]. The disruption of the ER–Golgi axis may result in ER stress, fragmentation of the Golgi apparatus, impaired vesicular transport, and heightened neuronal vulnerability, all of which significantly contribute to the development of NDDs [320].

The ER and Golgi compartments maintain distinct iron pools essential for proper protein maturation (Figure 3C). The transporter ZIP13 (encoded by *SLC39A13*) facilitates the movement of iron from the cytosol into the lumen of the ER and Golgi, thus maintaining interorganellar homeostasis [321]. However, in AD models, excess iron has been shown to induce oxidative stress, which in turn triggers ER stress through the activation of the unfolded protein response (UPR). This process can further cause Golgi fragmentation by disrupting microtubule dynamics or phosphorylation cascades [322]. In PD models, iron overload has been shown to compromise vesicular trafficking from the ER to the Golgi apparatus, thereby exacerbating the accumulation of αSyn and obstructing secretory pathways [323]. Fragmentation of the Golgi apparatus due to regional iron accumulation can result in reduced glycosylation of key proteins, such as APP, and elevated ER stress, thereby exacerbating the pathologies associated with Aβ/tau [324].

The ER–Golgi axis also plays a critical role in maintaining intracellular copper homeostasis to support the proper function of copper‐dependent enzymes. Excess free copper can generate ROS, which directly triggers ER stress and contributes to Golgi fragmentation [325]. This fragmentation then blocks the vesicular trafficking from the ER to the Golgi, leading to the accumulation of misfolded proteins and an increase in toxicity [326]. Under steady‐state conditions, the copper‐transporting P‐type ATPases, ATP7A and ATP7B, are mainly localized to the Golgi apparatus. In models of copper homeostasis disruption, such as Wilson's disease (WD) and Menkes disease (MD), the functional defects of ATP7A/B result in insufficient copper loading within the Golgi lumen. This not only interferes with copper‐dependent secretory pathways and protein trafficking but also reduces the activity of enzymes such as SOD1, which compromise the antioxidant defense capacity [118]. Consequently, oxidative stress is exacerbated, creating a vicious cycle that exacerbates ER stress [327]. Ultimately, disturbances in regional brain copper distribution and dysfunction of the ER–Golgi axis jointly promote the progression of NDDs [328].

##### 2.5.4.3 Influence of Iron and Copper on Intercellular Signaling Through Exosomes

Exosomes play a critical role in facilitating intercellular communication in NDDs by transporting pathogenic proteins, microRNAs, and inflammatory mediators, thereby contributing to the spread of protein, neuroinflammation, and neuronal death [329].

Specifically, exosomes promote iron export by secreting ferritin‐bound iron, which reduces intracellular LIP levels, thereby conferring resistance to ferroptosis in stressed cells [330]. Currently, evidence does not support the notion that free or ferritin‐bound iron within exosomes acts as a direct signal to activate signaling cascades; rather, their effects are mediated by cargo components, such as miRNAs and proteins, that influence ferroptosis pathways [329]. In the context of NDDs, exosomes are involved in the transmission of pathogenic proteins between cells (Figure 3C), which may indirectly exacerbate iron dyshomeostasis and increase susceptibility to ferroptosis [6]. Microglia can release αSyn via exosomes, thereby promoting its propagation between cells in PD [331]. Aggregated αSyn can inhibit ferritinophagy, thereby blocking iron release from ferritin [332]. Thus, αSyn transmitted via exosomes may indirectly lead to impaired release of cellular iron and disruption of iron homeostasis, while microglia are highly susceptible to ferroptosis [333]. Furthermore, in oligodendrocytes, Fth can be secreted via exosomes, which is of great importance for maintaining antioxidant function [334].

Copper ions have also been shown to influence exosome production and content in specific cellular contexts associated with NDDs, primarily through CuBPs like mutant SOD1 in an indirect manner [335]. In ALS astrocytes, mutant SOD1 enhances exosome release via unconventional secretory pathways, facilitating the intercellular transfer of toxic mutant SOD1 to neurons and contributing to non–cell‐autonomous motor neuron degeneration [336]. In AD and PD, copper dyshomeostasis exacerbates pathology, with exosomes facilitating the spread of misfolded proteins such as Aβ, tau, or αSyn, which may be influenced by altered copper homeostasis that increases oxidative stress and protein aggregation. However, the specific induction of neural exosome production by copper is not robustly established [337]. Recent studies have also underscored the roles of copper‐related protein targets, such as dihydrolipoyllysine‐residue acetyltransferase (DLAT), in the dynamics of microglial exosomes under conditions of copper toxicity in AD models, suggesting that exosomes may amplify the effects of copper dyshomeostasis [338].

#### 2.5.5 Iron/Copper‐Induced Cell Death

##### 2.5.5.1 Apoptosis

Apoptosis is a form of regulated cell death (RCD) mediated by both the intrinsic and extrinsic pathways, typically characterized by phosphatidylserine externalization and caspase activation. The extrinsic pathway is initiated by the interaction between TNF ligand superfamily member 6 (FASL) and TNF receptor superfamily member 6 (FAS), which recruits FAS‐associated death domain (FADD) protein and activates caspase‐8. The intrinsic pathway, triggered by mitochondrial damage signals such as excessive ROS and calcium overload, leads to the release of cytochrome c or apoptosis‐inducing factor 1 (AIFM1), subsequently activating caspase‐9 or endonucleases, respectively. These two pathways converge to activate effector caspases, which cleave cellular components to execute apoptosis. The mitochondrial membrane potential, regulated by the Bcl‐2 protein family, plays a critical role in the release of mitochondrial proteins [294].

In cellular models and animal models, overload of iron or copper has been shown to increase the expression of proapoptotic proteins, such as Bax and cleaved caspase‐3, while simultaneously reducing the levels of Bcl‐2. This imbalance contributes to increased oxidative damage, lipid peroxidation, and neuronal degeneration [339, 340]. Iron or copper overload is also associated with DNA fragmentation and the upregulation of p53‐mediated apoptotic proteins, which in turn can lead to neuroinflammation and neuronal death [341, 342]. Iron overload is also implicated in cellular apoptosis through the accumulation of ROS mediated by the JNK/p38 MAPK pathway, which results in DNA damage and the subsequent activation of the ATM/p53 pathway [343]. Similarly, copper overload can induce apoptosis through the p38/MAPK and ERK1/2 pathways [344] (Figure 3A).

Moreover, iron accumulation can activate ER stress, leading to the aberrant activation of the IRE1α pathway. The enhanced activation of IRE1α promotes the expression of XBP1s, ultimately resulting in ER stress‐related apoptosis [345]. Similarly, copper overload initiates the UPR, characterized by the upregulation of ER stress sensor pathways, particularly PERK and IRE1α. This response is associated with an increase in the expression of ER stress‐related proteins, including PERK, XBP1, and CHOP, ultimately leading to cellular apoptosis and the inhibition of autophagy [346].

##### 2.5.5.2 Pyroptosis

Pyroptosis is a caspase‐1‐dependent, inflammatory RCD characterized by pore formation in the plasma membrane. Morphologically, it leads to cell swelling, lysis, and the release of proinflammatory cytokines [347]. The initiation of pyroptosis occurs through the activation of inflammasomes, notably the NLRP3‐ASC (apoptosis‐associated speck‐like protein containing a CARD) complex, which serves as a molecular platform for caspase‐1 activation [348]. Once activated, caspase‐1 cleaves gasdermin D (GSDMD), releasing its N‐terminal pore‐forming domain. This results in membrane perforation and the secretion of inflammatory cytokines such as IL‐1β and IL‐18 [349, 350].

Pyroptosis is implicated in multiple NDDs. In AD, Aβ aggregate activates NLRP1 and NLRP3 inflammasomes to induce neuronal pyroptosis [351], while in PD, fibrillar αSyn triggers NLRP3 inflammasome‐mediated neuroinflammation and pyroptosis [352]. Additionally, the NLRP3/GSDMD‐mediated pyroptosis pathway also contributes to the pathogenesis of multiple sclerosis (MS) [353].

Evidence suggests that copper can promote pyroptosis (Figure 3B). In an AD cell model of mouse hippocampal HT‐22 cells cultured with Aβ_42_, CuCl_2_ treatment promoted Aβ aggregation and upregulated the expression of key proteins associated with pyroptosis, including NLRP3, ASC, caspase‐1, cleaved caspase‐1, GSDMD, and GSDMD N‐terminal (N‐GSDMD) [354]. Moreover, this Cu^2^^+^‐mediated pyroptosis in HT‐22 can be partially mitigated by the NLRP3 inhibitor MCC950 [354]. Furthermore, 8 weeks of free drinking with up to 500 mg/L copper can induce the pyroptosis of dopaminergic neurons in the substantia nigra pars compacta (SNpc) of mice [309]. Copper is reported to downregulate the neuroprotective cAMP/PKA/CREB signaling pathway, and simultaneously induce oxidative stress that activates the NLRP3 inflammasome, ultimately resulting in GSDMD‐mediated pyroptosis in MN9D dopaminergic neuronal cells [355]. In addition, sustained copper exposure can also increase p62 expression and LC3‐II/I ratio, thereby activating the NLRP3/caspase‐1/GSDMD‐dependent pyroptosis pathway in BV2 microglial cells [309].

##### 2.5.5.3 Ferroptosis

Ferroptosis is a distinct mode of RCD predominantly driven by iron‐dependent lipid peroxidation, which results in membrane rupture and mitochondrial shrinkage [356]. The process is primarily characterized by the peroxidation of PUFAs present in phospholipid bilayers [357] (Figure 3A). Reactive Fe^2^^+^ from the LIP generates hydroxyl radicals through the Fenton reaction, thereby initiating a chain reaction of lipid peroxyl radicals and hydroperoxides. In addition, these peroxides can also be enzymatically produced by lipoxygenases and cytochrome P450 oxidoreductase [356]. The selenoprotein GPX4 plays a protective role by using GSH to reduce lipid hydroperoxides to nontoxic alcohols [358].

Confirming the presence of ferroptosis within the brain remains a complex endeavor, largely due to the lack of specific biomarkers. Current diagnosis relies on indirect indicators such as iron accumulation, elevated levels of lipid peroxidation markers (e.g., 4‐HNE, MDA), and reduced GSH levels. Definitive confirmation requires the reversal of cell death through the usage of ferroptosis inhibitors, which may include iron chelators or radical‐trapping agents [356]. Recently, Zha et al. have identified a gut–microbiome–brain axis in which microbiota‐derived lysophosphatidylcholine (LPC) sustains the expression of the ferroptosis regulator long‐chain‐fatty‐acid–CoA ligase 4 (ACSL4), thereby suppressing ferroptosis and alleviating the AD pathology [359].

##### 2.5.5.4 Cuproptosis

Cuproptosis, a merging concept of Cu‐dependent RCD, was first introduced by Tsvetkov et al. in 2022 [360]. Cuproptosis is triggered by the cellular overaccumulation of copper ions, mainly involving the regulation of ferredoxin 1 (FDX1), lipoic acid synthase (LIAS), and DLAT [7] (Figure 3A). FDX1 not only reduces Cu^2+^ to Cu^+^, but also is an upstream regulator of protein lipoylation that promotes the lipoylation of several enzymes, including DLAT, a key component of the pyruvate dehydrogenase complex (PDHC) that regulates the metabolic flux in the tricarboxylic acid (TCA) cycle in mitochondria [361]. LIAS, through interaction with FDX1, promotes mitochondrial protein lipoylation by catalyzing the final step in the de novo synthesis of lipoic acid [362]. The reduction of Cu^2+^ to the more toxic Cu^+^ by FDX1 can contribute to the aberrant oligomerization of lipoylated proteins, including DLAT, and the destabilization of Fe–S cluster proteins, thus lowering the overall activity of the TCA cycle. The reduction of Fe–S cluster protein stability further disrupts genomic stability and gene expression, affects the ubiquitination degradation pathway, and ultimately leads to proteotoxic stress and cell death [363].

Although there is no direct evidence that cuproptosis occurs in the course of NDDs, the association between cuproptosis and NDDs is emerging [364]. Zhang et al. reported that copper induces cognitive impairment in mice via the modulation of cuproptosis [365]. Exposure of male mice to copper in their drinking water at 4 weeks of age for 3 months exacerbated learning and memory impairment capabilities and resulted in an accumulation of copper in the brain and urine. The study revealed that copper exposure increased the expression of the cuproptosis‐related proteins FDX1 and DLAT, and decreased the levels of Fe–S cluster proteins, resulting in oxidative damage and neuronal degeneration [365].

## 3 The Implications of Dysregulated Iron and Copper Metabolism in NDDs

Dysregulation of iron and copper homeostasis has been associated with various NDDs. In this section, we summarize the role of abnormal iron and copper levels and dysregulated iron and copper metabolism in the development of eight major NDDs including Alzheimer's disease (AD), Parkinson's disease (PD), Prion diseases (PrDs), HD, WD, MD, amyotrophic lateral sclerosis (ALS), and MS (Tables 3 and 4).

**TABLE 3:** Summary of compounds that ameliorate NDDs through modulation of iron/copper homeostasis.

Intervention	Compound	Structure	Model	Pharmacological effect	Disease	References
Iron chelator	Deferoxamine (DFO)	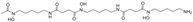	P301L tau transgenic mice and APP/PS1 mice	Inhibit ferroptosis, reduce oxidative stress, and slow down cognitive decline	AD	[366]
			PC12 cells	Enhance dopaminergic neuron survival, and suppress αSyn aggregation	PD	[367]
	Deferiprone (DFP)	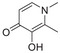	rTg4510 mice	Reduce brain iron deposition, lower hyperphosphorylated tau levels, and improve behavioral performance	AD	[368]
			A53T transgenic mice	Rescue behavioral deficits by inhibiting iron‐induced αSyn aggregation and exacerbate oxidative damage	PD	[369]
			MPTP‐treated mouse model	Counteract free iron toxicity, elevate striatal dopamine levels, and mitigate ferroptosis‐like features and neurodegeneration	PD	[370]
	PBT434	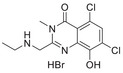	hBMVEC	Modulate the cellular iron uptake by chelation of extracellular Fe^2+^	AD, PD	[371]
			6‐OHDA toxin mouse model, MPTP‐treated mouse model, hA53T transgenic mice	Lower nigral αSyn accumulation, and rescue motor performance	PD	[372]
Multifunctional ion chelator	M30	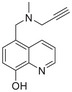	SH‐SY5Y cells, NSC‐34 cells, primary cortical cells	Increase striatal dopamine levels and enhance dopaminergic neuron populations and TfR‐positive cells	AD, PD, ALS	[373]
	VK‐28	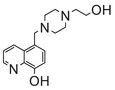	SOD1^G93A^	Prevent motor neuron degeneration	ALS	[374]
	VAR10303	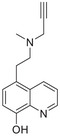	6‐OHDA (rat) and MPTP (mouse) models	Mitigate striatal dopamine depletion, decrease dopamine turnover, and upregulate tyrosine hydroxylase levels	PD	[375]
	GW501516	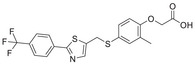	MPTP‐treated mouse model	Alleviate motor deficits, protect dopaminergic neurons, reduce striatal dopamine loss, and suppress neuroinflammation	PD	[376]
Natural compounds	Curcumin	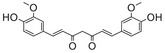	Tg2576 transgenic mice and	Bind Fe^2^^+^, reduce amyloid and oxidized protein aggregation, and improve cognitive deficits	AD	[377]
	Eriodictyol	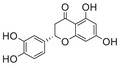	Aβ_42_‐induced HT22 cells, APP/PS1 mice	Inhibit tau phosphorylation, activate the Nrf2/HMOX1 pathway to combat ferroptosis, and ameliorate cognitive deficits	AD	[378]
	Sterubin	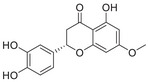	HT22 hippocampal nerve cells	Enhance mitochondrial homeostasis, calcium uptake, biogenesis, fusion/fission balance, and respiratory function, and improveS bioenergetic efficiency	AD	[379]
	Fisetin	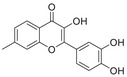	HT22 hippocampal nerve cells	Enhance mitochondrial homeostasis, calcium uptake, biogenesis, fusion/fission balance, and respiratory function, and improve bioenergetic efficiency	AD	[379]
	Berberine	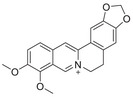	3×Tg AD mice	Modulate iron levels, inhibit ferroptosis, and activate the Nrf2 pathway	AD	[380]
	Naringin	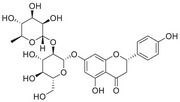	Iron dextran‐induced iron‐overloaded mice	Reduce nonheme iron and decrease amyloid plaque formation	AD	[381]
	DL‐3‐n‐butylphthalide	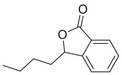	Rotenone‐treated rats	Reduce motor deficits, dopaminergic neuron loss, and iron deposition	PD	[382]
	Quercetin	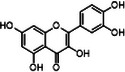	Erastin and RSL‐3 induced M17 cells, PC12 and SH‐SY5Y cells, MPP^+^ induced PC12 cells, MPTP‐treated mouse model	Exert antiferroptosis effects via Nrf2 activation	PD	[383]
	Epigallocatechin gallate (EGCG)	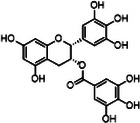	*αSyn* gene‐transduced PC12 cells	Inhibit Fe^3^^+^‐induced αSyn conformational changes and protect cells from Fe^3^^+^ toxicity	PD	[384]
Copper ionophore	Cu^II^(gtsm)	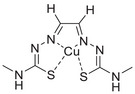	APP/PS1 and rTg4510 mouse models	Reduce tau pathology and increase the structural component of PP2A	AD	[385]
	Elesclomol (ES)	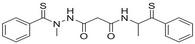	Mottled‐brindled mice	Improve whisker appearance from bushy, highly kinked clumps	MD	[386]
			VPS13∆ yeast strain	Alleviate the impairment	MD	[387]
MPAC	Clioquinol (CQ)	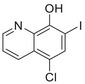	APP2576 transgenic mice	Decrease in brain Aβ deposition	AD	[388]
			MPTP‐induced mouse model	Reduce the loss of SN neurons	PD	[389]
			PC12 cells expressing polyQ‐*Htt*, R6/2 transgenic Huntington's mice	Reduce mutant protein level and decrease cell death; mitigate pathology and behavioral abnormalities	HD	[390]
			EAE mouse model	Suppress demyelination and reduce infiltration by encephalitogenic immune cells	MS	[391]
	PBT2	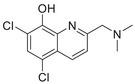	APP/PS1 and Tg2576 transgenic mouse models	Improve synaptic health and reduce Aβ burden and tau phosphorylation	AD	[392]
			R6/2 mouse model	Reduce striatal atrophy and improve motor performance	HD	[393]
	INHHQ	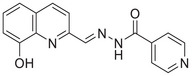	H4 (human neuroglioma) cells	Disrupt anomalous copper‐αSyn interactions via metal ions sequestering	PD	[394]
Copper chelator	DPA	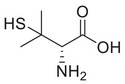	AD patients	Reduce serum oxidative stress, but no difference in cognitive decline	AD	[395]
	TDMQ20	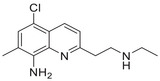	icv‐CuAβ mouse model, hippo‐CuAβ mouse model, 5XFAD mouse model	Reverse cognitive and behavioral impairments, neutralize oxidative stress in the cortex	AD	[396]
	Cu^II^(atsm)	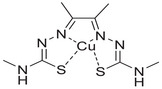	MPTP‐induced mouse model	Rescue the loss of dopaminergic cells, improve motor dysfunction, and extend survival rates	PD	[397]
			SOD1^G37R^ mice, SOD1^G93A^ mice	Boost SOD1 activity, retard paralysis onset, and extend mouse lifespan	ALS	[398]
	Trientine	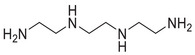	WD patients	Mobilize liver copper and promote urinary excretion	WD	[399, 400]
	*N*,*N*‐Dimethyldithiocarbamate (DMDTC)	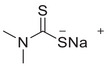	Macular mouse model	Increase brain copper levels and prolong lifespan	MD	[401]
Copper–protein binding agent	Tetrathiomolybdate (TTM)	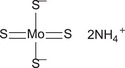	WD patients	Reduce intestinal copper uptake to a clinically significant degree	WD	[402]
	ALXN1840	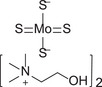	WD patients	Reduce copper levels in the liver and blood through biliary excretion	WD	[403]
Natural compound	Myricetin	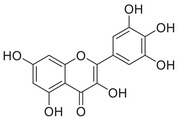	CuCl_2_‐induced SK‐N‐BE(2)‐M17 cells	Inhibit Aβ aggregate formation, reduce copper‐induced Aβ cytotoxicity	AD	[404]
	Apigenin	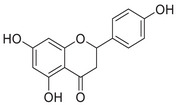	Copper‐treated APPsw overexpressing cells	Protect neurons from toxicity caused by Aβ in the presence of copper	AD	[405]
	Quercetin	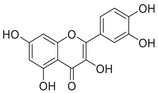	CuSO_4_‐induced SH‐SY5Y cells	Reduce αSyn levels	PD	[346]
	Epigallocatechin gallate (EGCG)	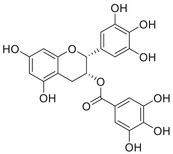	*αSyn* gene transduced PC12 cells	Hinder αSyn conformation change and ROS generation; suppress αSyn overexpression and fibrillation	PD	[406]
	Xyloketal B	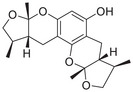	*Caenorhabditis elegans* Huntington's disease model	Prevent the formation of mutant Htt aggregates	HD	[407]
	Neferine	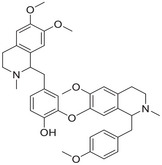	Overexpressing huntingtin with 74 CAG repeats in PC12 cells	Reduce mutant Htt through an autophagy‐related gene 7 (Atg7)‐dependent mechanism	HD	[408]
	Morroniside	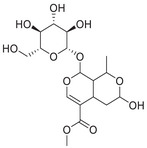	H_2_O_2_ induced SH‐SY5Y cells	Inhibit the decreased SOD1 activity caused by H_2_O_2_	ALS	[409]
	Chrysophanol	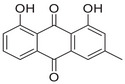	Rats receiving dietary copper supplementation for 12 weeks	Reduce the liver copper, increase the 24 h urine copper, improve the learning and memory ability, alleviate hippocampal neuron damage and apoptosis	WD	[410]
	Ursolic acid	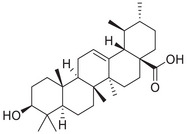	Cuprizone‐induced demyelination model	Suppress cuprizone‐induced demyelination and motor dysfunction	MS	[411]

**TABLE 4:** List of the clinical trials of the drugs for NDDs by intervening iron or copper metabolism.

Disease	NCT identified	Trial phase	Intervention	Duration	Status	Study registration dates	Record last updates	Result	References
AD	NCT04149639	No specific data	Curcumin C3 complex	90–135 days	Completed	2019‐11‐08	2021‐03‐02	No significant difference in cognitive function was observed between the curcumin and placebo groups.	[412]
	NCT01716637	I	Etanercept	16 weeks	Completed	2010‐02	2016‐05‐12	Etanercept was well‐tolerated and demonstrated signals of potential benefit in cognitive, functional, and behavioral measures.	[412]
	NCT03234686	II	DFP	12 months	Completed	2018‐01‐19	2024‐07‐03	DFP treatment was associated with a reduction in hippocampal but also with an acceleration of cognitive decline.	[413]
PD	NCT00943748	II–III	DFP	6 and 12 months	Completed	2009‐10	2012‐09‐03	DFP reduced pathological iron accumulation, potentially improved motor function and delayed disease progression.	[370]
	NCT01539837	II	DFP	6 months	Completed	2012‐02	2020‐06‐16	DFP treatment was well‐tolerated and significantly reduced iron content.	[414]
	NCT02655315	II	DFP	9 months	Completed	2016‐02‐09	2022‐11‐07	In early PD patients not on levodopa, DFP worsened Parkinsonism scores compared to placebo.	[415]
AD	NCT00471211	II	PBT2	12 weeks	Completed	2007‐05‐08	2008‐01	The safety profile was favorable; PBT2 250 mg induced a significant, dose‐dependent reduction of CSF Aβ_42_ concentrations, and improved the category fluency test.	[416, 417]
PD	NCT03204929	I	Cu^II^(atsm)	28–168 days	Completed	2017‐06‐28	2020‐03	No results posted	Clinicaltrials.gov
ALS	NCT02870634	I	Cu^II^(atsm)	28–168 days	Completed	2016‐08‐10	2020‐03	No results posted	Clinicaltrials.gov
	NCT04082832	II	Cu^II^(atsm)	24 weeks	Unknown status	2019‐09‐02	2019‐11	No results posted	Clinicaltrials.gov
WD	NCT00004339	III	TTM	8 weeks	Completed	1999‐10‐18	2006‐05‐09	Some risk of neurologic deterioration, anemia and/or leukopenia, and transaminase elevations	[418]
	NCT02273596	II	ALXN1840	24 weeks	Completed	2014‐10‐20	2021‐09	Good safety profile with reduced non‐Cp Cu and disease‐related disability	[419]
	NCT03403205	III	ALXN1840	48 weeks	Terminated	2017‐12‐19	2024‐06‐17	ALXN1840 was superior to standard; the most frequent adverse effect was alanine aminotransferase (ALT) increase	[420]
	NCT01472874	Not applicable	Trientine	1 year	Completed	2011‐11‐11	2020‐04	Some risk of anemia and initial neurologic deterioration	[418]
	NCT03299829	Not applicable	Trientine	1 year	Recruiting	2017‐09‐25	2020‐08	No results posted	Clinicaltrials.gov
	NCT01874028	I	Trientine	> 1 year	Completed	2013‐06‐06	2014‐09	No results posted	Clinicaltrials.gov
HD	NCT01590888	II	PBT2	26 weeks	Completed	2012‐04‐18	2016‐06	PBT2 was generally safe and well‐tolerated	[421]

### 3.1 Alzheimer's Disease

#### 3.1.1 The Introduction of AD

AD, the most common cause of age‐related dementia, is a progressive disease that worsens over time, eventually leading to serious disability and death [422]. The major risk factors for AD include age, genetic predisposition, and family history [423]. Despite over 200 years of research, the exact etiology and pathology remain elusive. While several drugs have been approved by the FDA, none have been able to halt the progression of AD. Current hypotheses regarding the pathogenesis of AD continue to focus on the accumulation of abnormal Aβ and phosphorylated tau, as well as neuronal degeneration.

The pathogenicity of Aβ varies with its length, with longer Aβ_42_ exhibiting greater hydrophobicity, aggregation tendency, and toxicity [424]. The current amyloid hypothesis does not focus on total Aβ levels, but rather on the ratio of Aβ_42_/Aβ_40_ [425]. Compared to the extracellular plaques, the soluble oligomers of intracellular Aβ are more detrimental and are considered the causative factor [425]. The toxicity of Aβ oligomers is largely dependent on their size and structural characteristics [426]. Although the exact mechanism remains elusive, emerging evidence suggests a possible link to the toxic redox reactions of their metal‐bound forms [427].

Although tau is not prone to aggregation, under certain conditions, it can aggregate into oligomers and filaments [428], eventually forming neurofibrillary tangles (NFTs). The presence of NFTs leads to neural and glial cell degeneration and contributes to the development of tauopathies [429]. Numerous studies have shown that tau possesses a prion‐like self‐propagating ability that allows its aggregates to diffuse trans‐synaptically to other brain regions, thereby inducing further spread and aggregation [430]. Consistent with this, *tau* knockout mice show delayed neuronal maturation and impaired synaptic plasticity [431].

#### 3.1.2 Iron Dyshomeostasis in AD

Excessive accumulation of iron within cells and amyloid plaques, resulting from dysregulated iron homeostasis in the brain, is a hallmark of AD pathology [432]. Synchrotron‐based X‐ray spectromicroscopy studies have confirmed the presence of chemically reduced, low‐oxidation‐state iron (Fe^0^) within AD plaques in vivo [433, 434]. Recent findings suggest that the interaction between ferritin and Aβ might play a key role in the formation of iron‐containing amyloid deposits, which leads to increased oxidative stress [435]. In addition, impaired iron mobilization, as reflected by significantly elevated ferritin levels in serum, has been suggested to be a potential marker for preclinical AD [436].

**FIGURE 4:**
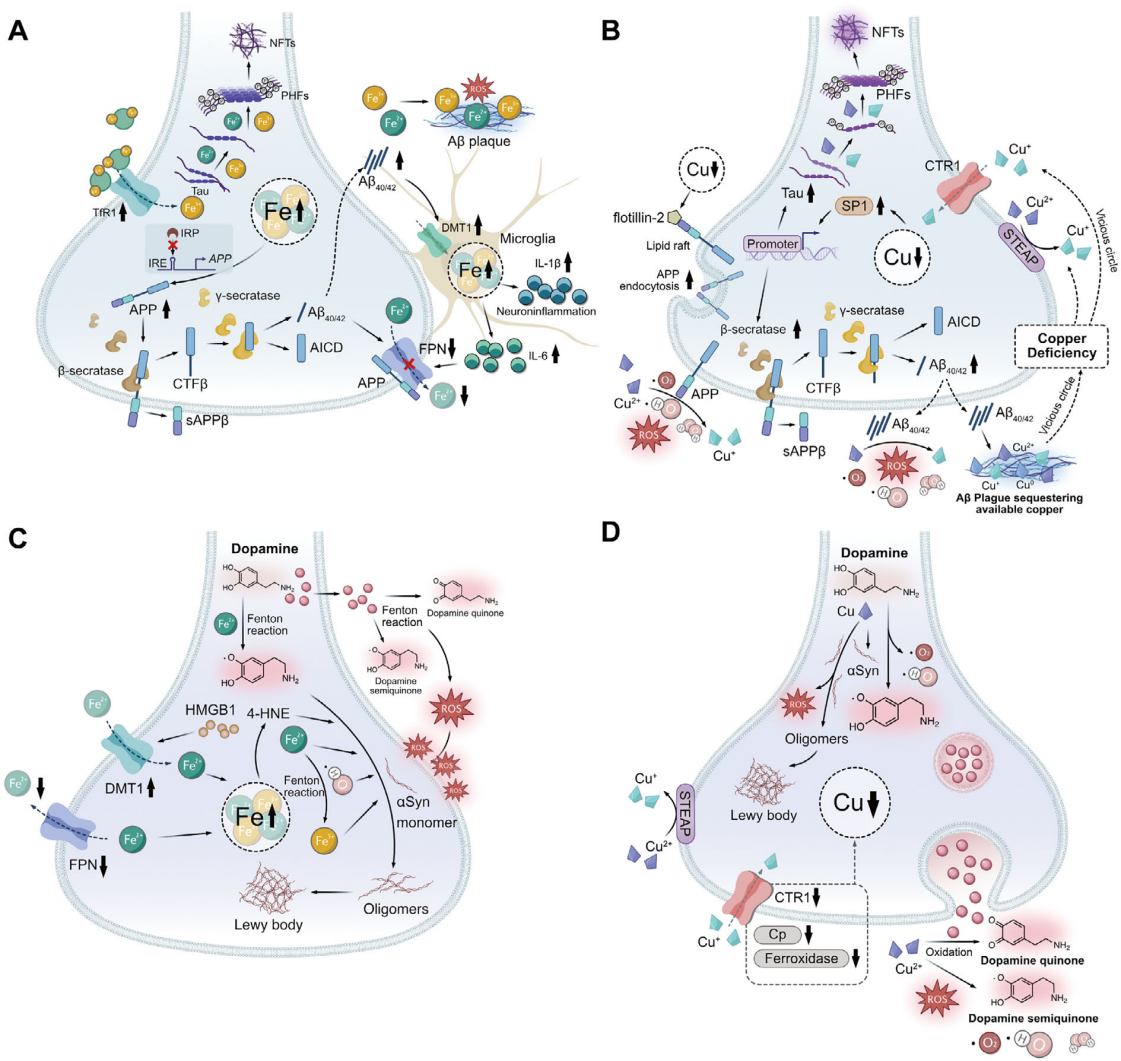
Iron and copper dyshomeostasis in AD and PD. (A) Elevated neuronal iron levels, resulting from upregulation of TfR1 and downregulation of FPN, increase APP transcription. Although APP stabilizes FPN, increased APP also promotes the generation of Aβ_40_/Aβ_42_, which destabilizes FPN and leads to intracellular iron retention. Aβ_40_/Aβ_42_ further enhances microglial iron uptake by upregulating DMT1, thereby exacerbating Aβ‐induced neuroinflammation and inflammatory cytokine secretion. Secreted IL‐6 promotes FPN degradation, worsening cerebral iron retention. Iron directly binds to tau, promoting its conformational change, phosphorylation, and aggregation. Within the core of Aβ plaques, Aβ fibers strongly chelate iron, causing local iron overload. This elevated iron concentration further catalyzes ROS production and promotes Aβ aggregation, exacerbating damage to surrounding neurons. (B) Copper levels are markedly decreased in the brain of AD patients. (1) After being reduced to Cu^+^ by STEAP, Cu^+^ is primarily transported into neurons by CTR1. Copper deficiency increases the expression of transcription factor SP1. SP1 promotes the transcription of *MAPT* (encoding tau) and *BACE1* (encoding β‐secretase). The binding of copper to tau can trigger hyperphosphorylation of tau and subsequent formation of NFTs. (2) Both Aβ and APP can reduce Cu^2+^ to Cu^+^. The interaction of Aβ or APP with copper generates ROS and superoxide radicals via the Haber–Weiss reaction. In addition, copper deficiency promotes the association of flotillin‐2 with cholesterol‐rich lipid rafts, thereby increasing Aβ synthesis by inhibiting APP endocytosis. (3) In the amyloidogenic pathway, APP is first processed by β‐secretase to produce sAPPβ and CTFβ. The latter is then processed by γ‐secretase to produce AICD and Aβ, especially the pathological Aβ_40_/Aβ_42_. Aβ_40_/Aβ_42_ is secreted into the extracellular space and then aggregated. Copper promoted the formation of Aβ plaque. Cu^0^, Cu^+^, and Cu^2+^ have been found in Aβ plaques.This could lead to further copper deficiency, creating a self‐perpetuating cycle. (C) Neuronal iron dyshomeostasis involves reduced FPN expression, which impairs iron export, while HMGB1 overproduction upregulates the iron importer DMT1. Together, these changes lead to excessive iron accumulation in neurons. Elevated iron directly promotes αSyn misfolding and aggregation. Hydroxyl radicals generated via the Fenton reaction oxidize αSyn. Dopamine‐derived quinones, catalyzed by iron, and lipid peroxidation products such as 4‐HNE from ferroptosis, covalently modify αSyn, further accelerating its misfolding and aggregation. These interconnected processes ultimately promote Lewy body formation. (D) PD patients show a significant copper deficiency in the SN. There is a marked decrease in neuronal CTR1, Cp, and ferroxidase activity. These changes contribute to copper deficiency. Copper accelerates the oxidation of dopamine, leading to the production of dopamine quinone, dopamine semiquinone, and the generation of superoxide radicals and ROS, which are toxic to neurons. Furthermore, under the influence of copper, αSyn is more prone to aggregation, leading to the formation of Lewy bodies.

In AD neurons, the expression of TfR1 is increased while that of FPN is downregulated, in correlation with elevated iron levels [437, 438] (Figure 4A). Iron influx or increasing iron level can enhance the translation of the *APP* mRNA transcript, which contains an IRE at the 5′ UTR region [44]. APP itself functions as a neuronal iron‐exporting chaperone by stabilizing FPN on the cell membrane [439], thereby assisting in the iron balance. However, the increase in APP also concurrently promotes its amyloidogenic processing into Aβ_40_ and Aβ_42_, which destabilize FPN on the cell surface, leading to intracellular iron retention [440]. Aβ also enhances microglial uptake of NTBI by upregulating DMT1 [441], and the resulting intracellular iron accumulation promotes a proinflammatory M1 phenotype, exacerbating Aβ‐induced IL‐1β secretion and neuroinflammation [442]. Chronic neuroinflammation further activates microglia, which release proinflammatory cytokines such as IL‐6. These cytokines upregulate hepcidin, which systemically inhibits iron recycling by promoting the degradation of FPN, leading to iron retention in the brain [283]. In addition, activated glial cells also accumulate iron and contribute to the transfer of excess iron to neurons [443].

Iron (Fe^2^^+^/Fe^3^^+^) can bind to Aβ, particularly Aβ_42_, via a high‐affinity site in its N‐terminus. This binding induces a conformational change in Aβ from a random coil to a β‐sheet‐rich structure, accelerating the formation of oligomers and fibrils [444]. In addition, iron catalyzes the Fenton reaction, which generates hydroxyl radicals that can oxidatively modify Aβ. Oxidized Aβ more readily forms stable oligomers, which then act as “seeds” to accelerate further aggregation [445]. Iron also catalyzes lipid peroxidation, producing aldehydes such as 4‐HNE and MDA. These products covalently modify Aβ, enhancing its conformational stability, aggregation propensity, membrane anchoring capability, and neurotoxicity [445]. Within the core of AD plaques, Aβ fibrils strongly chelate iron, leading to localized iron overload. This increased iron concentration further catalyzes ROS production and promotes Aβ aggregation, thereby intensifying damage to surrounding neurons [446].

Similar to Aβ plaques, NFTs in AD patients also frequently colocalize with high concentrations of iron [432]. Tau protein is both a regulator and a target of iron dyshomeostasis. On the one hand, tau promotes the trafficking of APP to the neuronal surface and assists in FPN‐mediated iron efflux [447]; on the other hand, tau deficiency can alleviate Aβ‐induced iron accumulation in the hippocampus [448]. Moreover, iron can directly bind to tau, promoting its conformational changes, phosphorylation, and aggregation. Iron‐catalyzed oxidative stress generates ROS that can oxidatively modify tau [449]. Oxidized tau is more susceptible to phosphorylation by kinases and acts as a “seed” to accelerate NFTs formation [450, 451]. Concurrently, ROS compromise microtubule stability, further amplifying tau detachment and aggregation [432]. Furthermore, iron overload activates tau kinases, such as glycogen synthase kinase‐3 beta (GSK‐3β), while inhibiting phosphatases like protein phosphatase 2A (PP2A), resulting in a net increase in tau phosphorylation [452].

#### 3.1.3 Copper Dyshomeostasis in AD

A large body of evidence suggests an imbalance in the regulation of metal ions, particularly copper, in the brains of AD patients [453]. In AD patients, brain copper levels are markedly decreased, whereas serum or plasma levels of copper and non–ceruloplasmin‐bound copper (non‐Cp Cu) are significantly increased, without significant changes in Cp levels [454].

How does copper contribute to the development of AD under conditions of copper deficiency in brain tissue? There are several possible mechanisms. (1) The expression of copper‐responsive transcription factor specificity protein 1 (SP1) is suppressed under conditions of copper sufficiency but is enhanced under conditions of copper deficiency [455]. SP1 is a key regulator in the transcriptional control of BACE1, which in turn modulates Aβ production through the processing of APP [456]. Besides, the promoter of the *tau* gene is also regulated by SP1 [457]. (2) Depletion of copper in cells promotes the shift of APP secretase cleavage from α‐secretase to β‐secretase activity, leading to an increase in Aβ production and the subsequent formation of plaque. The elevated Aβ levels can further contribute to localized copper deficiency in the neural system by sequestering available copper. This could escalate Aβ secretion, creating a self‐perpetuating cycle [458]. (3) Copper influences the association of flotillin‐2 with cholesterol‐rich lipid rafts, thereby reducing Aβ synthesis through the inhibition of APP endocytosis. Under conditions of intracellular copper deficiency, the formation of Aβ–copper complexes is more probable. This accounts for the observed hypermetallation of Aβ with copper in the presence of tissue copper deficiency in AD [459].

In addition to the effects of copper deficiency on AD, the redox cycling activity of copper has been implicated in the generation of ROS, which in turn, contributes significantly to the progression of AD [460]. The segment of APP, specifically residues 135–156, can convert Cu^2+^ into Cu^+^. This conversion entails an electron transfer process, which could potentially lead to an increased production of hydroxyl radicals [461]. Recent studies have revealed the presence of nanoscale metallic copper (Cu^0^) as well as Cu^+^ and Cu^2+^ in Aβ plaques in postmortem brain tissues from AD patients [462]. The interaction between copper and Aβ is widely regarded as the main driver of copper‐induced Aβ aggregation [266]. Specifically, the interaction between Aβ and Cu^2+^ facilitates the reduction of Cu^2+^ to Cu^+^ and simultaneously generates H_2_O_2_ via the Haber–Weiss reaction on extracellular Aβ [463]. Emerging evidence suggests that the chemical speciation of copper (Cu^2+^, or Cu^+^) may be of greater importance than its absolute concentration, as the redox‐active copper may promote oxidative stress [464].

Furthermore, the binding of copper to tau can trigger the hyperphosphorylation of tau and the subsequent formation of NFTs [266] (Figure 4B). The conserved MTBDs play a critical role in facilitating tau aggregation in vitro, leading to the generation of paired helical filaments (PHFs) and NFTs. Recent studies suggest that the neutralization of charges through modifications such as acetylation and ubiquitination on the MTBD is a critical step in the development of tau fibrils and the progression of AD [465]. In the presence of Cu^2+^, the R2 and R3 peptides adopt a monomeric α‐helical configuration, inducing the formation of PHFs and further aggregation leading to NFTs [209]. Furthermore, research has shown that the coordination of Cu^2+^ alters the electrostatic surface of the R1 peptide, thereby modulating tau aggregation in biological systems [210].

However, contrary findings have also been reported, suggesting that brain copper may potentially protect AD patients from cognitive decline and possibly slow the progression of cognitive decline [109]. For instance, studies have shown that low levels of copper in drinking water disrupt Aβ homeostasis in the brains of AD transgenic mice by affecting both Aβ production and clearance [466]. Collectively, these findings underscore the complex role of copper in the initiation and progression of AD, involving mechanisms related to oxidative stress, redox cycling of metal ions, and interactions with Aβ or tau [464].

### 3.2 Parkinson's Disease

#### 3.2.1 The Introduction of PD

PD, the second most prevalent NDD, affects approximately 6 million people worldwide. Its pathology is characterized by neuronal inclusions in the form of Lewy bodies and Lewy neurites with cell loss in the SN and other brain regions [467]. However, the etiology of PD remains elusive, and there is still no curative or preventive treatment available [468]. While the exact pathophysiology of PD remains unknown, oxidative stress and mitochondrial dysfunction are widely recognized as key factors in disease progression [469]. Autopsy evidence from PD brains reveals heightened oxidative stress affecting proteins, lipids, and DNA, along with decreased GSH levels and mitochondrial dysfunction [470]. Because aggregated and misfolded αSyn is a major component of Lewy bodies, PD is classified as a synucleinopathy. αSyn, a naturally unfolded protein, tends to aggregate into oligomers and fibrils under pathological conditions, adopting a toxic amyloid‐like conformation, particularly enriched in β‐sheet‐like structures [471]. It has been hypothesized that prion‐like cell‐to‐cell transmission and templating of synuclein are key mechanisms driving PD progression [472].

#### 3.2.2 Iron Dyshomeostasis in PD

In PD patients, the accumulation and deposition of iron in the putamen is generally increased, and its level is positively correlated with more severe motor symptoms and cognitive decline [473]. Different Parkinsonian syndromes exhibit characteristic distribution patterns of iron deposition in the putamen: in the Parkinsonian variant of multiple system atrophy, iron deposition is more concentrated in the dorsolateral putamen, whereas in progressive supranuclear palsy, it is more frequently observed in the anterior putamen [473].

Iron dysregulation is implicated in the loss of dopaminergic neurons in PD through a complex pathological cascade. This cascade begins with a disruption of neuronal iron homeostasis (Figure 4C). On the import side, there is an elevation of the expression of DMT1, which colocalizes with iron deposition in the SN of PD patients [474]. In response to increased iron levels, the upregulation of NEDD4 family‐interacting protein 1 (NDFIP1) protein provides a defense effect by promoting the ubiquitination and degradation of DMT1, thereby preventing iron entry and accumulation within neurons [475]. However, in cases of inflammation‐induced iron increase, the overproduction of high mobility group protein B1 (HMGB1) leads to an upregulation of DMT1 before quantifiable neuronal loss [476]. Additionally, a significant reduction of the expression of the principal iron exporter protein FPN has been observed in the AD brains, suggesting a severe impairment of iron efflux [477].

The aggregation of αSyn is a key pathological characteristic of PD. In PD models, the accumulation of iron has been shown to promote the transition of αSyn from a disordered monomer to a partially folded intermediate, thereby accelerating oligomerization and fibril formation [94]. Concurrently, iron overload also generates hydroxyl radicals via the Fenton reaction, resulting in the oxidation of αSyn and the formation of toxic oligomers, which act as “seeds” to accelerate further aggregation [478]. Besides, Fe^2^^+^ catalyzes the oxidation of dopamine via the Fenton reaction, leading to the rapid oxidation of dopamine into more toxic quinone species (e.g., dopaminochrome, aminochrome). These dopamine‐derived quinone products can covalently modify αSyn and promote its aggregation [479]. Lipid peroxidation products, such as 4‐HNE, generated during ferroptosis can also covalently modify αSyn, exacerbating its misfolding and aggregation [480]. Collectively, the resulting “iron deposition–αSyn aggregation” cycle continuously self‐amplifies, ultimately resulting in the formation of Lewy bodies and neuronal death [481].

#### 3.2.3 Copper Dyshomeostasis in PD

Copper imbalance is thought to be associated with the development of idiopathic PD. A recent cross‐sectional analysis indicated a nonlinear, dosage‐dependent correlation between copper consumption and the incidence of PD [482]. In healthy individuals, the SN region has the highest copper content in the brain [483]; however, PD patients show a copper deficiency in the SN [484]. Interestingly, PD patients show higher copper levels in the CSF when compared to healthy people [485, 486]. The pathological mechanisms linking copper deficiency in the PD brains to the onset and development of PD remain incompletely understood. Nonetheless, reports of the link between copper and PD pathology predominantly focus on the dopamine metabolism, oxidative stress, and αSyn aggregation [487] (Figure 4D).

A substantial reduction of neuronal CTR1 level has been observed in the SN region of PD patients [483]. In addition, the basal ganglia and SN regions from postmortem PD patients had higher iron levels and lower copper levels [112]. Given that copper is essential for the ferroxidase activity of Cp and plays a role in the maintenance of iron balance, insufficient copper levels may indirectly induce toxic effects by disrupting iron homeostasis through impaired ferroxidase activity of Cp [488]. Consequently, this could lead to disruptions in iron export from the brain and potentially result in the release of copper from the enzyme, thereby liberating free copper ions that could contribute to the generation of additional free radicals. In line with this, mice lacking Cp experienced neuronal cell death in the SN [489].

A significant amount of copper is detected in the neuromelanin of the SN, particularly within the locus coeruleus, suggesting that copper may play an active role in dopamine oxidation [490]. The interaction of copper ions with αSyn facilitates dopamine oxidation, leading to the production of potentially harmful substances, such as dopamine quinone and superoxide radicals [491]. Additionally, αSyn is known to form partially folded amyloid conformations that are more prone to aggregation in the presence of copper [471]. Notably, even at physiological concentrations, copper accelerates the formation of αSyn fibrils [492]. The aggregation of aSyn significantly boosts ROS production in the presence of Cu^2+^ [493].

### 3.3 Prion Diseases

#### 3.3.1 The Introduction of Prion Diseases

PrDs, known as transmissible spongiform encephalopathies, are a group of degenerative encephalopathies of the CNS that affect humans and a wide range of animals, with a long incubation period and a 100% lethality rate. Human PrD can be divided into disseminated, inherited, and acquired types, including Creutzfeldt–Jakob disease, Kuru disease, GSS syndrome, and so forth, of which the disseminated type of Creutzfeldt–Jakob disease accounts for 85%–90% [494]. Diagnosis can now be made by testing for abnormal PrP^Sc^ in many biological fluids and peripheral tissues such as CSF. In the absence of targeted therapies, treatment of PrD can only be supportive [495]. Neuropathological changes in PrD include spongy vacuolization, astrocyte and microglia activity, neuronal loss, and PrP^Sc^ deposition. In mice, the activation of microglia and the occurrence of astrocytosis are observed to occur before the death of neurons [496]. PrP^Sc^ in aggregated form accumulates in the CNS and is associated with characteristic neuropathological changes in the cerebral and cerebellar cortices, as well as in the deep brain nuclei [494]. The exact mechanism of prion transmission has not been clarified. The monomers are added to the ends of the PrP aggregate and spread by fragmentation of the prion fibers [497]. The presence of PrP^Sc^ is crucial for the onset and spread of PrDs, as well as for the disease's severity [498].

#### 3.3.2 Iron Dyshomeostasis in Prion Diseases

Growing evidence indicates that PrP^C^ is critically involved in regulating brain iron homeostasis. This is supported by the key observations that altered iron metabolism and reduced iron levels have been found in the brains of *PRNP* (which encodes PrP^C^ protein) knockout mice [236], and altered levels of the iron‐related proteins ferroxidase and Tf have been reported in the CSF of patients with Creutzfeldt–Jakob disease [237].

Mechanistically, PrP^C^ functions as a ferrireductase and a modulator of cellular iron uptake, an activity dependent on its octapeptide repeat domain and its association with the plasma membrane [499]. PrP^C^ promotes the conversion of Fe^3^^+^ to Fe^2^^+^, and the reduced, divalent iron is subsequently transported across the membrane via the ZIP14/DMT1 complex [95]. Moreover, the mRNA of the *PRNP* gene contains an IRE, making its expression subject to modulation by iron levels [238]. Therefore, this establishes a precise regulatory circuit where PrP^C^ controls iron uptake, while its own production is, in turn, regulated by iron status.

#### 3.3.3 Copper Dyshomeostasis in Prion Diseases

Studies reported that copper levels in the brains of PrD patients are 50% lower compared to healthy controls [500]. But the role of copper in PrD remains contentious. While copper is essential for maintaining the “normal” and noninfectious state of PrP^C^, its deficiency can lead to PrD development. When bound to copper, PrP^C^ exhibits SOD1‐like activity, wherein copper acts as an antioxidant, thereby increasing the survival of neurons [501]. Copper also prevents the connection between PrP^C^ and PrP^Sc^ by triggering the internalization of PrP^C^ from the cell's exterior, thus diminishing the transfer of PrD [502]. Furthermore, PrP^C^ and copper can jointly inhibit the activation of NMDA receptors, thereby protecting neurons from excitotoxicity [503]. Studies have shown that copper ions delay the onset of PrD in model mice and significantly reduce PrP^Sc^ accumulation in the infected neuroblastoma cells [504]. It is also reported that copper chelators induce clinical signs resembling pruritus in mice. Thus, copper may play a protective role in PrD.

However, there is another perspective that the binding of PrP^C^ to copper can promote its conformational change to PrP^Sc^, enhancing protease resistance and protein infectivity [114]. Treatment with the copper chelator D‐penicillamine (DPA) reduced copper levels in the blood and brain and delayed the onset of PrD in intraperitoneal scrapie inoculation mice [505]. In addition, Cu^2+^ is associated with neurotoxicity mainly through its binding to His‐111, Met‐109, and Met‐112 of human PrP^C^, which are involved in its aggregation [506]. These findings suggest that copper may promote the onset and development of PrD. In summary, copper appears to have a contradictory function in the development of PrD, with the exact mode of action still being enigmatic [507].

### 3.4 Huntington's Disease

#### 3.4.1 The Introduction of HD

HD is a rare, autosomal dominant NDDs characterized by the progressive decline of motor, cognitive, and psychiatric functions. The genetic cause of HD is an aberrant expansion (> 35) of the CAG repeat sequence in exon 1 of the *HTT* gene. The CAG repeats encoded polyQ further lead to the misfolding and abnormal aggregation of the mHTT protein and its toxicity. The presence of mHTT results in a gradual deterioration of neuronal function. Consequently, noticeable shrinkage of the basal ganglia nuclei and the subcortical white matter is observed before the emergence of motor symptoms. As the disease progresses, there is also a significant loss of neurons in the cortical, thalamic, and hypothalamic regions, eventually throughout the brain [508]. The underlying pathogenesis of HD is complex and wide‐ranging, including dysregulation of the ubiquitin/proteasome and autophagy systems, leading to the accumulation of toxic proteins [509]. Both oxidative stress and inflammation in the periphery and CNS are present at the outset of the disease [510]. Moreover, the mHTT impacts the regulation of gene expression and the transport along axons, leading to reduced BDNF‐mediated signaling [511]. In addition, in HD patients, there is a disruption in cortical glutamate signaling and striatal dopamine signaling, indicating issues with synaptic function [511]. The pathophysiology of HD is very complex, and correcting this is not that simple [512]. The pathogenic process evolves very slowly, over a period of more than 40 years, making both detection and intervention particularly difficult [511].

#### 3.4.2 Iron Dyshomeostasis in HD

HD is characterized by dynamic and region‐specific alterations in brain iron metabolism. Neuroimaging studies reveal a temporal progression: the premanifest HD stage shows iron accumulation in subcortical structures like the putamen and globus pallidus, suggesting that iron deposition in subcortical structures could serve as an early biomarker of HD [513, 514]. As the disease advances to early HD, quantitative susceptibility mapping confirms iron accumulation in the basal ganglia, with the degree of iron deposition in the putamen and caudate nucleus positively correlating with disease severity [515]. Interestingly, iron levels show a complex relationship with brain structure, exhibiting negative correlations with the volumes of the putamen, globus pallidus, and anterior cingulate cortex, but positive correlations with some cortical structure volumes [516]. Longitudinal data further establish that the rate of iron deposition in the caudate nucleus and globus pallidus can serve as a dynamic indicator for monitoring disease progression [517]. Postmortem studies further confirm iron deposition in striatal astrocytes of HD patients [518].

Although early studies reported decreased serum ferritin levels in HD patients [519], transgenic HD models (N171‐82Q, R6/2, YAC128) consistently demonstrate elevated iron levels in the striatum and cortex, accompanied by upregulation of iron metabolism proteins like Tf, ferritin, and TfR [520, 521]. Furthermore, neonatal iron loading exacerbates oxidative stress, energy dysfunction, and striatal degeneration in these models, highlighting the toxicity of iron in the vulnerable HD brain [522, 523].

Notably, elevated ferritin in microglia represents an early event in HD [524]. Iron accumulation has been reported to activate microglia and promote the kynurenine pathway metabolism [96]. At the molecular level, HD model mice show mitochondrial iron accumulation, linked to upregulated MFRN2, downregulated frataxin, and exacerbated lipid peroxidation [97]. Mechanistically, the nuclear translocation of STAT5 elevates the expression of cytoplasmic aconitate hydratase (ACO1), which is also known as iron regulatory protein 1 (IRP1), an IRP regulating the expression of genes involved in iron metabolism, ultimately driving iron deposition [515]. APP also participates in the regulation of iron metabolism in HD, as its knockdown increases cerebral iron levels in YAC128 mice [525].

Most importantly, a central regulatory role has been identified for the HTT protein. HTT has been characterized as an iron‐regulated protein, and its deletion disrupts brain iron homeostasis [526]. This finding places HTT at the center of the pathological iron dysregulation in HD. Targeting this disrupted iron homeostasis pathway, therefore, represents a promising therapeutic strategy for mitigating disease progression.

#### 3.4.3 Copper Dyshomeostasis in HD

In HD patients, copper concentrations in the brain are notably elevated, with significant increases observed in the thalamus (64%) and ribosomes (68%) [527]. However, there are also studies suggesting that copper levels in the brains of HD patients remain unchanged or even decreased [117]. Research has shown that the copper transporter 1B (Ctr1B) and *Dm*ATP7 participate in maintaining copper balance within the brain of *Drosophila melanogaster*. In a *Drosophila* model of HD, it was discovered that changes in the levels of Ctr1B or *Dm*ATP7 in the brain, or adjustments to the copper content in their food, had a significant impact on the symptoms associated with the expression of Htt exon1‐polyQ. Substitution of Htt Met8 and His82, two potential copper‐binding residues, could eliminate the modulatory effect of copper on Htt exon1‐polyQ toxicity. Thus, HD might not solely be a polyQ disorder but could also be influenced by copper levels [528].

Reports indicate that copper in vitro can markedly enhance the formation of fibrils and aggregates from the purified recombinant human proteins containing exon1 with polyQ sequences [529]. Furthermore, the segment encompassing the first 171 amino acids of the human wild‐type HTT protein and its mutant variant with expanded glutamine repeats has been shown to interact directly with copper [252]. In addition, lactate dehydrogenase (LDH), a crucial enzyme in regulating lactate levels and providing energy substrates to neurons, is inhibited by copper, which also contributes to the neurodegeneration in HD [507]. In addition, elevated levels of MT‐3 in mammalian cells substantially decreased the clumping and toxicity associated with polyQ proteins, suggesting CuBPs such as MT proteins may be potential targets for the treatment of HD [529].

### 3.5 Wilson's Disease

#### 3.5.1 The Introduction of WD

WD is a hereditary condition inherited in an autosomal recessive pattern caused by mutations in the *ATP7B* gene. Historically known as hepatolenticular degeneration, it typically manifests in adolescence or early adulthood with symptoms including liver problems and movement disorders. Studies conducted in East Asia have indicated a significantly higher occurrence of WD, with estimates ranging from 1/1500 to 1/3000. It is suggested that the incidence of WD may be much higher in geographically or culturally isolated populations [530]. A significant proportion of individuals, up to 60%, initially experience neurological or psychiatric issues, known as the neurological presentation of the disease, regardless of liver condition [531]. On the other hand, patients who first display liver‐related symptoms, from abnormal liver function tests to hepatomegaly, splenomegaly, or acute liver failure, are categorized as having hepatic presentations [532].

Over 500 different mutations in the *ATP7B* gene have been discovered, with certain mutation hotspots showing varying prevalence across ethnic groups. Mutations leading to a complete loss‐of‐function or absence of the ATP7B protein typically result in a severe form of WD that manifests early, often affecting the liver, although such mutations are relatively uncommon [533]. There is a proposed association between specific point mutations, such as 3207C → A, and the delayed onset of neurological symptoms in WD, although this connection has not been definitively established across multiple independent studies [534].

#### 3.5.2 Iron Dyshomeostasis in WD

The pathophysiology of WD involves a critical disruption in iron metabolism stemming from impaired Cp function or reduced serum Cp levels [535]. In WD, mutations in the *ATP7B* gene prevent the incorporation of copper into Cp, resulting in a lack of the copper cofactor essential for its ferroxidase activity [536]. Biochemically, WD resembles an acquired form of aceruloplasminemia (ACP), a rare autosomal recessive disorder caused by *Cp* gene mutations [537]. In ACP, loss of Cp ferroxidase activity disrupts iron mobilization, causing systemic overload. Diabetes typically appears around age 38.5, while neurological symptoms from brain iron deposition emerge near age 50 [538]. Thus, despite WD's primary copper defect, it shares ACP's core pathophysiology of impaired iron handling, leading to reduced circulating iron, increased storage, and pathological accumulation in organs like the liver [536]. Accumulated iron catalyzes the production of ROS via the Haber–Weiss and Fenton reactions, leading to oxidative stress and cellular damage [260].

#### 3.5.3 Copper Dyshomeostasis in WD

Mutations in the *ATP7B* gene result in a dysfunctional protein with a shortened half‐life, abnormal conformation, and aberrant intracellular localization. The mutant ATP7B protein also exhibits impaired interaction with ATOX1, thereby hindering copper transport [539]. As a result, copper accumulates excessively in hepatocytes [137]. As the intracellular copper levels exceed the capacity of MT/GSH, hepatocytes undergo intense oxidative stress, leading to severe cellular damage [540]. Upon hepatocyte death, unbound copper is released into the bloodstream, posing a significant toxicity risk. Free copper, not bound to Cp, is highly toxic and can be deposited in organs such as the kidney and brain [285]. Copper ions that cross the BBB into the peripapillary space are captured by astrocytes. Neuronal damage in WD is considered secondary and nonspecific, attributed to astrocyte dysfunction and the toxic effects of copper ions released from damaged astrocytes. The resulting gliosis serves as the primary morphological basis for the neurological syndrome associated with WD [285].

Traditional WD pathology suggests that *ATP7B* mutations cause impaired copper efflux from cells, which results in copper spillage into the CSF and causes oxidative damage to the CNS [84]. However, other pathological theories suggest that ATP7B disorders in astrocytes may lead to the production of impaired Cp and thus Fe efflux, similar to hereditary Cp deficiency [541]. Recently, Li et al. discovered that Zn transporter 1 (ZnT1) also plays a role in copper import into cells [542]. They revealed that Cu^2+^ has the same primary binding site as Zn^2+^ in ZnT1, with Zn^2+^ competing for ZnT1‐mediated Cu^2+^ uptake. Interestingly, they also found that ZnT1 may be a potential target for the Zn‐mediated treatment for WD [542].

### 3.6 Menkes Disease

#### 3.6.1 The Introduction of MD

MD, also known as curly hair syndrome, is an inherited disorder arising from mutations in the *ATP7A* gene, which follows an X‐linked recessive inheritance pattern. Infants with MD are typically born with no apparent symptoms but exhibit severe neurological deterioration between the ages of 2–4 months, often resulting in mortality before the age of 3 years [84]. The incidence of MD is reported to be about 1/300,000 in most countries [543]. Males are predominantly affected, with only a few cases in females, possibly due to XO/XX mosaicism or abnormal X chromosome inactivation [544].

The *ATP7A* gene is the only known gene associated with MD, with mutations identified in approximately 95% of children with clinically diagnosed MD. There are 155 *ATP7A* mutations reported in the human mutation database [545]. These mutations are genetically diverse, and the same mutation within a family can present with different clinical phenotypes [546]. Research on the functional implications of *ATP7A* mutations is still limited [547].

#### 3.6.2 Iron Dyshomeostasis in MD

In MD, defective copper metabolism reduces the antioxidant capacity of red blood cells, leading to hemolysis. Hemolysis induces an increase in hepatic hepcidin, which inhibits iron recycling, resulting in disrupted iron metabolism and exacerbating anemia. As a result, the pathological conditions of MD can be characterized by a systemic disorder of iron homeostasis triggered by copper deficiency, with hemolysis being a central feature [548]. This copper‐deficient state affects the proper function of several key copper‐dependent enzymes, subsequently disrupting iron metabolism in MD. Notably, the synthesis and functionality of Cp are affected. Due to the lack of copper, the liver produces only the inactive copper‐free apo‐Cp instead of the functional holo‐enzyme, resulting in a severe loss of its ferroxidase activity. Consequently, Fe^2^^+^ released in tissues cannot be oxidized to Fe^3^^+^, which hinders the loading of iron onto Tf, and leads to decreased serum iron levels, thereby inducing a state of “functional iron deficiency” that clinically manifests as microcytic hypochromic anemia [549]. Furthermore, copper deficiency also impairs the function of another critical copper‐dependent enzyme, HEPH. Within intestinal cells, impaired HEPH activity compromises the release of dietary iron from intestinal cells into the bloodstream, thereby exacerbating systemic iron insufficiency [550]. Together, the dysfunctions of Cp and HEPH create a vicious cycle of interconnected copper and iron metabolic disturbances that characterize the pathophysiology of MD.

#### 3.6.3 Copper Dyshomeostasis in MD

Mutations in the *ATP7A* gene can reduce or abolish the expression and function of the ATP7A protein, disrupting copper transport by gastrointestinal mucosal cells. This results in decreased copper levels in both plasma and the brain, while paradoxically causing copper accumulation in tissues such as the duodenum, kidney, spleen, pancreas, skeletal muscle, and placenta [84]. The dysregulated copper balance leads to decreased or lost activity of essential copper‐dependent enzymes, which ultimately triggers the development of a range of clinical manifestations in MD patients [285]. Recent studies suggested that the mutated *ATP7A* gene product exists in both truncated and elongated forms, with the truncated form being present in the ER and being responsible for the development of mild MD [551].

### 3.7 Amyotrophic Lateral Sclerosis

#### 3.7.1 The Introduction of ALS

ALS is a progressive and painless muscle weakness resulting from the degeneration of motor neurons throughout the brain, brainstem, and spinal cord [552]. The global incidence of ALS is approximately 1/50,000, with an estimated 1.5 million patients worldwide [553]. Due to an aging population, the number of people with ALS is increasing exponentially, and by 2040, there will be approximately 400,000 people with ALS worldwide [554].

ALS is now understood as a systemic disease with a widely varying clinical presentation, including nonmotor symptoms, behavioral changes, and cognitive decline, including frontotemporal dementia. Patients with ALS typically die of respiratory failure within two to 4 years of diagnosis, although a small proportion of patients progress more slowly [555]. Approximately 90% of ALS cases are sporadic, a tenth of the individuals with ALS have a hereditary pattern of the illness, and to date, over 30 genes linked to ALS have been discovered [556]. The causative mutations found in familial ALS cases have also been described in many sporadic cases [557]. Pathogenic variants in the most common ALS‐associated genes, including SOD1, TDP43 (TAR DNA‐binding protein 43), FUS (RNA‐binding protein FUS), and C9orf72 (chromosome 9 open reading frame 72), constitute roughly 60% of the inherited cases and around 10% of the noninherited ALS instances [558].

The most common cause of ALS is mutations in the *SOD1* gene. Currently, more than 180 cases of *SOD1* mutations have been reported. Due to the high prevalence of *SOD1* mutations, which account for 20%–25% of familial ALS cases, the *SOD1* gene is considered to be one of the main targets for ALS therapeutic regimens [559].

#### 3.7.2 Iron Dyshomeostasis in ALS

In ALS, disrupted iron metabolism leads to abnormal iron accumulation in the brain, especially within the corticospinal motor pathways [560]. In ALS tissues, pathological relevance is evidenced by Tf deposition in Bunina bodies and basophilic inclusions [561]. Furthermore, blood–spinal cord barrier disruption contributes to the accumulation of blood‐derived iron and neurotoxic hemoglobin in the spinal cord, thereby accelerating early motor neuron degeneration [562].

In SH‐SY5Y cells overexpressing SOD1^G93A^, elevated mRNA levels of iron importers, and increased expression of key mitochondrial iron handling proteins (including MFRN1/2, frataxin, and Fe–S cluster) were observed, suggesting that SOD1^G93A^ contributes to a disruption of iron homeostasis [563]. In SOD1^G93A^ ALS mouse models, iron accumulates abnormally in motor neurons and glial cells, with elevated DMT1, FPN, and Cp levels in the cervical spinal cord, along with elevated ferritin content in glial cells [564].

Mechanistically, SOD1^G93A^ disrupts iron homeostasis by impairing the Akt/FOXO3a signaling pathway, which upregulates iron import while downregulating iron export proteins [565]. This leads to further intracellular iron accumulation and promotes ROS production, creating a vicious cycle [565]. SOD1^G93A^ is also likely to impair Fe–S cluster assembly [566]. The resulting iron accumulation activates a metalloprotease ADAM17 (disintegrin and metalloproteinase domain‐containing protein 17), promoting TNF‐α secretion and inducing oxidative stress [98].

The consequences of this dysregulation extend to ferroptosis. Investigations across multiple ALS transgenic models, including SOD1^G93A^, TDP43^Q331K^, and C9orf72^500^, have revealed GPX4 depletion and ferroptosis in both spinal cord and brain tissues, which are closely associated with impaired Nrf2 signaling and downregulation of Fth [99]. The decreased activity of GPX4 impairs the cell's ability to clear lipid peroxides, thereby enhancing susceptibility to ferroptosis [567]. Both GPX4 overexpression and Nrf2 activation have been shown to ameliorate motor dysfunction and delay neurodegeneration in SOD1^G93A^ mice [99]. Additionally, ALS mice and cells displayed significantly lower expression of a novel ferroptosis suppressor, speedy protein A (SPDYA); the overexpression of SPDYA inhibits ferroptosis by modulating the GCH1/BH4 axis and TfR1, thereby significantly extending the survival of SOD1^G93A^ mice [100].

#### 3.7.3 Copper Dyshomeostasis in ALS

The activity and stability of SOD1 are reliant on PTMs, including copper binding. Cu binding enhances the thermal stability of SOD1 and enables its enzymatic activity. In the absence of PTMs, SOD1 becomes thermally unstable and prone to form multimers or high‐energy conformations [568].

In humans, CCS stands as the only copper chaperonin known to interact directly with and activate SOD1. It facilitates the folding and maturation of SOD1 into its active form. This process involves an ATP‐independent mechanism, wherein CCS functional structural domains I and III are brought into the immediate vicinity of nascent SOD1. CCS forms a heterodimeric complex with SOD1, which is crucial for the proper folding of the enzyme [569].

Mutations or misfolding of SOD1 have been associated with the development of ALS. These mutations are typically located at the conserved MBSs, interfaces, and β‐barrel structural domains, each of which hinders the likelihood that SOD1 will progress along the maturation pathway. Misfolded SOD1 has been found in both neurons and glial cells across regions such as the spinal cord, brainstem, and hippocampus areas of ALS patients, highlighting its potential role in the pathogenesis of ALS [570]. These pathological aggregates in these regions not only contain SOD1, but also incorporate heat shock protein, ubiquitin, proteasome, and CCS, indicating a complex interplay of cellular components in ALS pathology [571].

### 3.8 Multiple Sclerosis

#### 3.8.1 The Introduction of MS

MS is a condition where the body's immune system mistakenly attacks the CNS, leading to inflammation and damage to the protective covering of nerve fibers, known as demyelination, and the severing of axons, which are the nerve fibers themselves. This axonal damage is a sign of permanent neurological harm [572]. The hallmark of MS is the presence of localized demyelination and inflammatory patches, predominantly in the white matter of the brain, which can be detected using MRI. However, lesions in the gray matter and the cerebral cortex, while frequently observed in pathological examinations, are often not easily discernible with the current diagnostic imaging techniques [573]. Following the initial phase of inflammation in MS, the affected areas may transition to a chronic phase characterized by several possible outcomes: remyelination, the subsidence of inflammation without tissue repair, or a persistent state where inflammation and the breakdown of myelin continue concurrently. Both inflammation and the process of neurodegeneration are observed to varying extents in MS patients at the onset of the disease [574]. Moreover, the balance and severity of these two processes can fluctuate within the same individual as the disease progresses over time [575].

Diagnoses of MS are most commonly made in individuals between the ages of 20 and 30, and the condition can have a profound impact on physical capabilities, mental processes, overall well‐being, and work capacity. The exact origins of MS remain a mystery, although a variety of genetic predispositions and environmental factors are believed to play a role in its development [576].

#### 3.8.2 Iron Dyshomeostasis in MS

In MS, dysregulation of iron metabolism in the CNS is a key driver of disease progression. Clinical evidence from cross‐sectional studies reveals that iron accumulation in deep gray matter progressively increases from relapsing–remitting to secondary progressive MS, peaking in primary progressive MS [577]. Autopsy studies confirm that the areas of iron deposition closely overlap with regions of neurodegeneration and demyelination [578]. A particularly significant radiological finding is the presence of iron rim lesions; clinical studies indicate that the presence of ≥ 4 such lesions predicts earlier onset of motor and cognitive impairments, poorer long‐term outcomes, and correlates with higher clinical disability scores [579, 580].

The pathological iron originates from several specific cellular sources. Oligodendrocytes, which possess the highest intracellular iron content in the CNS, release iron into the extracellular space upon their damage and demyelination [581, 582, 583]. Furthermore, monocyte‐derived macrophages infiltrating the CNS also contribute to cerebral iron accumulation by taking up iron complexes.

The resulting iron metabolism dysregulation triggers multiple pathological mechanisms. In the gray matter of MS patients and the spinal cords of experimental autoimmune encephalomyelitis (EAE) mice, the expression of the key ferroptosis inhibitor GPX4 is downregulated [584]. Ferroptosis is not only an early event in EAE [585] but also plays a role in various demyelination models [586]. Iron imbalance also disrupts calcium/glutamate signaling, leading to excitotoxicity, cellular swelling, and activation of proteolytic enzymes that accelerate neuronal degeneration [101]. A vicious cycle forms where iron overload and glutamate release exacerbate postsynaptic calcium overload, mitochondrial dysfunction, and iron deposition, ultimately resulting in neuronal death [587]. Iron imbalance also affects energy metabolism, in which iron‐dependent ROS not only induce mitochondrial DNA loss [102], but also directly impair respiratory chain function [588]. Additionally, in vitro studies suggested that ferrous ions can exert neurotoxicity by activating sphingolipid metabolism and promoting pathological protein aggregation [589, 590, 591].

#### 3.8.3 Copper Dyshomeostasis in MS

Notably, MS patients exhibit significantly elevated copper levels in both serum and CSF compared to healthy controls. This increase may be attributed to the decreased serum Cp activity, which affects copper absorption [124]. Moreover, upregulation of copper transporters in the CNS and BBB impairment at active MS lesions may both contribute to increased CNS copper levels. Once inside, copper uptake and release by astrocytes are facilitated, ultimately contributing to demyelination [123].

The proposed role of copper dyshomeostasis in the pathogenesis of MS is theorized to stem from an excessive accumulation of copper, which triggers heightened oxidative damage [592]. The elevated copper levels within the CNS have been linked to impairments in the mitochondrial electron transport system, including COX, along with the activation of glial cells. However, various studies have yielded some inconsistent findings reported in this regard [593]. The copper chelator cuprizone is a well‐established animal model for studying CNS demyelination and remyelination. It supports the link between copper imbalance and ROS production by facilitating copper entry into the CNS and inducing demyelination through oligodendrocyte toxicity and oxidative stress [594, 595].

## 4 The Therapies for Regulating Iron and Copper Metabolism in the Treatment of NDDs

Over the years, many agents and therapeutic strategies have been developed to target cellular iron and copper metabolism (Table 3). Several iron/copper‐based or iron/copper‐binding compounds have demonstrated potential for the treatment of NDDs. Iron/copper‐binding compounds are primarily divided into two classes: ionophores, which transport ions across cell membranes, and chelators, which bind ions into stable ring structures to regulate cellular levels. Additional functional agents that modulate iron and copper metabolism include metal–protein attenuating compounds (MPACs), iron/copper–protein binding agents, and natural products (Table 3).

### 4.1 Therapeutics Targeting Iron Homeostasis

Therapeutic strategies targeting iron dyshomeostasis have shown promise in several NDDs, with approaches ranging from classic chelators to multifunctional natural compounds (Table 3).

#### 4.1.1 Iron Chelator

In P301L tau and APP/PS1 mouse models, intranasal deferoxamine (DFO) suppresses ferroptosis, lowers oxidative stress, and mitigates cognitive decline [366]. DFO enhances dopaminergic neuron survival and suppresses αSyn aggregation [367]. Deferiprone (DFP) is an orally bioavailable iron chelator approved for treating systemic iron overload disorders [596]. DFP therapy exerts a protective effect by reducing brain iron deposition, improving tau and αSyn pathology, and mitigating iron‐mediated neurotoxicity [368, 369, 370]. The lipophilic metal chelator, 8‐hydroxyquinoline (8‐HQ), also demonstrates anti‐AD potential in AD models. The novel iron chelator PBT434, a quinazolinone compound bearing a moderate affinity metal‐binding motif, modulates iron uptake in human BMVECs [371]. PBT434 also inhibits iron‐mediated oxidative stress and lowers nigral αSyn aggregation in several different PD mouse models, thereby improving the survival of dopaminergic neurons in the SNpc of mice [372]. In tau^−/−^ mice, a model of Parkinsonism, PBT434 not only alleviates olfactory deficits and bulbar iron accumulation in young mice but also reduces motor impairments and nigral degeneration in aged mice [597].

#### 4.1.2 Multifunctional Iron Chelators

Multifunctional iron chelators are a class of compounds that not only can bind and remove excess iron ions but also possess additional pharmacological activities, such as antioxidant, anti‐inflammatory, inhibition of protein aggregation, regulation of metal homeostasis, and mitochondrial protection [598]. Derivatives of 8‐HQ, such as M30 and VK‐28, possess both iron‐chelating and antioxidant activities, which can reduce the formation of ROS, thereby protecting neurons from oxidative damage [599]. The multifunctional iron chelator M30 increases striatal dopamine levels and enhances dopaminergic neuron populations and TfR‐positive cells in the SNpc [373]. Meanwhile, motor neuron degeneration can be prevented by the brain‐permeable iron chelators M30 and VK‐28 in SOD1^G93A^ mouse models of ALS [374]. VAR10303, a brain‐permeable iron chelator, significantly ameliorates PD symptoms in both the 6‐OHDA‐induced rat model and the MPTP‐treated mouse model [375]. In the MPTP‐induced PD mouse model, the PPARβ/δ agonist GW501516 modulates cellular iron homeostasis through the regulation of FPN and ferritin expression, without affecting TfR levels, and demonstrates the potential to alleviate motor deficits and reduce NLRP3 inflammasome‐mediated neuroinflammation [376].

#### 4.1.3 Natural Compounds

Natural compounds constitute a precious heritage of human culture. Especially relevant to the modulation of iron, several natural products have demonstrated potential in alleviating NDDs through antiferroptosis mechanisms. In AD‐related models, curcumin binds Fe^2^^+^, reduces amyloid and oxidized protein aggregation, and improves cognitive deficits [377]. Eriodictyol inhibits tau phosphorylation in Aβ_42_‐induced HT22 cells, and activates the Nrf2/HMOX1 pathway to combat ferroptosis, ameliorating cognitive impairment in APP/PS1 mice [378]. The natural flavonoids sterubin and fisetin strongly inhibit ferroptosis in HT22 neuronal cells [379]. Berberine demonstrates neuroprotective value by modulating iron levels, inhibiting ferroptosis, and activating the Nrf2 pathway in 3 × Tg AD mice [380]. Naringin effectively reduces nonheme iron and decreases amyloid plaque formation in iron‐overloaded mice [381].

In PD‐related models, DL‐3‐n‐butylphthalide, isolated from celery seeds, reduces motor deficits, dopaminergic neuron loss, iron deposition in the SN, and serum iron levels in rotenone‐treated rats [382]. Quercetin exerts antiferroptosis effects via Nrf2 activation in MPP^+^‐treated cellular models and in MPTP‐induced mouse models [383]. Epigallocatechin gallate (EGCG) inhibits Fe^3^^+^‐induced αSyn conformational changes and protects PC12 cells from Fe^3^^+^ toxicity [384] (Table 3).

#### 4.1.4 Relevant Clinical Trials

Clinical investigations into iron chelation therapies have yielded disease‐specific findings with varying levels of efficacy and safety considerations (Table 4).

Several clinical trials (ClinicalTrials.gov identifiers: NCT04149639, NCT01716637, etc.) have investigated the efficacy of several different agents for treating AD. A 24‐week placebo‐controlled trial on curcumin C3 Complex did not observe notable cognitive function differences between the curcumin and placebo groups [412]. Additionally, a Phase II clinical trial (NCT03234686) was carried out at nine sites in Australia to investigate the effect of DFP on AD, which incorporated neuropsychological tests and MRI assessments of brain iron levels [413]. The results indicated that although DFP effectively decreased brain iron accumulation, it also accelerated cognitive deterioration and disease progression in AD patients, highlighting the delicate balance of brain iron in physiological function and the challenges of direct targeting [413].

In clinical research on PD, two double‐blind, placebo‐controlled trials (NCT00943748, NCT01539837) have demonstrated that DFP (20–30 mg/kg/day) significantly reduced iron content in the SN and led to slight motor symptom improvement after 6–12 months [370, 414]. However, a Phase II clinical trial (FAIRPARK‐II, NCT02655315) showed that 36 weeks of DFP treatment provided no clinical benefit and was associated with worsened PD rating scale scores at the 9‐month mark in early‐stage PD patients [415]. These mixed results underscore the need for careful evaluation of the risks and benefits of DFP in PD treatment [600].

### 4.2 Therapeutics Targeting Copper Homeostasis

Therapeutic strategies targeting copper dyshomeostasis are increasingly being explored for NDDs, encompassing approaches from copper ionophores and MPACs to specific chelators and natural agents (Table 3).

#### 4.2.1 Copper Ionophore

The copper ionophore elesclomol (ES) has shown great potential to mitigate neurodegenerative pathology and improve survival in a mouse model of MD, by which ES mediates copper transport into mitochondria, consequently raising COX levels within the brain [386]. In a *VPS13A‐D* mutant yeast model (*S. cerevisiae vps13Δ*) of a rare neurological disorder, Soczewka et al. showed that the application of copper ionophores, including ES, disulfiram, and sodium pyrithione, significantly alleviated the impairments [387]. Another copper ionophore glyoxalbis‐[N4‐methylthiosemicarbazonato]Cu^2+^, also known as Cu^II^(gtsm), has shown promising results in ameliorating the cognitive impairments observed in both the APP/PS1 and rTg4510 mouse tauopathy models [601]. Mechanistically, in both models, Cu^II^(gtsm) treatment leads to a significant reduction in biomarkers associated with tau pathology and a substantial increase in the structural component of the tau phosphatase PP2A [385].

#### 4.2.2 Copper Chelator

One promising copper chelating agent is diacetylbis(*N*(4)‐methylthioaminocarbonyl) copper(II), also known as Cu^II^(atsm), an orally bioavailable molecule that can cross the BBB. Traditionally exploited in cellular imaging for hypoxic tissue labeling, Cu^II^(atsm) also demonstrates specific accumulation in the striatum of PD patients. In several PD mouse models, Cu^II^(atsm) is capable of rescuing the loss of dopaminergic cells, improving motor dysfunction, and extending survival rates [397]. Cu^II^(atsm) can also inhibit αSyn nitration and fibrosis, and modulate copper delivery as well as expression of proteins related to copper metabolism [602]. Cu^II^(atsm) exhibits neuroprotective properties in ALS by neutralizing protein nitration and oxidation [603]. It donates copper to the mutant form of SOD1, which increases the amount of fully copper‐bound SOD1, enhancing motor performance and survival in ALS mouse models [604]. Moreover, oral administration of Cu^II^(atsm) can notably enhance SOD1 enzymatic activity, postpone the onset of paralysis, and extend the lifespan of mice with ALS [605].

DPA was the first oral copper chelator to be introduced for therapy in WD, followed by Trientine (TETA 2HCl) as an alternative with a higher safety profile [606]. Trientine, initially approved in 1969 for WD patients who are intolerant to DPA, is now a widely used alternative. Overall, the safety profile of Trientine is more favorable compared to DPA, with four times fewer side effects reported. In 2018, TETA 4HCl, an alternative formulation, was approved under the trade name Cuprior [606].

In addition, coadministration of CuCl_2_ and the lipophilic copper chelator *N*,*N*‐dimethyldithiocarbamate in an MD mouse model led to significantly increased brain copper levels and an extension of lifespan [607]. Furthermore, the groundbreaking Cu^2+^‐specific chelator TDMQ20 has achieved remarkable success in completely reversing cognitive and behavioral defects in distinct mouse models replicating the different stages of AD [396]. Remarkably, it also counteracts oxidative stress induced by copper–Aβ complexes within the cortical region [396].

#### 4.2.3 Metal–Protein Attenuating Compound

MPACs are a library of compounds capable of inhibiting Zn^2^^+^‐ and Cu^2^^+^‐induced Aβ oligomerization. MPACs refer to a class of hydrophobic, low‐affinity metal complexes. Unlike high‐affinity therapeutic chelators, MPACs do not interfere with essential transition metal homeostasis [416]. MPACs are featured by the 8‐HQ scaffold, a heterocyclic compound characterized by a moderate metal‐binding affinity; several 8‐HQ derivatives have been developed over the years.

Clioquinol (5‐chloro‐7‐iodo‐8‐hydroxyquinoline) was among the pioneering MPACs to enter clinical trials. Despite early encouraging outcomes in a small cohort of patients with moderately severe AD, the studies were halted because of the toxic side effects that emerged [608].

PBT2 (5,7‐dichloro‐2‐[(dimethylamino)methyl]‐8‐hydroxyquinoline), a second‐generation MPAC, is a more potent copper/zinc ion ionophore, with higher solubility and BBB permeability than clioquinol [609]. PBT2 can significantly decrease the AD pathology of AD model mice, resulting in marked improvements in synaptic function and learning memory [608]. The anti‐AD effects of PBT2 are mediated through the modulation of brain metal ion concentrations and the chelation of copper and zinc ions away from Aβ [610]. In addition, PBT2 improves motor function, extends life span, and reduces paralysis in HD mice [393].

Another notable example is 8‐hydroxyquinoline‐2‐carboxaldehyde isonicotinoyl hydrazone (INHHQ), which can efficiently compete with αSyn for copper binding. INHHQ has inhibitory effects on αSyn aggregation both in vitro and in cells [394]. Furthermore, INHHQ is safe for in vivo injection in mice and has the capacity to cross the BBB [394].

Together, the 8‐HQ scaffold has served as a versatile platform for developing copper‐modulating agents, as its hydroxyl group and positions 5, 7, and 2 can be strategically functionalized to optimize properties such as metal‐binding capacity, complex stability, and cellular uptake, exemplified by the dimethylaminomethyl side chain at the 2‐position in PBT2 [611, 612]. The structure–activity relationship (SAR) of copper‐8‐HQ complexes hinges on several key factors. First, the electronic effect, where electron‐withdrawing substituents on the quinoline ring can stabilize the copper center and enhance redox activity, as seen in clioquinol's cytotoxicity [613]. Second, the fine‐tuning of ligand lipophilicity to balance cellular uptake and toxicity [614]. Third, the position‐specific impact of substituents, with the five‐ and seven‐positions critically influencing metal binding [608]. Lastly, the ligand‐to‐metal stoichiometry, where evidence points to a 2:1 complex as the key active species [599].

#### 4.2.4 Copper–Protein Binding Agent

Tetrathiomolybdate (TTM) promotes the biliary excretion of copper and has some ability to traverse the BBB. Compared to DPA, TTM shows a significantly reduced rate of neurologic decline (less than 5%). It functions by a distinctive mechanism involving the formation of stable ternary complexes [615]. Notably, TTM exhibits a rapid onset of action, restoring normal copper homeostasis within weeks of treatment, without elevating serum “free copper” [616], and reducing intestinal copper uptake to a clinically significant degree [402].

Bis‐choline TTM, also known as ALXN1840, is an investigational first‐in‐class oral copper protein‐binding agent. ALXN1840 is a TTM derivative with greater stability and bioavailability, making it more advantageous for clinical applications of treating WD [403]. In clinical trials, ALXN1840 has been shown to rapidly reduce serum levels of non‐Cp Cu by forming a ternary complex with copper and albumin, reduce liver and blood copper levels through biliary excretion, improve neurological symptoms, and stabilize liver function in WD patients [403]. These properties make ALXN1840 a promising new option for the treatment of WD.

#### 4.2.5 Natural Compounds

A multitude of studies have highlighted the neuroprotective properties of various natural compounds through the modulation of copper metabolism or levels [507]. In particular, polyphenols, such as myricetin, apigenin, quercetin, and EGCG, have demonstrated potential neuroprotective effects, which include reducing oxidative stress, decreasing apoptosis, inhibiting inflammation, or minimizing the formation of toxic protein aggregates [507]. Examples of representative natural products for different NDDs are presented in Table 3.

Mechanistically, these polyphenolic natural products like myricetin and EGCG, potently chelate copper ions with high affinity, directly forming stable complexes with Cu^2^^+^/Cu^+^ to interrupt copper redox cycling and the resulting ROS generation, thereby preventing copper‐induced Aβ aggregation and oxidative damage [617, 618]. The neuroprotective effects of these substances are largely driven by their potent antioxidant activity, often exceeding the effects of mere metal chelation. Unlike MPACs, which focus on precise metal redistribution, these polyphenolic natural products provide broader neuroprotection through multitarget antioxidant synergy [619]. Considering that MPACs and natural products represent two highly promising therapeutic approaches with different mechanisms of action for modulating copper homeostasis, future optimizations of the copper‐modulating agents may involve combining the advantages of both strategies. This could include constructing bifunctional MPAC‐polyphenol hybrid molecules [507].

#### 4.2.6 Relevant Clinical Trials

Most of the reported clinical trials of interventional therapies for copper homeostasis have been in patients with WD, with a small number in patients with AD, PD, ALS, and HD (Table 4).

##### 4.2.6.1 PBT2 in AD and HD

Clinical trials of the second‐generation MPAC PBT2 have indicated its potential application in NDDs. In the context of AD, a Phase IIa study (NCT00471211) showed that PBT2 was safe for a 12‐week usage and successfully reduced CSF Aβ_42_ levels [416, 417]. However, the overall therapeutic efficacy of PBT2 was limited, as cognitive improvement was restricted to only two specific executive function tests in the 250 mg dose group, without significant benefits evident in broader cognitive scales or memory functions [416]. Additionally, no effects on plasma biomarkers of AD were observed [416]. PBT2 also did not affect serum Cu^2+^ concentrations. For HD, a Phase II trial (NCT01590888) found PBT2 to be safe and well‐tolerated over a period of 26 weeks; however, the efficacy signal was weak and ambiguous [421]. Specifically, the high‐dose (250 mg) group only demonstrated significant improvement in one executive function test compared to placebo, while the composite cognition score, along with the other seven individual cognitive tests, did not show significant benefit [421]. Therefore, the two aforementioned clinical trials failed to establish a clear clinical efficacy of PBT2, underscoring the translational challenges encountered when attempting to convert mechanistic exploration into clinical validation.

The experience with PBT2's clinical trial highlights the challenge of achieving adequate disease modification in vivo, which is associated with low specificity and reliance on an ionophoric mechanism [620]. Furthermore, therapeutic success is contingent upon precision targeting, which can be facilitated through improved specificity or the use of multimodal agents [621]. First, the hydroxyquinoline chelation module of PBT2 can be covalently linked with the *N*,*N*‐dimethylaniline recognition unit of Aβ imaging agents to construct bifunctional molecules that possess both metal‐chelating and Aβ‐specific binding properties, thereby improving synergistic targeting of the pathological core of NDDs [622]. Second, a prodrug strategy can be adopted, where the active site is “masked” to achieve selective activation and delivery, thereby minimizing off‐target effects and systemic toxicity toward healthy tissues [611]. Third, improvements to the physicochemical properties of the molecules can be achieved by conjugating them with hydrophilic carriers to increase water solubility, utilizing specific transport systems for targeted delivery to the brain, and limiting passive diffusion and nonspecific distribution, thereby enhancing the interventive efficacy within the CNS while concurrently reducing the risks associated with peripheral metal chelation [623, 624]. Through these strategies, the selective intervention ability of PBT2 and similar compounds in addressing the aggregate–metal complex pathological system can be enhanced, while their potential off‐target toxicity is minimized.

##### 4.2.6.2 Cu^II^(atsm) in PD and ALS

Clinical studies on Cu^II^(atsm) show promise in NDDs. In PD, a Phase I trial (NCT03204929) found 4‐month treatment to be well‐tolerated and associated with symptom improvement in early‐stage patients [625]. For ALS, a Phase I study (NCT02870634) assessed its safety and pharmacokinetics, while a Phase II efficacy and safety trial (NCT04082832) has been completed with results pending.

##### 4.2.6.3 TTM, Trientine, and ALXN1840 in WD

Clinical studies investigating copper chelators for WD indicate both their efficacy and safety. Trial NCT00004339 suggests that TTM can reduce copper accumulation and improve symptoms [418]. In a completed trial (NCT01472874) involving Trientine, it was reported that patients remained clinically stable, with only transient fluctuations in liver enzyme levels observed. Additionally, a retrospective study (NCT03299829) was conducted to evaluate the effects of Trientine on liver function and urinary copper excretion, however its final results are still pending.

Two clinical trials on ALXN1840 for WD demonstrated its efficacy. A Phase II study (NCT02273596) reported a 71% therapeutic success rate [418]. A subsequent Phase III trial (NCT03403205) further confirmed ALXN1840's superiority over standard care, showing rapid and sustained copper mobilization over 48 weeks, indicating its potential to improve clinical symptoms and quality of life.

In summary, although some clinical trials have been conducted in recent years, the safety and efficacy results have not been satisfactory. The currently available therapeutic strategies targeting copper dyshomeostasis have not yet achieved breakthroughs, suggesting that more efforts should be made to develop next‐generation copper‐targeted strategies for the treatment of NDDs.

### 4.3 Artificial Intelligence in Iron and Copper Homeostasis Research

#### 4.3.1 AI‐Driven Prediction of Iron/Copper–Protein Binding Sites

Metalloproteins, which bind metals like Fe and Cu, comprise 30%–40% of all proteins and are critical for catalysis, electron transfer, oxygen transport, structural stability, and regulation. Common motifs include cysteine/His‐rich clusters for Fe–S centers and His/cysteine/methionine combinations for Cu. The prediction of MBSs in proteins can utilize various computational methods. These methods are primarily categorized into two main types: sequence‐based approaches and structure‐based approaches. The use of AI for predicting iron and copper binding motifs or sites in proteins is an actively advancing field. ML and deep learning (DL) are increasingly being incorporated into these methodologies to enhance the accuracy and scalability of predictions [626].

Among various ML algorithms, Random Forest represents one of the most effective models for the prediction of MBS. For example, Nguyen et al. analyzed the backbone atom geometric features of metal‐binding amino acids within the entire protein data bank and then applied a Random Forest decision tree algorithm for the identification of MBS, achieving remarkable results [627]. Another prominent model is the support vector machine (SVM), exhibiting robust performance in predicting binding sites of Fe/Cu, possessing both specificity and generalization ability [626]. For example, FINDSITE‐metal provides a reliable confidence assessment for Fe/Cu binding sites via SVM and Bayesian classifiers [628].

More recently, the application of DL models further improved the performance on MBS prediction. For instance, convolutional neural network (CNN) enables efficient identification of binding sites for Fe and Fe–S cluster [629], 3D DL ensemble model demonstrates exceptional accuracy in predicting Fe binding sites [630], and graph neural network (GNN) supports the accurate identification of MBS, without knowing the coordinating residue types and side‐chain information [631]. In addition, multilayer perceptron (MLP)‐based mebipred is capable of identifying the binding propensity of Cu and Fe (heme/Fe–S) in an alignment‐free manner, with a much better accuracy than other sequence‐based prediction methods [632]. BioBrigit, a hybrid CNN trained on 3D point clouds and a knowledge‐based tool, was developed to allow for computational characterization of diffusion paths of metals in proteins [633].

Overall, these advancements have significantly promoted MBS prediction, which is a key prerequisite for the design of novel metalloproteins with customized binding sites.

#### 4.3.2 Virtual Screening to Discover Novel Iron/Copper Modulators and Optimize Chelator Structures

VS methods, including structure‐based and ligand‐based approaches, have accelerated hit identification from vast libraries, and reduced wet‐lab costs in the general field of drug discovery. VS is increasingly applied to discover and optimize iron and copper modulators for treating NDDs, although direct and successful application of VS in this domain remains limited. Optimization focuses on improving selectivity, BBB permeability, oral bioavailability, and multifunctionality.

Below, we will illustrate this using the VS of copper chelating agents. Chaparro et al. developed a VS pipeline that combined quantum chemical calculations, an isodesmic classification method, and quantitative structure–activity relationship (QSAR) modeling to assess the chelating, drug likeness, and redox properties of copper ligands. They managed to identify four molecular scaffolds as promising Cu modulators, which could serve as potential drug candidates for AD treatments [634]. Holzhauer et al. combined the NMR‐based screening and VS to guide the synthesis of heteroleptic copper complexes in different complexation manners [635]. Michael et al. used SWISS ADME (absorption, distribution, metabolism, excretion) to assess the compounds’ drug‐likeness and ADME properties, Gaussian 09 W for density functional theory (DFT) calculations, and HEX 8.0 with Discovery Studio Visualizer for molecular docking to clarify the binding modes and efficiencies of copper chelators [636].

Therefore, the integration of VS with wet‐lab validation not only accelerates the discovery of novel Fe/Cu modulators and the precise optimization of chelator structures but also provides innovative approaches for targeted disease therapy.

#### 4.3.3 Big Data Analytics to Identify Correlations Between Iron/Copper‐Related Genetic Variants

Big data analytics have been applied to detect correlations between metal‐related genetic variants and NDDs. While there is little research on iron, the associations between copper‐related genetic variants and NDDs such as AD, WD, and ALS have been explored.

Squitti et al. conducted a meta‐analysis of 56 clinical studies, which suggests that the AG haplotype of the *ATP7B* gene is more prevalent in AD patients [637]. Individuals with this genotype have an excessive accumulation of non‐Cp Cu, suggesting an increased susceptibility to copper imbalance. This imbalance may accelerate the development of AD through mitochondrial dysfunction, imbalances in other essential metals (e.g., iron and zinc), and dysfunction of CuBPs, which in turn lead to oxidative stress and inflammation [637].

Since WD is primarily caused by mutations in the ATP7B gene, Zhou et al. collected 253 rare synonymous variants of ATP7B and determined the splicing outcome resulting from these variants [638]. Among the 85 tested variants, 11 were found to cause partial exon deletion or skipping. These aberrant splicing events may lead to loss of ATP7B function, subsequently triggering WD. This study provides crucial evidence for the genetic diagnosis of WD, particularly for patients carrying synonymous variants of uncertain significance [638].

In some sporadic cases, rare missense variants in familial ALS causative genes are present, but their pathogenicity often remains unclear. Hatano et al. developed an ML method called MOVA, based on structural and locational information from AlphaFold2, to predict the pathogenicity of variants in ALS‐related genes [639]. MOVA demonstrated excellent performance for five ALS‐related genes, including SOD1 [639].

Through whole‐exome sequencing analysis, Hsiao et al. identified a heterozygous *KCND3* c.1256G > A (p.R419H) variant in a PD patient. Multiple bioinformatic analyses, including the utilization of the gnomAD database, confirmed its pathogenic nature, demonstrating its association with neurodegenerative disorders characterized by brain iron accumulation [640].

#### 4.3.4 Neuroimaging AI for Noninvasive Monitoring of Brain Iron/Copper Levels

Noninvasive quantification of brain copper trafficking or pools holds promise as a biomarker for early diagnosis, disease progression tracking, and therapy response assessment. AI applied to positron emission tomography (PET) data from copper‐radiotracer studies for noninvasive monitoring of brain copper is emerging [641].

A copper sensor, F‐NpCu1, has been developed that combines fluorescent imaging with potential PET imaging capabilities. It can quantify differences in copper levels in mouse and postmortem human brain tissue. Following intravenous injection, its biodistribution matched tissue copper content, and it demonstrated the ability to cross the BBB. This sensor enables real‐time detection of copper levels in the living brain through intravital endoscopic imaging, providing an in vivo copper detection tool for CNS disorders and other copper dysregulation‐associated conditions [642].

NDDs involve a normoxic process and impaired electron transport chain activity, thereby triggering a compensatory hyper‐reductive state. This promotes abnormal retention of compounds like Cu‐ATSM in affected neural tissues [643], supporting its potential as a tracer for NDDs. PET imaging with ^62^Cu/^64^Cu‐ATSM has revealed distinct regional uptake patterns correlating with disease progression across multiple NDDs [644, 645, 646, 647]. Torres et al. demonstrated that the brain uptake and distribution of ^64^Cu‐GTSM in AD models are significantly altered [648, 649], suggesting its usage as a diagnostic tool for AD.

Concurrently, several novel targeted ^64^Cu agents have been developed. Cho et al. designed a bifunctional chelator linked to two Aβ‐targeting fragments, enhancing both binding affinity and BBB penetration for AD imaging [650]. Nam et al. reported a fluorescent conjugate (^64^Cu‐ATSM‐FITC), which enables in vivo imaging of endogenous H_2_S, offering a tool for neuroinflammation diagnosis [651]. Shojaei et al. developed a ^64^Cu‐labeled antibody tracer targeting the triggering receptor expressed on myeloid cells 2 (TREM2) protein, a surrogate marker for microglia activation, enabling specific neuroinflammation imaging in AD models and human tissues [652].

The integration of AI further supports this field. Adeli et al. developed a DL model called swinUNETR for attenuation and scatter correction in ^64^Cu PET imaging. Using a transfer learning approach pretrained on gallium‐68 images and fine‐tuned on ^64^Cu data, the model generated results highly consistent with standard CT‐based corrections, demonstrating feasibility for future standalone PET systems to reduce patient radiation exposure [653]. This AI advancement lays crucial groundwork for the refined application of copper‐based radiotracers in NDDs.

## 5 Conclusion and Perspectives

Iron and copper ions are essential trace elements involved in various biological processes; however, their redox‐active nature also makes them a potential source of oxidative stress, which can contribute to neurodegeneration [654]. Through better understanding the role of these ions in various biological processes associated with the onset and development of NDDs, researchers can potentially develop new treatments for NDDs and other diseases characterized by the dysregulation of metal ion metabolism, accumulation of misfolded proteins, and oxidative damage. The expanded knowledge of the roles of iron and copper in modulating key proteins and cellular processes is crucial for developing targeted therapies that can correct metal balance in the CNS, prevent the formation of toxic protein aggregates, and protect neurons from oxidative stress. This could involve the development of metal‐binding molecules that can sequester excess iron or copper or modulate protein activity at specific sites, thereby influencing their function and potentially slowing or reversing the progression of NDDs.

Understanding the relationship between iron/copper and their respective binding proteins, especially IDPs, is crucial for the development of therapeutic strategies for NDDs. The distinctive architecture of MBSs, which often involves interactions between side‐chain groups such as His imidazoles and cysteine thiols with the metal ion, represents a common feature in many metal‐binding proteins. The recently introduced concept of “metalloallostery” offers an intriguing perspective in the field of bioinorganic chemistry and metallobiology [655]. Targeting the interactions between iron/copper and IDPs may present a novel strategy to mitigate protein misfolding and aggregation, thereby potentially slowing or halting disease progression. Further research is needed to investigate these interactions in greater detail and to translate these findings into clinical applications.

Although chelation therapy has shown neuroprotective potential in preclinical studies, its clinical translation remains hindered by three main challenges. First, the lack of selectivity in current therapies results in the depletion of essential metals necessary for physiological functions, alongside the removal of pathological deposits. Second, the pharmacological complexity of current agents complicates the management of compartmentalized intracellular metal imbalances, with some agents potentially forming unstable complexes that may exacerbate oxidative stress. Third, the deficiency of assessment tools and the lack of biomarkers to monitor real‐time cerebral metal dynamics hinder the ability to correlate therapeutic intervention with clinical outcomes [610]. As a result, future strategies should focus on transitioning from mere metal clearance to the precise modulation of metal homeostasis. Achieving this progression will require the development of agents with improved BBB permeability and tissue targeting, in conjunction with early diagnostic approaches, to restore impaired metal transport and utilization systems rather than relying solely on indiscriminate chemical sequestration [610, 656].

The ongoing research into the roles of iron and copper in neuronal biology is poised to reveal new insights and therapeutic targets. Here are some key points and future directions that could be considered in this pursuit. (1) Unraveling iron and copper biology: Understanding the intricate biology of both iron and copper within the CNS will likely uncover new mechanisms that could be targeted therapeutically. (2) Discovery of new targets: As research progresses, it may identify novel molecular targets within iron‐ and copper‐related pathways that are dysregulated in the brain. These targets could be proteins involved in metal transport, storage, or proteins that are regulated by iron or copper ions. (3) Development of metal‐focused drugs: With a better understanding of the roles and functions of iron and copper in the CNS, the development of drugs that are capable of modulating iron/copper metabolism or levels could become more precise. (4) AI integration: AI integrates multiomics data to uncover molecular links between metal homeostasis and disease, assists in designing and optimizing novel chelating agents with high selectivity and BBB permeability, and predicts their efficacy, toxicity, and metal selectivity to reduce preclinical development risks. AI further supports patient stratification and clinical trial design, promoting precision drug application. These might include chelators for iron or copper, ionophores, or compounds that modulate their activity at specific sites within proteins.

In conclusion, this review offers a systematic synthesis of the relationship between iron and copper homeostasis and NDDs from the perspectives of metal metabolism, metal–protein interactions, and metal–ion‐focused intervention strategies, thus providing a comprehensive reference for therapeutic advancements and future research directions for NDDs.

## Author Contributions

**Xin Liu**: investigation, visualization, writing – original draft, writing – review and editing. **Lin Jia**: investigation, visualization, writing – review and editing. **Kerong Wu**: investigation, writing – review and editing. **Mo Chen**: investigation. **Jichao Sun**: validation. **Chuanbin Yang**: formal analysis. **Chengchao Xu**: formal analysis. **Jie Sun**: supervision, writing – review and editing. **Jigang Wang**: supervision, writing – review and editing, funding acquisition**. Lingyun Dai**: supervision, writing – review and editing, funding acquisition. All the authors have read and approved the final version of the manuscript.

## Ethics Statement

The authors have nothing to report.

## Conflicts of Interest

The authors declare no conflicts of interest.

## Data Availability

The authors have nothing to report.
